# Dynamic 3D-Network Coating Composite Enables Global Isolation of Phosphopeptides, Stepwise Separation of Mono- and Multi-Phosphopeptides, and Phosphoproteomics of Human Lung Cells

**DOI:** 10.3390/biom15060894

**Published:** 2025-06-18

**Authors:** Linlin Liu, Zhenhua Chen, Danni Wang, Weida Liang, Binbin Wang, Chenglong Xia, Yinghua Yan, Chuanfan Ding, Xiaodan Meng, Hongze Liang

**Affiliations:** 1 Key Laboratory of Advanced Mass Spectrometry and Molecular Analysis of Zhejiang Province, School of Materials Science and Chemical Engineering, Ningbo University, Ningbo 315211, China; liulinlin1205@163.com (L.L.); 13965866085@163.com (D.W.); liangweida94@gmail.com (W.L.); 13453635484@163.com (B.W.); x17816980649@163.com (C.X.); yanyinghua@nbu.edu.cn (Y.Y.); dingchuanfan@nbu.edu.cn (C.D.); 2 Health Science Center, Ningbo University, Ningbo 315211, China; czhpfsh@163.com

**Keywords:** immobilized metal-ion affinity chromatography, functionalized ionic liquid, polymer brush, phosphopeptide enrichment, lung cancer

## Abstract

Protein phosphorylation is one of the most common and important post-translational modifications (PTMs) and is highly involved in various biological processes. Ideal adsorbents with high sensitivity and specificity toward phosphopeptides with large coverage are therefore essential for enrichment and mass spectroscopy-based phosphoproteomics analysis. In this study, a newly designed IMAC adsorbent composite was constructed on the graphene matrix coated with mesoporous silica. The outer functional 3D-network layer was prepared by free radical polymerization of the phosphonate-functionalized vinyl imidazolium salt monomer and subsequent metal immobilization. Due to its unique structural feature and high content of Ti^4+^ ions, the resulting phosphonate-immobilized adsorbent composite G@mSiO_2_@PPFIL-Ti^4+^ exhibits excellent performance in phosphopeptide enrichment with a low detection limit (0.1 fmol, tryptic β-casein digest) and superior selectivity (molar ratio of 1:15,000, digest mixture of β-casein and bovine serum albumin). G@mSiO_2_@PPFIL-Ti^4+^ displays high tolerance to loading and elution conditions and thus can be reused without a marked decrease in enrichment efficacy. The captured phosphopeptides can be released globally, and mono-/multi-phosphopeptides can be isolated stepwise by gradient elution. When applying this material to enrich phosphopeptides from human lung cell lysates, a total of 3268 unique phosphopeptides were identified, corresponding to 1293 phosphoproteins. Furthermore, 2698 phosphorylated peptides were found to be differentially expressed (*p* < 0.05) between human lung adenocarcinoma cells (SPC-A1) and human normal epithelial cells (Beas-2B), of which 1592 were upregulated and 1106 were downregulated in the cancer group. These results demonstrate the material’s superior enrichment efficiency in complex biological samples.

## 1. Introduction

By virtue of various post-translational modifications (PTMs), proteins perform diverse functions that regulate a wide range of biological processes in living organisms. Among PTMs, reversible protein phosphorylation, controlled by kinases and phosphatases, is the most extensively studied and plays a pivotal role in numerous cellular events, including proliferation, survival, protein functions, protein–protein interactions (PPIs), and signaling networks [1,2]. To date, phosphoproteomics has found increasingly widespread applications in life sciences and related fields [1,3,4]. Abnormal protein phosphorylation is linked to many diseases, such as cancers, neurological disorders, and diabetes. Gaining insights into dynamic phosphorylation events contributes to the identification of diagnostic biomarkers and drug targets [5]. Mass spectrometry (MS)-based technology coupled with liquid chromatography has developed fast over the past decades in phosphoproteomics due to inherent merits such as extra high detection sensitivity, accuracy, and rapidity and can overcome the drawbacks of the limited availability, poor reproducibility, low sensitivity, and expensive costs of antibody-based protocol for large-scale application [6,7,8].

It is known that high-quality phosphoproteomic analysis is highly dependent upon the enrichment quality of phosphorylated peptides. Ideal enrichment materials should have multiple advantages such as low detection limits, high selectivity and capacity, and broad phosphopeptide coverage. Although many strategies and several combinations are proposed, immobilized metal affinity chromatography (IMAC) has been regarded as one of the most popular protocols [3]. The number of IMAC materials has greatly increased over the past decade [9,10]. However, traditional IMAC materials, mainly relying on a single influencing factor or simple combinations, cannot effectively enrich and separate target phosphopeptides for high-quality phosphoproteomic analysis due to severe interferences from other biomacromolecules in complex real biological samples, the vulnerability and sub-stoichiometry of phosphorylated peptides, and the low ionization rate of phosphopeptides in MS measurements [11].

Functional polymeric materials capable of responding to various physical and chemical changes or the presence of biomolecules in the surrounding environments have been used in a variety of fields. Thus, the potential of the polymer-modified IMAC enrichment materials has attracted much attention in life science research [11]. Zou and coworkers constructed novel IMAC-M^n+^ adsorbents based on a monodisperse polymeric microsphere [12]. Polydopamine-based IMAC material was reported and applied to the enrichment of endogenous phosphopeptides in human serum [13]. Lu’s group introduced poly(ethylene glycol methacrylate phosphate)-immobilized Ti^4+^ as the functional polymeric layer to enrich phosphorylated peptides [14]. With poly(glycidyl methacrylate) microspheres as seeds, Ti^4+^-immobilized poly(vinylphosphonic acid-co-ethylene dimethacrylate) beads were prepared [15]. Liu reported a four-armed PEO-based Ti^4+^ adsorbent [16]. Jia et al. suggested a facile protocol to prepare a magnetic IMAC composite coated with polydopamine-polyethyleneimine [17]. Yan and Ding recently prepared a poly(ionic liquid) microsphere matrix (MBA-VimDOPA-Ti^4+^) via the copolymerization of methylenebisacryamide and vinylimidazolium derivatives [18]. In particular, Tao and coworkers proposed another enrichment strategy by which target phosphopeptides in a complex mixture were selectively isolated by forming chemical bonding between phosphopeptides and soluble dendrimers [19]. Later, they reported a new adsorbent, termed polymer-based metal ion affinity capture (PolyMAC), showing high selectivity and sensitivity toward phosphopeptides [20]. However, although some polymer-based IMAC materials may exhibit intriguing prominence in some aspects, most of them so far have not shown overwhelming superiority over traditional IMAC adsorbents or metal oxide affinity chromatography (MOAC) counterparts [3].

We recently proposed a strategy by which graphene was surface-decorated with phosphonate-functionalized ionic liquid (PFIL) to prepare hydrophilic IMAC-Ti^4+^ materials [21,22]. These novel adsorbents were applied to the studies of Alzheimer’s disease (AD), and the phosphoproteomic results found a discrepancy in protein phosphorylation between amyloid precursor protein (APP)/presenilin 1 (PS1) and MAPT×P301S (microtubule-associated protein tau) transgenic mice in an early stage of AD without showing cognitive dysfunctions. These results suggest that differentially phosphorylated proteins may be used for early diagnosis [23]. Further phosphoproteomic and kinomic studies indicate that 9-methylfascaplysin, reported by us, exerts anti-AD action via the specific inhibition of rho-associated coiled-coil kinase 2 (ROCK2) in microglia and glycogen synthase kinase 3β (GSK3β) in neurons [24].

**Scheme 1:**
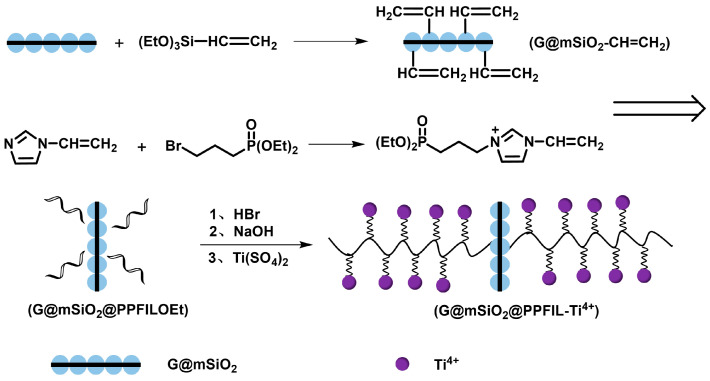
Schematic illustration of the synthetic procedure for preparation of the G@mSiO_2_@PPFIL-Ti^4+^ composite.

Inspired by these results, herein, we report a new GO-based composite that was coated by a layer of functional brush polymer. Phosphonate-functionalized vinyl imidazolium ionic liquid monomers were polymerized via free radical polymerization (Scheme 1). The high content of metal ions immobilized by dense phosphonate groups and less steric hindrance facilitate the capture of phosphopeptides, especially in low abundance, thus endowing the material with extra low detection limit, high selectivity, wide coverage of phosphorylated peptides, reusability, and practicality to implement an in-depth phosphoproteomic analysis of the real biological samples. Moreover, quantitative phosphoproteomics and bioinformatics have been applied to non-small-cell lung cancer (NSCLC), demonstrating the application potential of the adsorbent in the enrichment of phosphopeptides from complicated, real samples.

## 2. Materials and Methods

### 2.1. Materials and Chemicals

Detailed information is presented in the Supporting Information.

### 2.2. Characterization and Measurement

Detailed information is presented in the Supporting Information.

### 2.3. Preparation of G@mSiO_2_@PPFILOEt

Firstly, the starting materials 1-vinyl-3-(diethoxyphosphoryl) propylimidazolium bromide (ViEPPImBr) and mesoporous silica-coated graphene (G@mSiO_2_) were prepared according to the published methods, respectively [21,25]. Then, to 400 mg of G@mSiO_2_ dispersed in 30 mL of anhydrous toluene, we added vinyltriethoxysilane (0.44 mL) and triethylamine (44 μL). The mixture was then stirred and refluxed at 110 °C under a nitrogen atmosphere for 12 h. The supernatant was removed via centrifugation. The vinyl-modified precursor (G@mSiO_2_-CH=CH_2_) was collected and washed with ethanol several times and vacuum-dried before use. To a two-neck flask containing 100 mg of ViEPPImBr in 20 mL of chloroform, we added G@mSiO_2_-CH=CH_2_ (200 mg) and AIBN (8 mg) and dispersed them via sonication. This mixture was purged with nitrogen, stirred at room temperature for 1 h, and then heated at 75 °C for 3 h. The resulting solid (G@mSiO_2_@PPFILOEt) was separated via centrifugation, washed with chloroform and ethanol repeatedly, and vacuum-dried overnight at 85 °C.

### 2.4. Preparation of G@mSiO_2_@PPFIL-Ti^4+^

Hydrolysis of G@mSiO_2_@PPFILOEt (200 mg) was conducted in an acidic solution of HBr at 100 °C for 1 h. Neutralization with NaOH (pH = 10), washing with DI water to neutral, and dryness under vacuum at 100 °C afforded G@mSiO_2_@PPFIL-Na^+^. The prepared sodium salt composite (100 mg) was stirred with 30 mL of the Ti(SO_4_)_2_ solution (0.1 mol L^−1^) at room temperature for 2 h. The supernatant was removed via centrifugation, and the resulting composite G@mSiO_2_@PPFIL-Ti^4+^ was washed with DI water several times and vacuum-dried at 100 °C.

### 2.5. Sample Preparation

Generally, the digested solutions of standard proteins for enrichment evaluation were prepared according to our previous work [21,22], and the real samples including human serum and saliva were also prepared accordingly. Detailed information can be found in the Supporting Information.

For the study on human lung cells, one normal lung epithelial cell line (Beas-2B) and one lung adenocarcinoma cell line (SPC-A1) were used. After harvesting, the cells were lysed in SDT buffer and sonicated using a probe sonicator in an ice water bath to ensure complete cell disruption. The resulting cell suspension was then centrifuged at 14,000 rpm for 30 min to separate the soluble proteins. The supernatant was carefully collected, and the protein concentration was determined using the BCA protein assay. For protein digestion, the proteins were first reduced by incubating with DTT (0.1 M) at 37 °C for 2 h, followed by alkylation with IAA (0.25 M) at room temperature in the dark for 30 min. To quench the alkylation reaction, an additional DTT (0.1 M) was added and incubated for 10 min. The urea buffer was then diluted to 1.6 M using 50 mM TEAB buffer to create optimal conditions for trypsin digestion. Finally, the proteins were digested with trypsin at a 1:50 (*w*/*w*) ratio for 16 h at 37 °C. The resulting protein digest was lyophilized to dryness and stored at −20 °C for further analysis [26].

### 2.6. Enrichment of Phosphopeptides

Each portion (0.25 mg) of composite G@mSiO_2_@PPFIL-Ti^4+^ was ultrasonicated to ensure dispersion in 100 μL of loading buffer (50% ACN, 6% TFA, *v*/*v*). Then, β-casein digest (1 μL) was added to the above dispersion and the mixture was further oscillated at 37 °C for 30 min. The adsorbent was isolated by centrifugation at 1000 rpm for 3 min and washed by loading buffer two times, and finally with washing buffer (50% ACN, 0.1% TFA, *v*/*v*). The isolated adsorbent with captured phosphopeptides was eluted using 10 μL of the NH_4_OH solution (0.6 M) and oscillated at 37 °C for 15 min. A portion of the eluent (0.5 μL) was taken and then mixed with the matrix (0.5 μL) for analysis by MALDI-TOF MS. The protocol of phosphopeptide enrichment from the digested mixtures of β-casein and BSA and mixtures of digested β-casein, β-casein, and BSA was similar to the above-described process. To evaluate the reusability, effect of gradient elution, and batch-to-batch reproducibility of the G@mSiO_2_@PPFIL-Ti^4+^ composite, a sample of β-casein digest in the concentration of 2 pmol was employed. The procedures to enrich endogenous phosphopeptides from human saliva and serum were carried out according to a previous report [21].

The lyophilized digest of human lung cells (about 1 mg) was taken and dissolved in 200 μL of loading buffer (50% ACN, 6% TFA, *v*/*v*) and was mixed with 2 mg G@mSiO_2_@PPFIL-Ti^4+^ composite dispersed in 300 μL of loading buffer. The suspension was oscillated at 37 °C for 30 min and centrifuged at 1000 rpm. The supernatant was removed, and the adsorbent was washed with loading buffer and deionized water three times and treated with elution buffer (30 μL, 0.6 M NH_4_OH) under oscillation for 15 min to release the enriched phosphorylated peptides. The eluent was then lyophilized to dryness and stored at −20 °C before conducting nano-LC-MS/MS analysis.

### 2.7. MALDI-TOF MS Analysis

Details are included in the Supporting Information.

### 2.8. LC-MS/MS Analysis and Data Search

Detailed information is presented in the Supporting Information [17,27,28,29,30,31,32,33].

## 3. Results and Discussion

### 3.1. Preparation and Characterization of G@mSiO_2_@PPFIL-Ti^4+^ Composite

Our previous work clearly demonstrates that our strategy to enhance the enrichment of phosphopeptides is generally practicable for designing IMAC materials [21,22,34]. In addition to the remaining favorable features of hydrophilicity and strong metal binding ability, in this study, we intentionally increase the density of chelators by covering the matrix with a brush polymer bearing more metal coordination sites. The phosphonate-functionalized vinyl imidazolium monomer could be easily polymerized by AIBN. Thus, more metal ions can be immobilized by the chelation of the dense phosphonate groups, which is beneficial for capturing ultralow phosphorylated peptides from real samples. The synthetic route of the composite (G@mSiO_2_@PPFIL-Ti^4+^) is illustrated in Scheme 1.

**Figure 1:**
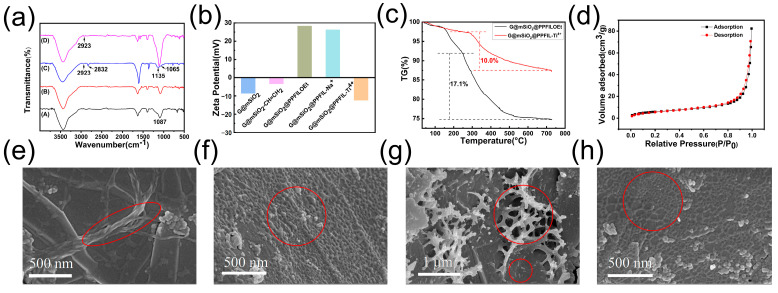
Structural characterization of composites. (**a**) The FT-IR of G@mSiO_2_ (A), G@mSiO_2_-CH=CH_2_ (B), G@mSiO_2_@PPFILOEt (C), and G@mSiO_2_@PPFIL-Ti^4+^ (D). (**b**) Bar graph of zeta potentials. (**c**) The TGA of G@mSiO_2_@PPFILOEt and G@mSiO_2_@PPFIL-Ti^4+^. (**d**) N_2_ adsorption−desorption isotherm of the G@mSiO_2_@PPFIL-Ti^4+^ composite and BET surface area. SEM images for G@mSiO_2_@PPFILOEt (**e**,**f**), and for G@mSiO_2_@PPFIL-Ti^4+^ (**g**,**h**).

The FTIR spectra of G@mSiO_2_, G@mSiO_2_-CH=CH_2_, G@mSiO_2_@PPFILOEt, and G@mSiO_2_@PPFIL-Ti^4+^ are illustrated in Figure 1a. The adsorption peak at 1087 cm^−1^ was assigned to the asymmetric stretching of Si–O–Si of silica and Si–OH deformation from silanol, while the adsorption of the vinyl group overlapped with that of the matrix. After polymerization, the characteristic peaks of P=O (1135 cm^−1^) and P–C (1065 cm^−1^) could be observed in G@mSiO_2_@PPFILOEt (Figure 1a(C)). Additionally, the peaks appearing at 2923 and 2832 cm^−1^ are assigned to C–H stretching of the saturated backbone and sidechains of the polymer. The disappearance of the peak at 2832 cm^−1^ verified the successful hydrolysis of ethyl phosphonates and immobilization of Ti^4+^ ions (Figure 1a(D)).

Then, the zeta potentials of the gradually modified intermediates and the final product were measured to prove the successful preparation of G@mSiO_2_@PPFIL-Ti^4+^ (Figure 1b). The negative zeta potential of G@mSiO_2_ was due to the abundant distribution of silicon hydroxyl groups, and surface modification with vinyl groups led to the potential change of up to −3.44 mV. The further polymerization process increased the zeta potential to 28.3 mV. A slight drop to 26.27 mV was found after the hydrolysis of ethyl phosphonates and neutralization with NaOH. Finally, after cation exchange, the zeta potential of G@mSiO_2_@PPFIL-Ti^4+^ decreased to −12.4 mV.

Thermal gravimetric analysis (TGA) under a nitrogen atmosphere was carried out with G@mSiO_2_@PPFILOEt and G@mSiO_2_@PPFIL-Ti^4+^ (Figure 1c). For G@mSiO_2_@PPFILOEt, the first weight loss (below 150 °C) was due to the release of the adsorption water, and the loss between 150 and 250 °C was ascribed to the residual ethoxy group of the silane coupling agent. A 17% weight loss was observed between 250 and 750 °C due to poly(ionic liquid) decomposition. Upon metal immobilization, the weight loss of G@mSiO_2_@PPFIL-Ti^4+^ slightly decreased to 10% due to the indecomposable metal residue under test conditions. The nitrogen adsorption–desorption behavior of the G@mSiO_2_@PPFIL-Ti^4+^ composite was also studied, as shown in Figure 1d. After multi-step modification, the specific area of G@mSiO_2_@PPFIL-Ti^4+^ was dramatically decreased to 20.66 m^2^ g^−1^ compared to 139.69 m^2^ g^−1^ of G@mSiO_2_ (Figure S1). This result also supported the fact that functional brush polymers were successfully constructed on the matrix G@mSiO_2_. The 3D network consisting of phosphonate-modified brush polymers bridged by dense Ti^4+^ ions spreads out into space, and this structural nature endows the composite with superior capability to enrich phosphorylated peptides. Thus, the decrease in the physical surface area does not affect the enrichment capacity (see below).

Both the surface composition and the surface morphology of an adsorbent play key roles in the enrichment performance of phosphorylated peptides. As shown in Figure 1, the SEM images exhibited markedly morphologically different textures of G@mSiO_2_@PPFILOEt and G@mSiO_2_@PPFIL-Ti^4+^ compared to uniformly coated G@mSiO_2_ (Figure S2) and the previously reported G@mSiO_2_-PFIL-Ti^4+^ [21]. Two morphological features, intertangled rope-like strands and small bumps, were found in G@mSiO_2_@PPFILOEt, as shown in Figure 1e,f. The formation of entangled strands may be attributed to the synergistic effects of the hydrophobic interactions within neighboring polymeric backbones and the electrostatic interactions between imidazolium cations and bromide anions, whereas the formation of bumps may be mainly caused by the electrostatic interactions between cations and anions coming from the same polymer. Upon immobilization, numerous Ti^4+^ ions served as bridging agents, which may have randomly crosslinked more polymers by forming Ti^4+^-phosphonate coordination, leading to the formation of coral reef-like structures (Figure 1g,h). Energy-dispersive X-ray (EDX) spectra of G@mSiO_2_@PPFILOEt and G@mSiO_2_@PPFIL-Ti^4+^ also confirmed the success of the multistep construction of the adsorbent (Figure S3a,b). The element contents of P and Ti were measured to be 2.80 wt% and 6.11 wt%, respectively, much higher than those found in G@mSiO_2_-PFIL-Ti^4+^ reported in our previous work (P and Ti: 1.26 wt% and 2.44 wt%, respectively). Furthermore, the content of Ti measured by inductively coupled plasma optical emission spectroscopy analysis (ICP-OES) was found to be as high as 85.62 mg g^−1^. These results demonstrate that the introduction of the functional 3D network in material design is rational and workable. Finally, the beneficial changes in the water contact angle of G@mSiO_2_@PPFILOEt (54°) and G@mSiO_2_@PPFIL-Ti^4+^ (21°) again supported our rationality in material design that polymer brush cations make the adsorbent more hydrophilic, resulting in the enhanced capture of phosphopeptides [35] (Figure S3c,d).

### 3.2. Application of G@mSiO_2_@PPFIL-Ti^4+^ Composite in Phosphopeptide Enrichment from Standard Protein Digests

All samples containing phosphopeptides were enriched under the loading buffer condition (50% ACN, 6% TFA, *v*/*v*), and the adsorbed phosphopeptides were released under the elution buffer condition (10% NH_4_OH). The results of the optimization process are illustrated in Figure S4. The experiments that followed were conducted under the optimal conditions unless otherwise stated. It could be noted that the phosphopeptides captured by this novel adsorbent coating with poly(ionic liquid) brush polymers were enriched under more acidic conditions and eluted under more basic conditions. The higher tolerance against caustic conditions makes it possible to dramatically suppress interferences by fine-tuning parameters, and thereby significantly enhances the detection limit and selectivity of phosphopeptide targets.

**Figure 2:**
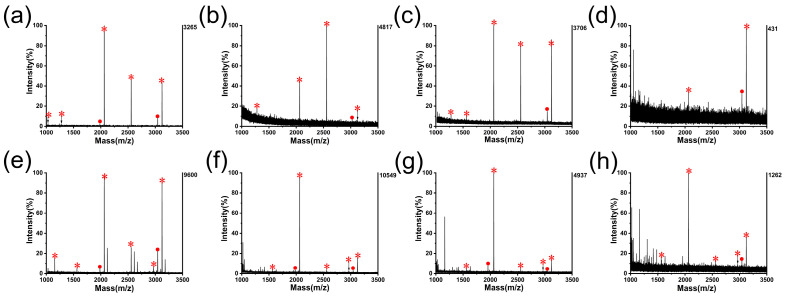
MALDI-TOF MS spectra of β-casein tryptic digest. Enrichment by G@mSiO_2_@PPFIL-Ti^4+^ composite (**a**) 10 fmol, (**b**) 1 fmol, (**c**) 0.5 fmol, and (**d**) 0.1 fmol. MALDI-TOF MS spectra of tryptic digest mixtures of β-casein and BSA enriched by G@mSiO_2_@PPFIL-Ti^4+^ at a molar ratio of (**e**) 1:1000, (**f**) 1:5000, (**g**) 1:10,000, and (**h**) 1:15,000. The peaks of phosphopeptides are marked with *, and the peaks of dephosphopeptides are marked with ●.

We first explored the detection limit of G@mSiO_2_@PPFIL-Ti^4+^ by using the tryptic digests of β-casein proteins in different concentrations. As shown in Figure 2a, four mono-phosphopeptides (*m*/*z* 1031, 1278, 2061, and 2556), one multi-phosphopeptide (*m*/*z* 3122) signals, and two dephosphorylated residues (1982, 3042) could be detected with a clean background when the β-casein digest (10 fmol) was enriched. The sequence assignment of these phosphopeptide peaks is listed in Table S1. Even when the β-casein digest was diluted with loading buffer to 0.1 fmol, two phosphopeptides and one dephosphorylated residue were detected (Figure 2d), demonstrating the excellent enrichment performance of G@mSiO_2_@PPFIL-Ti^4+^. The performance comparison with recently published results is listed in Table S2.

The ability to selectively enrich phosphorylated peptides from a pool of non-phosphopeptides is another key feature in evaluating adsorbent material. A series of digested samples of β-casein and BSA in different molar ratios were then prepared for selectivity evaluation. After being treated with G@mSiO_2_@PPFIL-Ti^4+^, five signals of phosphopeptides and two dephoshorylated residues in high intensities could be clearly observed when the molar ratio of β-casein and BSA was 1:5000 (Figure 2f). Even when diluted to 1:15,000, as shown in Figure 2h, five phosphopeptides and one fragment were still detected, exhibiting better selectivity than the adsorbent of this type previously reported by our group [21]. A comparison with other adsorbent materials regarding selectivity is also listed in Table S2. The improved specificity and selectivity of G@mSiO_2_@PPFIL-Ti^4+^ may be attributed to its unique structural features that combine high hydrophilicity, high acid and base tolerance, and high densities of phosphonate chelators and immobilized metal ions.

There are a large number of biomacromolecules in real biosamples, including non-phosphoproteins and phosphoproteins, which have a great impact on phosphopeptide enrichment. β-Casein serving as a phosphoprotein and/or BSA serving as a non-phosphoprotein were added to the digested β-casein to make simulative complicated biological samples. When a mixture of the digested β-casein and BSA (mass ratio, 1/1000) was enriched by G@mSiO_2_@PPFIL-Ti^4+^, four phosphopeptides and one dephosphorylated residue in high intensities could be detected with clean backgrounds, as shown in Figure S5a. A more complicated sample (β-casein digest/BSA/β-casein, molar ratio, 1/1000/1000) was employed. It was found that five phosphopeptides and one dephosphorylated fragment were also found with clean backgrounds (Figure S5b), exhibiting an excellent size-exclusion effect. Furthermore, we also determined the adsorption capacity of G@mSiO_2_@PPFIL-Ti^4+^ by using pyridoxal 5′-phosphate as a model compound. The capacity was 23.8 mg g^−1^, much higher than that of G@mSiO_2_-PFIL-Ti^4+^ (13.3 mg g^−1^). All the above results demonstrate that this new adsorbent modified with functionalized polymer brush cations exhibits enhanced overall performance compared to the previously reported counterpart [21].

**Figure 3:**
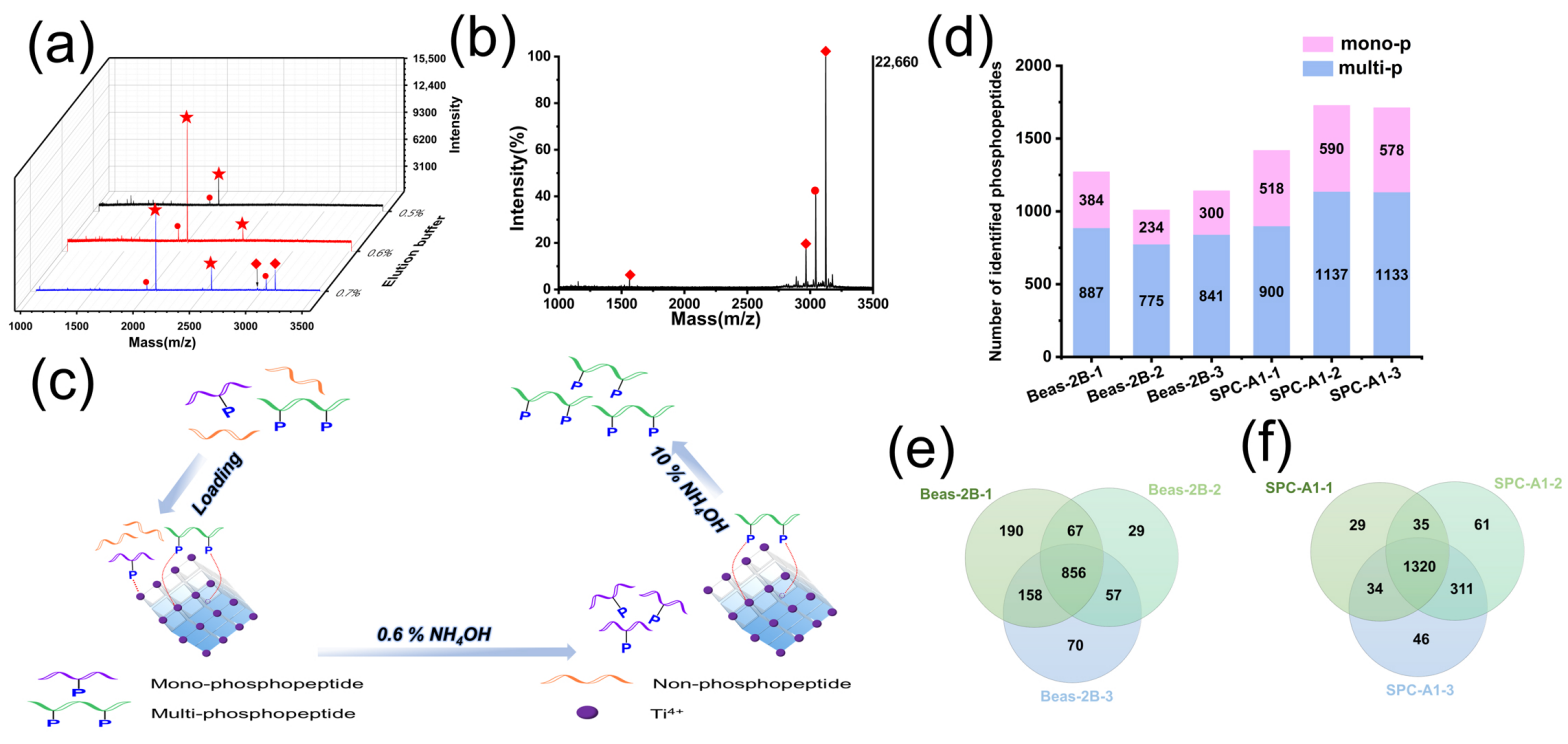
MALDI-TOF MS analysis of the fractional elution of mono- and multi-phosphopeptides of the β-casein digest (2 pmol). (**a**) Eluted with 0.5%, 0.6%, and 0.7% NH_4_OH, respectively. Mono-phosphopeptides, multi-phosphopeptides, and dephosphorylated residue are marked with asterisk, rhombus, and dot, respectively. (**b**) Consecutive elution with 0.6% and 10% NH_4_OH, and collection of the fractional part eluted by 10% NH_4_OH. Mono-phosphopeptides, multi-phosphopeptides, and dephosphorylated residue are marked with asterisk, rhombus, and dot, respectively. (**c**) Principle diagram of the enrichment and stepwise elution of phosphopeptides captured by G@mSiO_2_@PPFIL-Ti^4+^. (**d**) Bar graph showing the number of mono- and multi-phosphopeptides identified in Beas-2B and SPC-A1 cell lines. (**e**) Venn diagram representing the overlap of phosphopeptides identified in the Beas-2B triplicates. (**f**) Venn diagram representing the overlap of phosphopeptides identified in the SPC-A1 triplicates.

Researchers have found that due to different sizes and charges, any given protein with different sites and degrees of phosphorylation presents rich and unique spatial conformations, which can alter the protein–protein interactions and consequently affect its biological activities [36]. For example, abnormal hyperphosphorylation of the tau protein has been considered to be linked to Alzheimer’s disease [23,37,38,39,40,41,42,43], while the degree of phosphorylation of the myosin light chain (RLC) on Thr18 and Ser19 regulates adhesion and polarity in different ways [44]. In addition to the interference of non-phosphopeptides, however, mutual interference within mono- and multi-phosphopeptides could cause information loss in phosphopeptides in low abundance [45]. To address this problem, some strategies have been proposed to selectively enrich and separate mono- and multi-phosphopeptides [46,47,48,49,50,51]. In this work, we tentatively examine the feasibility of separating mono- and multi-phoshopeptides with G@mSiO_2_@PPFIL-Ti^4+^ by gradient elution, as shown in Figure 3. Gratifyingly, G@mSiO_2_@PPFIL-Ti^4+^ exhibited the selective release of mono- and multi-phosphopeptides. When the enriched phosphopeptides from the β-casein digest were first washed with 0.5% NH_4_OH, one mono-phosphospeptide (*m*/*z* 2061) was eluted. The two most common mono-phosphopeptides in high intensity (*m*/*z* 2061, 2556) were isolated with a clean background via treatment with 0.6% NH_4_OH. When further increasing the concentration of NH_4_OH to 0.7%, two multi-phosphopeptides (*m*/*z* 2966, 3122) began to be eluted out, in addition to two mono-phosphopeptides. In order to examine the possibility of selectively isolating multi-phosphopeptides, consecutive elution processes with different NH_4_OH concentrations were applied. The G@mSiO_2_@PPFIL-Ti^4+^ composite loaded with phosphorylated peptides was first eluted with 0.6% NH_4_OH twice to remove mono-phosphopeptides completely and then eluted with 10% NH_4_OH to allow for the complete elution of multi-phosphopeptides. Satisfyingly, three multi-phosphopeptides (*m*/*z* 1562, 2966, and 3122) and one corresponding dephosphorylated residue of a higher intensity were clearly observed without any interference of mono-phosphopeptides (Figure 3b).

Taken together, all of the above results demonstrate the superior performance of the G@mSiO_2_@PPFIL-Ti^4+^ composite in phosphopeptide enrichment. We can also provide insight into why this adsorbent has an improved performance. When this material was modified with multi-cationic polymer brushes on the surface, the enrichment capacity was no longer limited by the physical surface area and morphology of the GO substrate, as the multi-cationic polymer brushes construct a 3D network by coordinating to Ti^4+^ ions using their phosphonate groups. This cationic 3D network is hydrophilic and flexible yet robust in aqueous solution and has a much higher density of Ti^4+^ ions, which is necessary to enrich phosphopeptides in ultralow abundance. In principle, the resulting 3D network can be viewed as a hollow Rubik’s cube, consisting of an infinite number of small cubes, as shown in Figure 3c. During the process of extracting phosphorylated peptides, nano-sized small cubes remain stacked by coordinating bonds and flexible in aqueous solution, thereby preventing the interference of other large-sized biomolecules. On the other hand, as phosphate groups from phosphopeptides and phosphonate groups from polymer brushes have comparable bonding strength to Ti^4+^ ions, there is a fast exchange between these two groups to coordinate Ti^4+^ ions, forming a dynamic equilibrium. The multiphosphopeptides form relatively stronger interactions with polymer brushes due to more interaction sites with Ti^4+^ ions and thus were eluted only by concentrated NH_4_OH. Therefore, the selective isolation of mono-phosphopeptides and multi-phosphopeptides can be successfully achieved via gradient elution with NH_4_OH.

The recyclability of the G@mSiO_2_@PPFIL-Ti^4+^ nanocomposite was examined using β-casein tryptic digest. As observed in Figure S6, five, five, and three phosphopeptides were detected for the first, third, and sixth recycles, respectively. Additionally, it was also noted that with the increase in recycles, the intensities of mono-phosphopeptides (*m*/*z* 1278, 2061, 2556) decreased while the intensities of multi-phosphopeptides relatively increased. In the sixth recycle, only multi-phosphorylated peptides (*m*/*z* 1562, 2966, and 3122) were captured and eluted (Figure S6c). Furthermore, the intensities of the quarterly phosphorylated peptides (*m*/*z* 3122, 2966) are much stronger than those of the doubly phosphorylated peptides. This would be reasonable considering that the multi-phosphorylated peptides have more sites and stronger coordinating ability to Ti^4+^ ions in the 3D-network polymer brushes. If they were not completely eluted out after several recycles, multi-phosphopeptides would occupy all coordination sites, leaving no chance for mono-phosphopeptides. Two batches of G@mSiO_2_@PPFIL-Ti^4+^ were randomly selected for reproducibility evaluation using the tryptic digest of β-casein (2 pmol). As shown in Figure S7, there is no noticeable difference between two-batch adsorbents regarding enrichment capacity, verifying their excellent reproducibility.

### 3.3. Application of the G@mSiO_2_@PPFIL-Ti^4+^ Nanocomposite in Phosphopeptide Enrichment from Human Serum, Saliva, and Lung Cells

Human serum was used as a typical model sample to assay the capability of the G@mSiO_2_@PPFIL-Ti^4+^ composite to capture endogenous phosphopeptides from complex biosamples. As shown in Figure S8, four characteristic phosphopeptides were observed with clean backgrounds, and the assignment of the phosphopeptides is listed in Table S3. Then, we evaluated the enrichment specificity from the easily accessible biological sample of human saliva, which is assumed to contain potential biomarkers [52,53]. In these assays, two procedures were deliberately applied: (1) direct elution with 10% NH_4_OH to collect global phosphopeptides and (2) the two-step elution process, 0.6% NH_4_OH followed by 10% NH_4_OH, to determine whether mono- and multi-phosphopeptides could be completely isolated. When directly eluted with 10% NH_4_OH, 25 phosphopeptides were captured globally, of which 8 were mono-phosphopeptides (Figure S9a). In the two-step process, 14 phosphorylated peptides were released during the first elution using 0.6% NH_4_OH, of which mono-phosphopeptides dominated the spectrum (mono-/multi-phosphopeptides, 9/5), as shown in Figure S9b. The ratio of mono-/multi-phosphopeptides was markedly increased. The following elution step with 10% NH_4_OH gave 19 phosphopeptides, of which the desired multi-phosphopeptides predominately appeared in the spectrum (mono-/multi-phosphopeptides, 3/16) (Figure S9c), exhibiting significant enhancement in specifically isolating multi-phosphopeptides. The assignment of all endogenous phosphorylated peptides captured in human saliva is tabulated in Table S4. The quantity of endogenous phosphopeptides enriched by the adsorbent materials developed in this study surpasses that achieved by previously reported IMAC enrichment materials, such as readily separable Fe_3_O_4_ magnetic sphere substrates and hydrophilic chelating ligands (Table S2). Furthermore, compared to the number of global phosphopeptides obtained by the one-step strategy, five more phosphorylated peptides were isolated by the two-step strategy. These findings reveal that the G@mSiO_2_@PPFIL-Ti^4+^ composite has potential in the selective isolation of mono- and multi-phosphopeptides from real biological samples.

**Figure 4:**
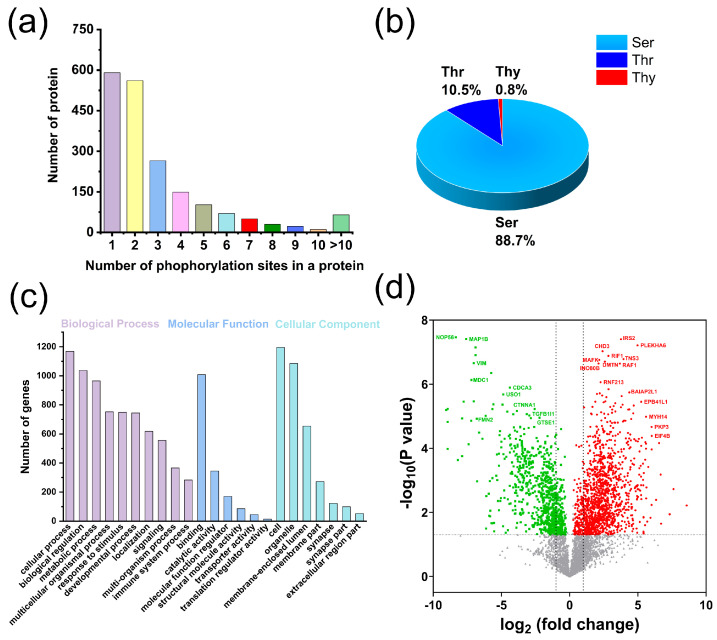
(**a**) Identification and characterization of phosphoproteins in the human lung cells enriched by using G@mSiO_2_@PPFIL-Ti^4+^ nanocomposite. (**b**) The distribution of the number of phosphorylation sites at various amino acid residues. (**c**) GO enrichment analysis of identified phosphoproteins across three categories: biological process, cellular component, and molecular function. (**d**) The volcano plot illustrates the differentially expressed phosphoproteins between SPC-A1 cells and Beas-2B cells.

There is a growing trend in cancer research of the paradigm evolving into the multi-omics strategy by integrating all data of multiple events rather than relying on a single event [54]. Phosphoproteomics emerges as a useful tool in cancer studies; however, the quality of phosphoproteomics analysis is heavily dependent on the enrichment performance of the adsorbent materials in terms of phosphopeptide specificity, selectivity, and wider coverage. It is widely recognized that lung cancer is the leading cause of cancer-related deaths, with approximately 85% of lung cancer cases classified as NSCLC. Among the three main subtypes of NSCLC, adenocarcinoma is the most prevalent, accounting for about 40% of cases [55]. The main drawbacks of traditional computed tomography (CT) detection technology, including a high risk of radiation overexposure and a high false-positive rate, compromise the reliability of early detection [56]. Therefore, we further attempted to explore the feasibility of using the G@mSiO_2_@PPFIL-Ti^4+^ composite in an alternative phosphoproteomics approach. Normal human lung epithelial cells (Beas-2B) and human lung adenocarcinoma cells (SPC-A1) were used in phosphopeptide enrichment, the phosphorylated peptides were identified by LC-MS/MS, and label-free quantification was used in the phosphoproteomic analysis. The phosphopeptides we have enriched are all at localization probabilities greater than 0.5. For biological triplicate normal human lung epithelial cell samples, 1271, 1009, and 1141 phosphopeptides were identified, respectively, as shown in Figure 3d. The information on sequence and phosphorylation sites is listed in Table S5. Quantitative analysis revealed 1155, 1429, and 1294 non-phosphopeptides across biological repetition, with enrichment specificity ranging from 47% to 53%. In total, there were 1145 unique phosphopeptides found from normal human lung epithelial cell (Beas-2B) lysates, and 856 phosphopeptides overlapped within the biological triplicate (Figure 3e). More information can be obtained in comparison with the data from human lung adenocarcinoma cell (SPC-A1) lysates. In total, 1418, 1727, and 1711 phosphopeptides were identified, respectively (Figure 3d and Table S5). In cancer cell lines, the number of enriched non-phosphopeptides was notably reduced to 1047, 532, and 559, accompanied by a significant improvement in enrichment specificity (60–75%). There are 1456 unique phosphopeptides found in human lung adenocarcinoma cell lysates, representing a 27% increase over the Beas-2B samples (Figure 3f). These findings unequivocally demonstrate the material’s superior performance in selectively enriching phosphopeptides, particularly within lung cancer cell models. These data provide a robust foundation for downstream analyses of cancer-associated signaling pathways and biomarker discovery, offering high-confidence phosphoproteomic datasets for interrogating disease-relevant molecular mechanisms. In addition, mono-, di-, and tri-phosphorylated proteins constitute 74% of all identified phosphoproteins, and 88.7%, 10.5%, and 0.8% of the phosphorylation sites were found in the amino acid residues of serine, threonine, and tyrosine, respectively (Figure 4a,b). This coverage is comparable to many previous studies, indicating that our adsorbent has unbiased enrichment capability for phosphorylated peptides [57].

To validate the identified phosphorylation sites, we employed WebLogo software (https://weblogo.berkeley.edu/logo.cgi) to conduct a sequence pattern analysis, the results of which are detailed in the Supplementary Materials of this manuscript (Figure S10). The generated sequence logo clearly illustrates that serine residues (S) undergoing phosphorylation are flanked by a high prevalence of alanine (A), aspartic acid (D), and leucine (L). Intriguingly, this specific amino acid composition aligns with the known substrate preference profile of kinases, particularly those recognizing acidic or hydrophobic motifs. This observed sequence pattern is consistent with the established principles governing kinase–substrate interactions, thereby providing additional biochemical evidence that strengthens the reliability of our phosphorylation site assignments [58].

These results clearly verify that the G@mSiO_2_@PPFIL-Ti^4+^ composite maintains excellent phosphopeptide specificity with wide coverage in the treatment of real, complicated samples. However, at present, our exploration of biological samples is stalled at examining the enrichment properties of the materials, while the types of samples have not been explored or selected regarding more aspects, such as the subtypes of lung cancer. We have cited studies showing the effects of specific phosphoprotein alteration bands in different lung cancer subtypes (adenocarcinoma and squamous cell carcinoma) and discussed both our findings and the published literature. As described in the study, the mutant EGFR protein exhibits persistent hyperphosphorylation, thereby contributing to the progression of lung adenocarcinoma [59]. Another study revealed that PTEN loss in stage IV squamous cell lung carcinoma promotes tumor progression through coordinated hyperphosphorylation of AKT and FAK, driving increased proliferation, invasiveness, and the epithelial–mesenchymal transition of lung cancer cells [60]. These studies are consistent with the hyperphosphorylation of AKT and EGFR in our study, strengthening the clinical relevance of our work. In our future investigations, we aim to further elucidate the mechanistic relationship between the phosphorylation processes in diverse biological specimens and the oncogenic progression of carcinogenesis.

To explore the potential functions of phosphorylated proteins in NSCLC, we conducted GO enrichment analyses (Figure 4c). The biological process analysis revealed that the majority of differentially expressed phosphoproteins were primarily enriched in cellular processes, biological regulation, responses to stimuli, metabolic processes, developmental processes, etc. In the molecular function analysis, the identified phosphoproteins were predominantly involved in binding, catalytic activity, molecular function regulation, structural molecular activity, and transporter activity. The cellular component analysis indicated that most of the identified phosphoproteins are localized within membrane-bounded organelles [58,61].

The volcano plot illustrates the differentially expressed phosphoproteins in lung cancer cells of SPC-A1 compared to normal lung cells of Beas-2B (Figure 4d). Among the 2698 differentially expressed phosphopeptides (*p* < 0.05), 1592 were upregulated and 1106 were downregulated. Key proteins showing significant upregulation include insulin receptor substrate 2 (IRS2), chromodomain helicase DNA binding protein 3 (CHD3), pleckstrin homology domain-containing A6 (PLEKHA6), replication timing regulatory factor 1 (RIF1), MAF bZIP transcription factor K (MAFK), dematin (DMTN), raf-1 proto-oncogene, serine/threonine kinase (RAF1), and brain-specific angiogenesis inhibitor 1-associated protein 2-like protein 1 (BAIAP2L1). RIF1, in particular, was found to be highly expressed in NSCLC at both mRNA and protein levels. Its expression was significantly correlated with clinical stages (*p* < 0.05) and patients’ prognosis (*p* < 0.001). Functional studies demonstrated that RIF1 knockdown inhibited NSCLC cell growth both in vitro and in vivo. Conversely, RIF1 overexpression promoted NSCLC cell growth, cell cycle progression, and cancer stem cell properties. This effect was driven by the enhanced protein phosphatase 1–axin (PP1-AXIN) interactions, which subsequently triggered the activation of the Wnt/β-catenin signaling pathway. The Wnt/β-catenin pathway plays a vital role in tumor development. The expression levels and phosphorylation status of proteins within this pathway directly affect its activation, thereby influencing tumor occurrence and progression. RIF1 is a novel oncogenic regulator of Wnt/β-catenin signaling. RIF1 is highly expressed in NSCLC and is significantly associated with clinical stage and poor prognosis. It promotes NSCLC cell growth, cell cycle progression, and cancer stem cell (CSC)-like properties by enhancing the PP1–AXIN interaction, leading to AXIN dephosphorylation and the subsequent activation of Wnt/β-catenin signaling. Clinically, RIF1 protein levels correlate positively with β-catenin in NSCLC tissues. Therefore, RIF1 contributes to the NSCLC progression [62]. DMTN functions as a tumor suppressor and plays a critical role in inhibiting malignant cell transformation. Overexpression of DMTN has been shown to suppress the proliferation and invasion of glioblastoma multiforme by regulating the cell cycle and actin cytoskeleton remodeling, respectively [63]. IRS2 (insulin receptor substrate 2) is a key mediator of insulin signaling and regulates various cellular processes [64]. It was reported that the IRS2-PI3K (phosphatidylinositol 3-kinase) signaling pathway promoted tumorigenesis by enhancing MYC (myelocytomatosis oncogene protein) expression [65]. On the other hand, significant downregulation was particularly evident in several key proteins, including nucleolar protein 56 (NOP56), microtubule-associated protein 1B (MAP1B), and vimentin (VIM). Cancer cells frequently exhibit high levels of vimentin expression, which enhances their invasive potential and contributes to cancer metastasis [66]. The phosphorylation of vimentin plays a critical role in regulating its structure and function. Specifically, the phosphorylation of vimentin at serine 56 has been shown to suppress stemness properties, tumor initiation, and metastasis [67]. Differential protein analysis has demonstrated that the material used in this study exhibits exceptional performance in phosphopeptide enrichment when comparing Beas-2B and SPC-A-1 cells. Additionally, phosphoproteomic analysis of human lung cancer cells shows promise for identifying novel tumor biomarkers.

**Figure 5:**
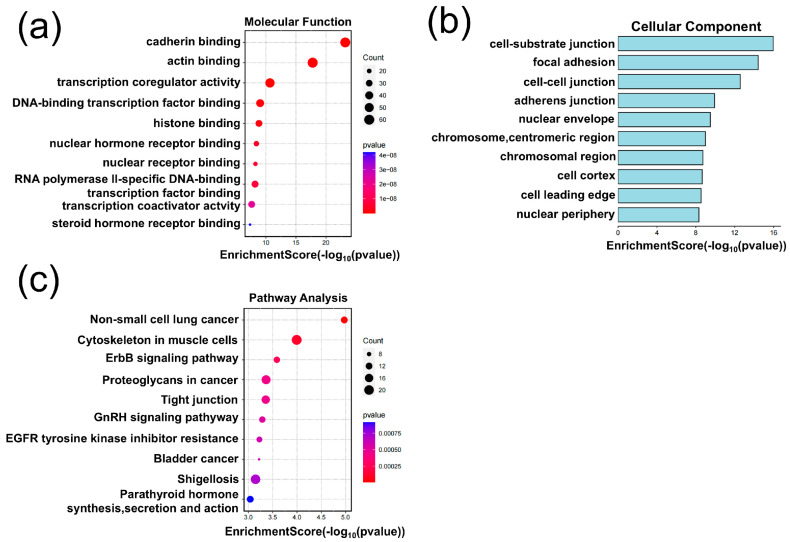
GO and KEGG pathway enrichment analysis of differentially expressed genes in Beas-2B and SPC-A1 cells. (**a**) Molecular function analysis; (**b**) Cell component analysis; (**c**) KEGG pathway analysis.

Subsequently, we collected the genes associated with the identified phosphorylated peptides. Differential gene expression analysis between Beas-2B and SPC-A1 cells was performed with a significance threshold of *p* < 0.05 and a log_2_ fold change > 2. A total of 706 differentially expressed genes were identified, with 508 genes upregulated and 198 genes downregulated in SPC-A1 cells compared to Beas-2B cells. These 706 differentially expressed genes were subsequently subjected to Gene Ontology (GO) and Kyoto Encyclopedia of Genes and Genomes (KEGG) pathway analyses (Figure 5). Biological process analysis demonstrated that the differentially expressed genes were predominantly associated with actin filament organization, the regulation of actin filament-based processes, and the regulation of actin filament organization (Figure S11). These findings collectively indicate that the identified genes play a crucial role in the regulation of actin filament-related processes. Given that cancer progression is closely linked to alterations in the structure and behavior of cancer cells, these changes might be largely driven by the remodeling of the cytoskeleton, particularly the actin structure. Molecular function analysis (Figure 5a) further revealed that the differentially expressed genes were implicated in actin binding, which aligns with their involvement in the regulation of actin filament organization as identified in the biological process analysis (Figure S11). Additionally, cell component analysis (Figure 5b) indicated that these genes were associated with cell–cell junctions, which are critical for communication between tumor cells and stromal cells. Notably, the epithelial–mesenchymal transition (EMT), a pivotal step in cancer metastasis, is influenced by cell–cell junctions, which may facilitate cell adhesion and migration in cancers. KEGG pathway analysis (Figure 5c) highlighted that the differentially expressed genes were enriched in pathways related to NSCLC and bladder cancer, as well as the ErbB signaling pathway. The ErbB family regulates cell proliferation and differentiation by binding growth factors and activating the phosphatidylinositol-3-kinase (PI-3K) pathway, a key driver of cancer development, either directly or indirectly [68]. These analyses highlight the critical roles of actin filament regulation, cell–cell junctions, and ErbB signaling in cancer progression, offering valuable insights into the molecular mechanisms driving tumorigenesis and metastasis. Although our investigation was preliminary, the G@mSiO_2_@PPFIL-Ti^4+^ composite exhibited promising performance and potential for phosphopeptide enrichment and the identification of lung cancer-associated biomarkers. Further exploration using additional methodologies will be conducted to validate and expand upon these findings.

## 4. Conclusions

A novel IMAC composite has been successfully orchestrated and functionalized by poly(ionic liquid) polymeric brushes. The constructed 3D network is characterized by its exceptional robustness combined with remarkable flexibility, high tolerance to acidity and basicity, and high content of metal ions, thus endowing the G@mSiO_2_@PPFIL-Ti^4+^ adsorbent with excellent enrichment performance. Furthermore, phosphorylated peptides can be enriched and eluted out globally, or respective mono- and multi-phosphopeptides can be differentially isolated. The application of this material in the phosphorylation studies of lung cells may suggest that the G@mSiO_2_@PPFIL-Ti^4+^ adsorbent could serve as a multi-purpose platform for phosphoproteomics research, biomedical diagnostics, and pharmaceutical development.

## Data Availability

The original contributions presented in this study are included in the article/Supplementary Material. Further inquiries can be directed to the corresponding authors.

## Supplementary Materials

The following supporting information can be downloaded at: https://www.mdpi.com/article/10.3390/biom15060894/s1, Figure S1: N_2_ adsorption−desorption isotherm of G@mSiO_2_; Figure S2: SEM image and EDX analysis of G@mSiO_2_; Figure S3: EDX analysis for G@mSiO_2_@PPFILOEt (a) and G@mSiO_2_@PPFIL-Ti^4+^ (b); water contact angle of G@mSiO_2_@PPFILOEt (c) and G@mSiO_2_@PPFIL-Ti^4+^; Figure S4: The MALDI-TOF MS spectra of tryptic digest mixture of β-casein (200 fmol) with different loading buffers containing 50% ACN and (a) 0.5% TFA, (b) 1% TFA, (c) 3% TFA, and (d) 6% TFA. Elution buffer, NH_4_OH solution (3 v%). Different elution buffers of NH_4_OH concentrations diluted with deionized water (e) 5 v%, (f) 10 v%. Loading buffers, 50% ACN and 6% TFA. The peaks of phosphopeptides are marked with *, and the peaks of dephosphopeptides are marked with ●; Figure S5: MALDI-TOF MS for the mixtures (a) β-casein digest and BSA protein (mass ratio 1/1000) and (b) β-casein digest, β-casein protein, and BSA protein (mass ratio 1/1000/1000) enriched by G@mSiO_2_@PPFIL-Ti^4+^; Figure S6: MALDI-TOF MS spectra of β-casein trypsin digestion of G@mSiO_2_@PPFIL-Ti^4+^ nanocomposites after (a) 1, (b) 3, and (c) 6 enrichments. The peaks of phosphopeptides are marked with *, and the peaks of dephosphopeptides are marked with ●. The phosphopeptides are marked with *, and the dephosphorylated peptides are marked with ●; Figure S7: MALDI-TOF MS spectra of β-casein tryptic digest after enrichment by different batches G@mSiO_2_@PPFIL-Ti^4+^ nanocomposite (a) batch I and (b) batch II. The peaks of phosphopeptides are marked with *, and the peaks of dephosphopeptides are marked with ●; Figure S8: MALDI-TOF MS spectra of human serum after enrichment by G@mSiO_2_@PPFIL-Ti^4+^; Figure S9: MALDI-TOF MS spectra of human saliva after enrichment by G@mSiO_2_@PPFIL-Ti^4+^. (a) directly eluted by 10% NH_4_OH and gradient elution (b) first eluted by 0.6% NH_4_OH, (c) second eluted by 10% NH_4_OH. The multi- and mono-phosphopeptides were marked red and black, respectively; Figure S10: sequence logo plot of serine phosphorylation sites; Figure S11: The GO analysis of the biological process based on the identified phosphopeptides of normal and lung cancer cells; Table S1: The detailed information of phosphopeptides from β-casein digests enriched by G@mSiO_2_@PPFIL-Ti^4+^; Table S2: Comparison of enrichment performance of our material G@mSiO_2_@PPFIL-Ti^4+^ to the recently reported adsorbent materials analyzed by MALDI-TOF MS; Table S3: List of phosphopeptides enriched from human serum; Table S4: Detailed information of phosphopeptides identified from human saliva; Table S5: The phosphopeptides obtained from the tryptic digest of human lung cells lysate enriched by G@mSiO_2_@PPFIL-Ti^4+^ nanocomposite.
