# Supplementary material for: Dynamic 3D-Network Coating Composite Enables Global Isolation of Phosphopeptides, Stepwise Separation of Mono- and Multi-Phosphopeptides, and Phosphoproteomics of Human Lung Cells
Source: Biomolecules. 2025 Jun 18;15(6):894. doi: 10.3390/biom15060894 (PMC12190523; doi:10.3390/biom15060894)
Supplement: Supplementary file 1 [file biomolecules-15-00894-s001.zip › biomolecules-3620704-supplementary.pdf]

biomolecules

MDPI

*Supplementary Materials*

# Dynamic 3D-Network Coating Composite Enables Global Isolation of Phosphopeptides, Stepwise Separation of Mono- and Multi-Phosphopeptides, and Phosphoproteomics of Human Lung Cells

**Linlin Liu, Zhenhua Chen, Danni Wang, Weida Liang, Binbin Wang, Chenglong Xia, Yinghua Yan, Chuanfan Ding, Xiaodan Meng and Hongze Liang**

**Contents**

**Chemicals and Materials**

**Characterization and Measurement**

**Sample preparation**

**MALDI-TOF MS Analysis**

**LC-MS/MS Analysis and Data Search**

**Figures S1–S10**

**Tables S1–S5**

**References**

## Chemicals and Materials

Graphene was obtained from Nanjing XFNANO Materials Tech Co., Ltd. (Nanjing, China). Tetraethyl orthosilicate (TEOS), sodium hydroxide, titanium sulfate ($Ti(SO_4)_2$), acetonitrile (ACN) and ammonia

 https://doi.org/10.3390/biom15060894

solution (25-28 wt%) were purchased from Sinopharm Chemical Reagent Co., Ltd. (Shanghai, China). Cetyltrimethylammonium ammonium bromide (CTAB) was obtained from Beijing Solarbio Science & Technology (Beijing, China). Vinylimidazole was obtained from Rhawn (Shanghai, China). Triethoxyvinylsilane was obtained from Adamas beta (Shanghai, China). Triethylamine was obtained from Shanghai Linger Chemical Co., Ltd. (Shanghai, China). Trichloromethane was obtained from Wuxi Jiani Chemical Co., Ltd. (Wuxi, China). 1, 3-dibromopropane was obtained from HWRK Chem (Beijing, China). Triethyl phosphite was purchased from Chengdu Gracia Chemical Technology (Chengdu, China). Hydrobromic acid (48 wt%) was obtained from J&K Chemical (Shanghai, China). Pyridoxal 5'-phosphate was obtained from Shanghai Yuanye Biotechnology (Shanghai, China). Trifluoroacetic acid was purchased from Aladdin (Shanghai, China). β-casein, bovine serum albumin (BSA), iodoacetamide (IAA), dithiothreitol (DTT) and 2,5-dihydroxybenzoic (DHB) were all obtained from Sigma Aldrich (Shanghai, China). Collections of human serum and saliva samples from a healthy volunteer were conducted according to the protocol approved by Ningbo University Ethical Board. The cell lines utilized in this study were purchased from the Cell Bank/Stem Cell Bank (Shanghai, China) and included Beas-2B and SPC-A1-1. We used DMEM basal medium (Corning, New York, USA) to culture the human normal epithelial cells Beas-2B, while the SPC-A1-1 lung cancer cell was cultured in RPMl-1640 basal medium (Corning, New York, USA). To the cells, we added 10% fetal bovine serum (FBS; PAN Biotech, Aidenbach, Germany), 100 μg/mL penicillin, and 100 U/mL streptomycin (Beijing Solarbio Science & Technology Co., Ltd., China). All the cells were cultured at 37 °C in a humidified incubator with 5% $CO_2$.

## Characterization and Measurement

Scanning electron microscopy (SEM) images and energy dispersive X-ray analysis (EDX) were recorded on a Nova NanoSEM 450 electron microscope (Thermo Fisher Scientific Inc., Waltham, Massachusetts, USA). Fourier-transform infrared spectroscopy (FT-IR) was taken on a NICOLET 6700 Fourier transform

infrared spectrometer (Thermo Fisher Scientific Inc., Waltham, Massachusetts, USA) using KBr pellets. Inductively coupled plasma optical emission spectroscopy (ICP-OES) was analyzed on an Aglient 5110 (Aglient, Santa Clara, California, USA). The zeta potentials were measured on a Zetasizer Nano ZS90 analyzer (Malvern Panalytical Ltd., Malvern, Worcestershire, UK). Thermogravimetric analysis (TG) was obtained on the Q500 Thermogravimetric analyzer (TA Instruments, Inc., New Castle, USA). Nitrogen adsorption-desorption isotherms were measured on an ASAP2020 analyzer (Micromeritics Instrument Corporation, Norcross, USA). The Brunauer-Emmet-Teller (BET) method was employed to calculate the specific surface areas. Water contact angles were measured on a Dataphysics OCA25 (DataPhysics Instruments GmbH, Ostfildern, Germany) with 3.0 μL water drop. UV-Vis spectra were recorded on a UV-2600 spectrophotometer (Shimadzu Corporation, Japan). MALDI-TOF MS experiments were conducted on a 5800 Proteomics Analyzer (Applied Biosystems, California, USA) with a Nd-YAG laser at 355 nm, and the measurement was at an acceleration voltage of 20 KV in the positive ion mode.

**Sample Preparation.**

The solutions of digested β-casein (5 mg) and BSA (10 mg) were prepared according to our previous work. For the study on selectivity, a series of mixed samples were prepared as follows. Each digest of β-casein (kept at 1.43 pmol) was diluted by varying the amount of digested BSA to 1:1000, 1:5000, 1:10000, and 1:15000 (molar ratio), respectively. For the study on size-exclusion effect, each digest of β-casein (kept at 2 pmol) was diluted by different amount BSA to 1:1000 (mass ratio) and addition of β-casein and BSA to reach mass ratio 1:1000:1000.

**MALDI-TOF MS Analysis.**

0.5 μL of eluent and 0.5 μL of DHB solution (20 mg $mL^{-1}$, 50% ACN/ 49% $H_2O$/ 1% $H_3PO_4$, v/v/v) was mixed completely. The mixed solution was dropped upon the plate and dried, and analyzed by MALDI-TOF

MS. MALDI-TOF spectrometer autoflex maX (Bruker Scientific, USA) was used in positive ion mode with an Nd: YAG laser (383 nm). The acceleration voltage was run at 20 kV, and the repetition rate was 1000 Hz.

**LC-MS/MS Analysis and Data Search.**

Phosphopeptides of human lung cell lysate were analyzed on a nanoElute system (Bruker Daltonics) coupled with timsTOF HT (Bruker Daltonics) with nanoelectrospray ion source. Lyophilized sample was redissolved in 1% TFA (20 μL), loaded on a trap column (75 μm×40 cm, 2.5 μm, 200 Å, C18, Bruker/PepSep), and eluted and separated through an analytical column (75 μm×15 cm, 1.9 μm, 200 Å, C18, Bruker/PepSep) with mobile phase A (deionized $H_2O$ with 0.1% formic acid) and mobile phase B (100% acetonitrile with 0.1% formic acid) for 60 min. Elution gradient was set as follows: 0–50 min, 5–30% B; 50–55 min, 30–95% B; 55–60 min, 95% B. The flow rate was 3 L/min. The mass spectrometer was run in dia-PASEF mode. Full-scan MS spectra (m/z 100–1700) were acquired in Orbitrap with a resolution of 120,000 at m/z 200. MS2 spectra were obtained with a resolution of 20,000 at m/z 200.

Raw data were analyzed with MSFragger in FragPipe (v21.1). The database was uniport knowledge base (downloaded on April 15,2024,20598 protein sequences). Search parameters were set as follows: Variable modifications, oxidation (M), acetyl (N-term), phospho (STY); Fixed modification, carbamidomethylation (C); Digestion mode, trypsin; Maximum missed cleavages, 2; Main search ppm, 6; PSM FDR, 0.01; Protein FDR, 0.01; Min peptide length, 2; FTMS MS/MS tolerance, 20 ppm; FTMS top peaks per 100 Da, 12.

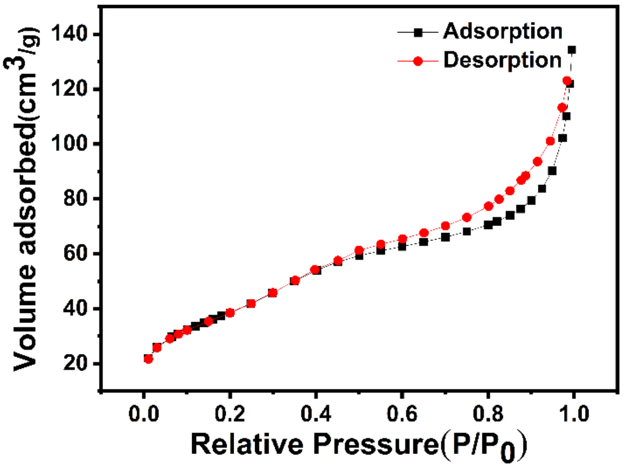


**Figure S1**. $N_2$ adsorption−desorption isotherm of G@mSiO$_2$

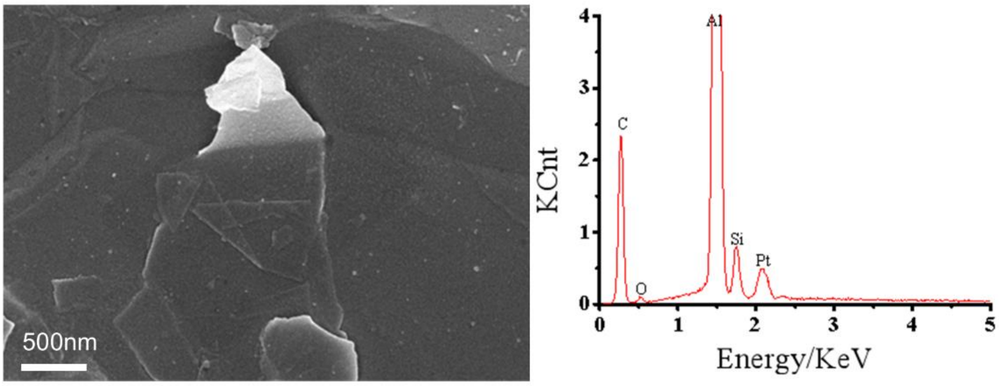


**Figure S2.** SEM image and EDX analysis of G@mSiO$_2$

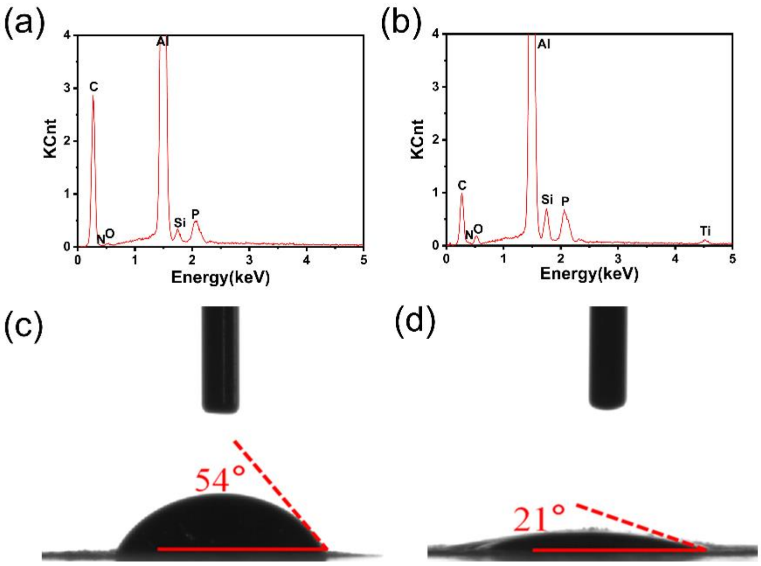


**Figure 3.** EDX analysis for G@mSiO$_2$@PPFILOEt (a) and G@mSiO$_2$@PPFIL-$Ti^{4+}$ (b); water contact angle of G@mSiO$_2$@PPFILOEt (c) and G@mSiO$_2$@PPFIL-$Ti^{4+}$ (d).

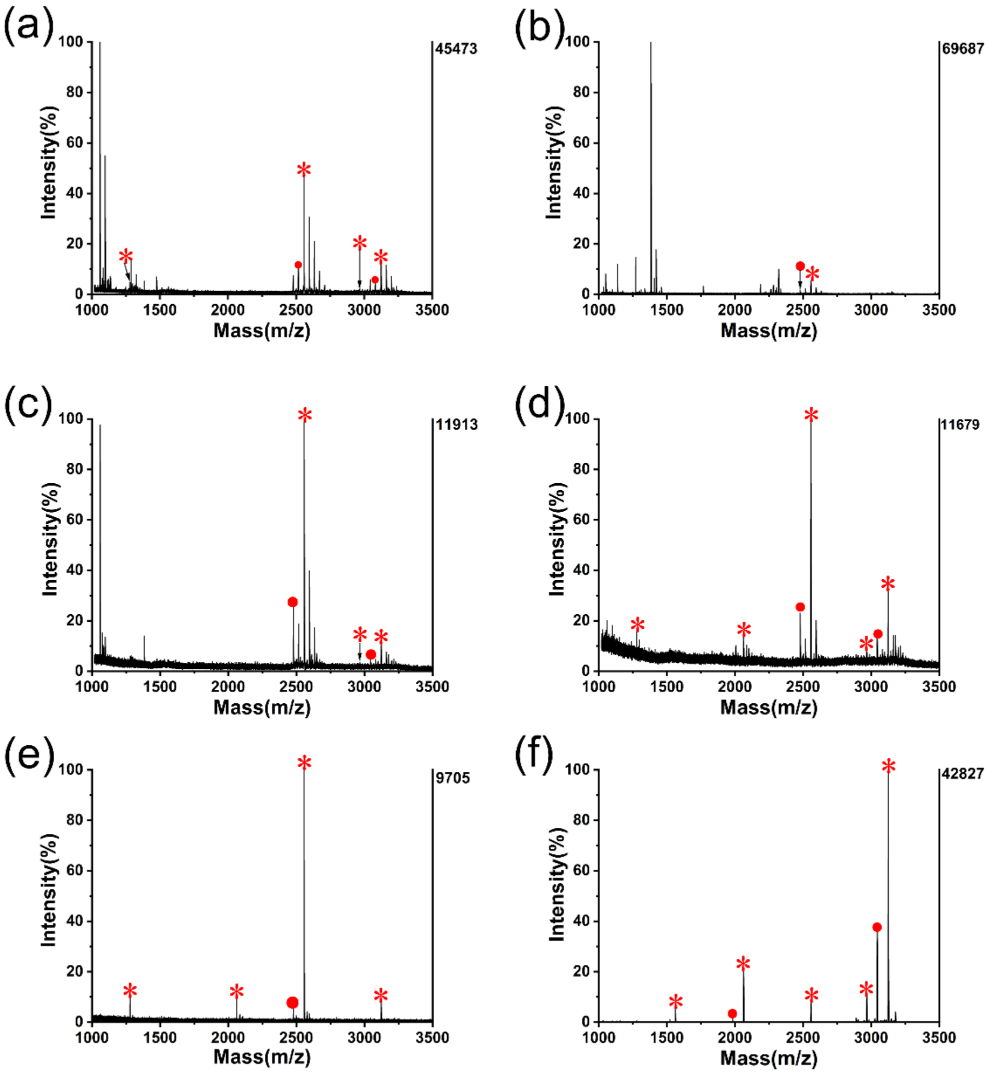


**Figure S4.** The MALDI-TOF MS spectra of tryptic digest mixture of β-casein (200 fmol) with different loading buffers containing 50% ACN and (a) 0.5% TFA, (b) 1% TFA, (c) 3% TFA, (d) 6% TFA. Elution buffer, $NH_4OH$ solution (3 v%). Different elution buffers of $NH_4OH$ concentrations diluted with deionized water (e) 5 v%, (f) 10 v%. Loading buffers, 50% ACN and 6% TFA. The peaks of phosphopeptides are marked with *, and the peaks of dephosphopeptides are marked with ●.

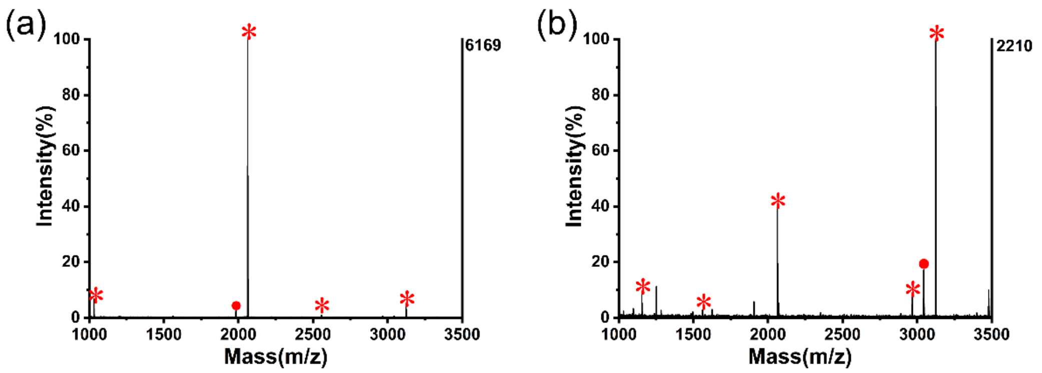


**Figure S5**. MALDI-TOF MS for the mixtures (a) β-casein digest and BSA protein (mass ratio 1/1000) and (b) β-casein digest, β-casein protein and BSA protein (mass ratio 1/1000/1000) enriched by G@mSiO2@PPFIL-$Ti^{4+}$. The phosphopeptides are marked with *, and the dephosphorylated peptides are marked with ●.

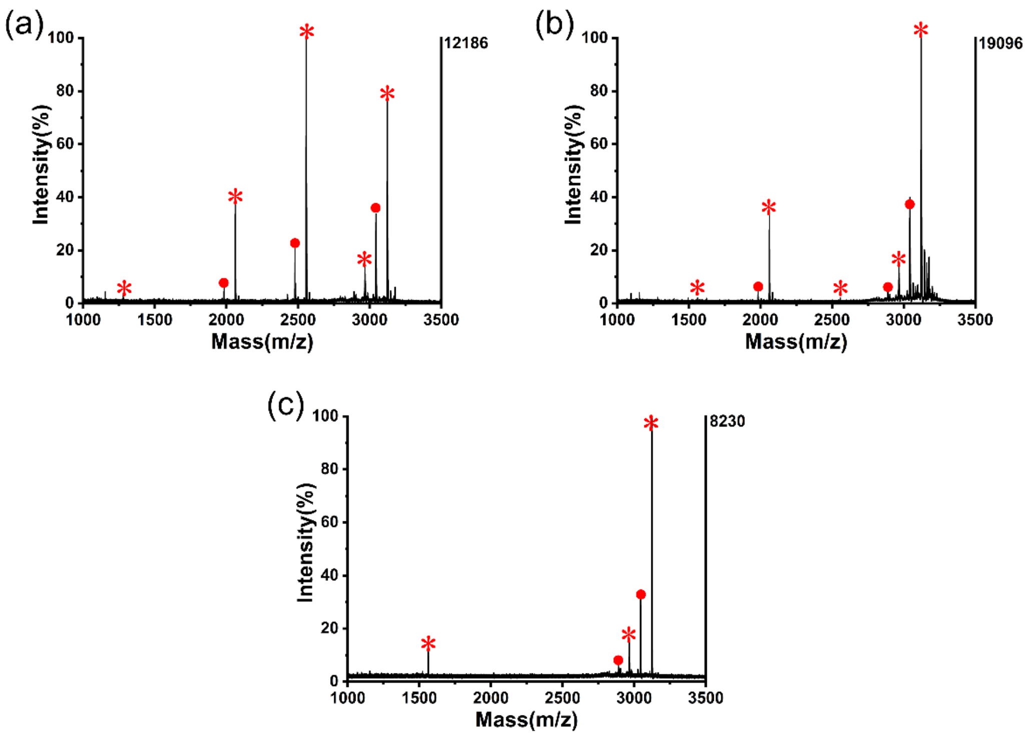


**Figure S6.** MALDI-TOF MS spectra of β-casein trypsin digestion of G@mSiO2@PPFIL-$Ti^{4+}$ nanocomposites after (a) 1, (b) 3, and (c) 6 times enrichment. The peaks of phosphopeptides are marked with *, and the peaks of dephosphopeptides are marked with ●.

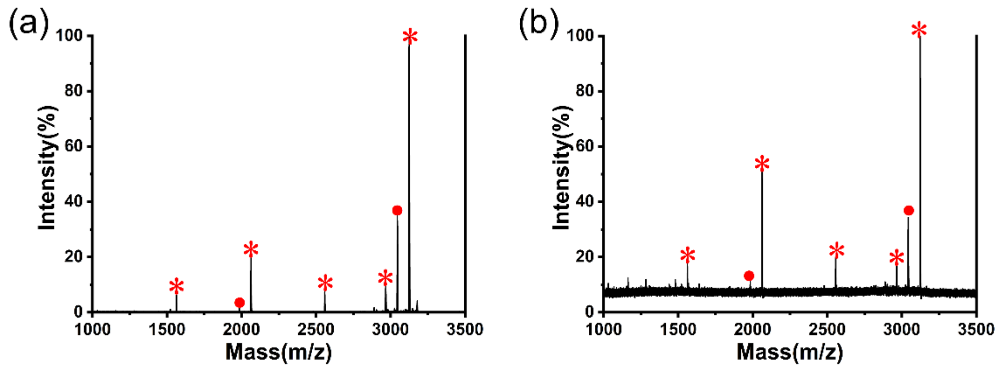


**Figure S7.** MALDI-TOF MS spectra of β-casein tryptic digest after enrichment by different batches G@mSiO2@PPFIL-$Ti^{4+}$ nanocomposite (a) batch I and (b) batch II. The peaks of phosphopeptides are marked with *, and the peaks of dephosphopeptides are marked with ●.

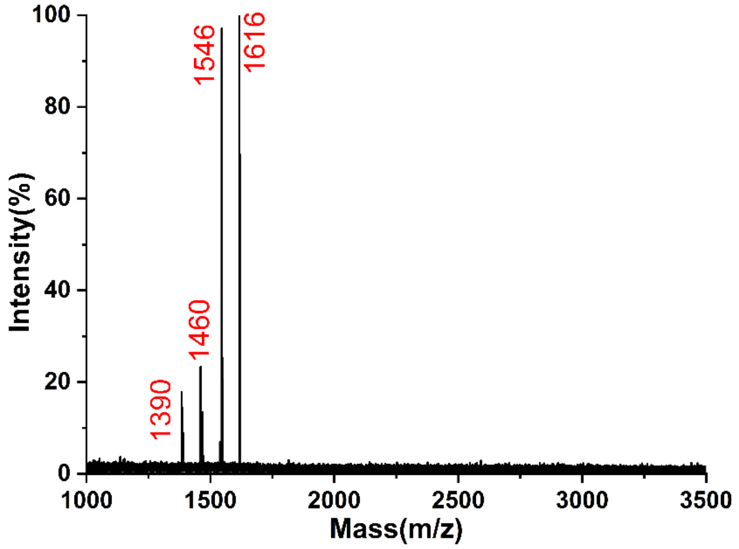


**Figure S8.** MALDI-TOF MS spectra of human serum after enrichment by G@mSiO2@PPFIL-$Ti^{4+}$.

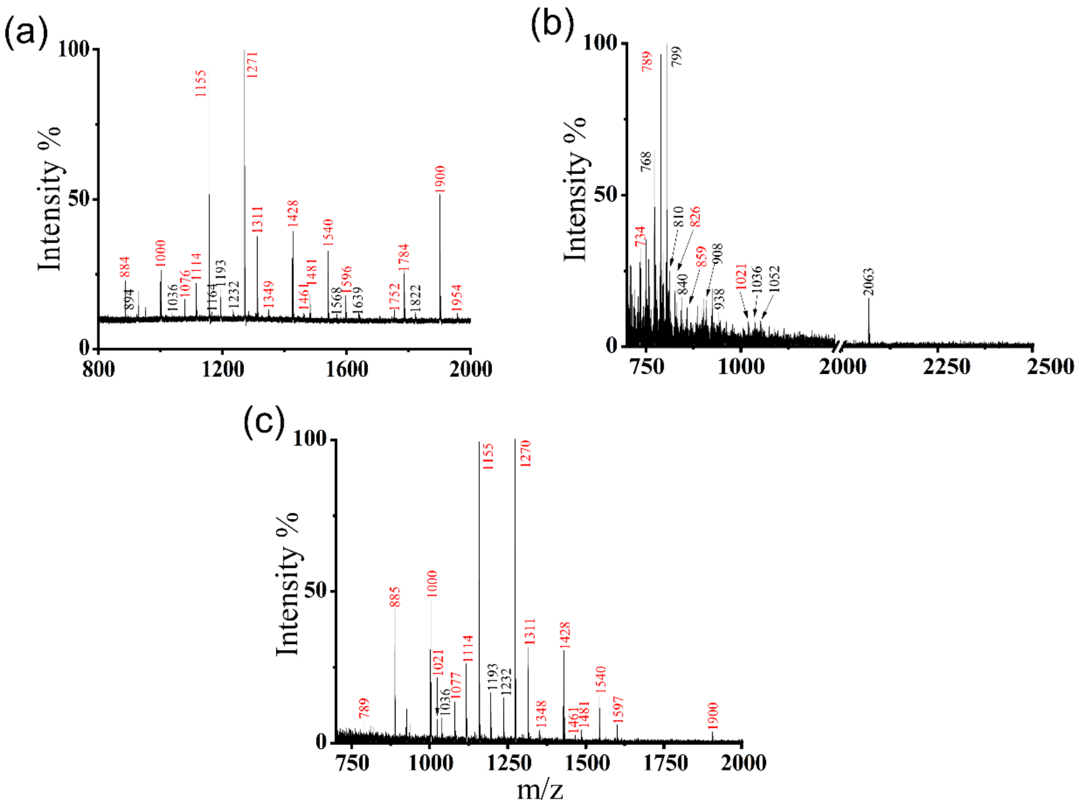


**Figure S9.** MALDI-TOF MS spectra of human saliva after enrichment by G@mSiO$_2$@PPFIL-Ti$^{4+}$. (a) directly eluted by 10% $NH_4OH$ and gradient elution (b) first eluted by 0.6% $NH_4OH$, (c) second eluted by 10% $NH_4OH$. The multi- and mono-phosphopeptides were marked red and black, respectively.

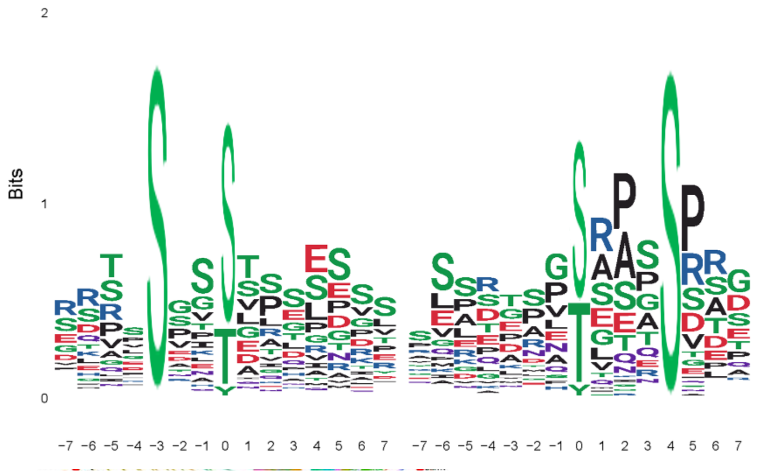


**Figure S10.** sequence logo plot of serine phosphorylation sites.

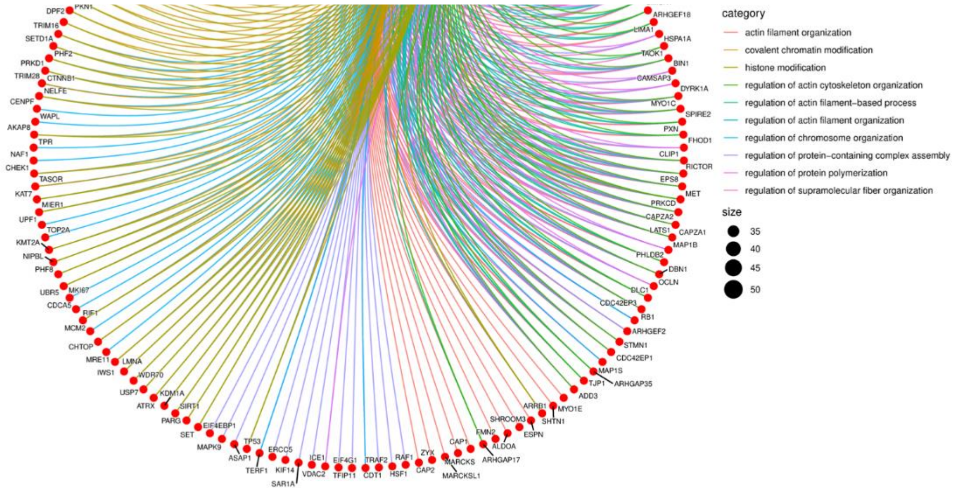


**Figure S11.** The GO analysis of the biological process based on the identified phosphopeptides of normal and lung cancer cells.

**Table S1.** The detailed information of phosphopeptides from β-casein digests enriched by G@mSiO2@PPFIL-$Ti^{4+}$

| No | m/z | Sequence |
|---|---|---|
| 1 | 1031 | FQ[PS]EEQQQTEDELQDK |
| 2 | 1278 | FQ[PS]EEQQQTEDELQDKIHPF |
| 3 | 1562 | GILAADE[PS]TG[PS]IAKR |
| 4 | 2061 | FQ[PS]EEQQQTEDELQDK |
| 5 | 2556 | FQ[PS]EEQQQTEDELQDKIHPF |
| 6 | 2966 | ELEELNVPGEIVE[PS]L[PS][PS][PS]EESITR |
| 7 | 3122 | RELEELNVPGEIVE[PS]L[PS][PS][PS]EESITR |

**Table S2.** Comparison of enrichment performance of our material G@mSiO2@PPFIL-$Ti^{4+}$ to the recently reported adsorbent materials analyzed by MALDI-TOF MS.

| Affinity Materials | Sensitivity (*β*-casein) | Selectivity *β*-casein/BSA (molar ratio) | The Number of Phosphopeptides from Saliva (mono-/multi-) | Ref |
|---|---|---|---|---|
| $Fe_3O_4$@GO@PDA-$Ti^{4+}$ | 2.5 fmol/μL | 1/1000 | - | [1] |
| $Fe_3O_4$@PDA-PEI-$Fe^{3+}$ | 0.4 fmol | 1/500 | 18 | [2] |
| CH-Dha-$Ti^{4+}$ | 0.2 fmol | 1/1000 | - | [3] |
| mNi@N-GrT@PDA@$Ti^{4+}$ | 1 fmol | 1/100 | - | [4] |
| $Fe_3O_4$@TAPTDHTA-$Ti^{4+}$ | 0.05 fmol/μL | 1/5000 | 12 | [5] |
| Cotton-Ti-IMAC | 0.1 fmol/μL | 1/1000 | - | [6] |
| Imi-Pops-Zr | 0.5 fmol | 1/1000 | 20 | [7] |
| $Ti^{4+}$-Brush@MAR | 10 fmol | 1/100 (mass ratio) | - | [8] |
| G@m$SiO_2$@PPFIL-$Ti^{4+}$ | 0.1 fmol | 1:15000 | 30(11/19) | This work |

**Table S3.** List of phosphopeptides enriched from human serum.

| No | m/z | Sequence |
|---|---|---|
| 1 | 1390 | DsGEGDFLAEGGGV |
| 2 | 1460 | ADsGEGDFLAEGGGV |
| 3 | 1546 | DsGEGDFLAEGGGVR |
| 4 | 1616 | ADsGEGDFLAEGGGVR |

**Table S4.** Detailed information of phosphopeptides identified from human saliva.

| No | m/z | Sequence |
|---|---|---|
| 1 | 734 | GsYSIK |
| 2 | 768 | KVsAQR |
| 3 | 789 | SKsLFK |
| 4 | 799 | AVsPFAK |
| 5 | 810 | GAFHPsn |
| 6 | 826 | IsVAAASK |
| 7 | 840 | KLsEQR |
| 8 | 859 | LsFSGLR |
| 9 | 885 | KLsEATR |
| 10 | 908 | AEsVVPAR |
| 11 | 938 | KLsELLR |
| 12 | 1000 | ssEEKFL |
| 13 | 1021 | RFsLFGK |
| 14 | 1036 | LPAKLsISK |
| 15 | 1052 | RVtLELGGK |
| 16 | 1077 | sSEEKFLR |
| 17 | 1114 | tDsDEKFI |
| 18 | 1155 | ssEEKFLR |
| 19 | 1193 | DSsEEKFLR |
| 20 | 1232 | sEQFLDEER |
| 21 | 1270 | DssEEKFLR |
| 22 | 1311 | ssEEKFLRR |
| 23 | 1348 | DsHEKRHHGY |
| 24 | 1428 | DssEEKFLRR |
| 25 | 1461 | DLDDFQsWLSR |
| 26 | 1481 | ssEEKFLRRIG |
| 27 | 1540 | SccLIPQTVsHR |
| 28 | 1597 | DssEEKFLRRIG |

| | | |
|---|---|---|
| 29 | 1900 | ISDGGDsEQFLDEERQ |
| 30 | 2063 | AGPPGPPGPPGsIGHPGARGPPG |

**Table S5.** The phosphopeptides obtained from the tryptic digest of human lung cells lysate enriched by G@mSiO2@PPFIL-$Ti^{4+}$ nanocomposite.

| Number | Protein IDs | phospho(STY)probabilities |
|---|---|---|
| 1 | Q9Y6Y0 | NsPQSsPTSTPK |
| 2 | Q9Y6Y0 | NsPQSsPTSTPK |
| 3 | Q9Y6X9 | sPPLPAVIR |
| 4 | Q9Y6X9 | KRsVAVsDEEEVEEEAER |
| 5 | Q9Y6X9 | KRsVAVsDEEEVEEEAER |
| 6 | Q9Y6X9 | DSNELsDsAGEEDSADLK |
| 7 | Q9Y6X9 | DSNELsDsAGEEDSADLK |
| 8 | Q9Y6X9 | KTEsPIKLsPATPSR |
| 9 | Q9Y6M7 | sFADIGKK |
| 10 | Q9Y6M1 | ISYIPDEEVSSPsPPQR |
| 11 | Q9Y6M1 | ISYIPDEEVSsPSPPQR |
| 12 | Q9Y6G9 | DFQEYVEPGEDFPAsPQR |
| 13 | Q9Y6G9 | KPVTVSPTTPTsPTEGEAS |
| 14 | Q9Y6G9 | KPVTVsPTTPTSPTEGEAS |
| 15 | Q9Y6G9 | KPVTVSPTTPtSPTEGEAS |
| 16 | Q9Y6G9 | KPVTVSPTtPTsPTEGEAS |
| 17 | Q9Y6E2 | FVEWLQNAEEEsESEGEEN |
| 18 | Q9Y6E2 | FVEWLQNAEEESEsEGEEN |
| 19 | Q9Y6D5 | ELEKPIQSKPQsPVIQAAAVsPK |
| 20 | Q9Y6D5 | ELEKPIQSKPQsPVIQAAAVsPK |
| 21 | Q9Y6D5 | GQSQLsNPTDDSWK |
| 22 | Q9Y666 | TEAPGtPEGPEPERPSPGDGNPR |
| 23 | Q9Y666 | TEAPGTPEGPEPERPsPGDGNPR |
| 24 | Q9Y666 | DTsLSGFK |
| 25 | Q9Y618 | ANAsPQKPLDLK |
| 26 | Q9Y618 | sPGNTSQPPAFFSK |
| 27 | Q9Y618 | LEPVsPPsPPHTDPELELVPPR |
| 28 | Q9Y618 | LEPVsPPsPPHTDPELELVPPR |
| 29 | Q9Y618 | SLGYHGSSYsPEGVEPVsPVSSPSLTHDK |
| 30 | Q9Y618 | SLGYHGSSYsPEGVEPVsPVSSPSLTHDK |
| 31 | Q9Y613 | EPLIPAsPK |
| 32 | Q9Y613 | sLEGGGCPAR |

| | | |
|---|---|---|
| 33 | Q9Y608 | NSASATTPLsGNSSR |
| 34 | Q9Y606 | VPSPLEGSEGDGDtD |
| 35 | Q9Y606 | VPSPLEGsEGDGDtD |
| 36 | Q9Y5X2 | LSCLFAGPHSTLtPPCsPPEDGLCPH |
| 37 | Q9Y5S9 | GFGsEEGSR |
| 38 | Q9Y5Q9 | GKLsAEENPDDSEVPSSSGINSTK |
| 39 | Q9Y5J1 | KTSSDDEsEEDEDDLLQR |
| 40 | Q9Y5B6 | MADHLEGLssDDEETSTDITNFNLEK |
| 41 | Q9Y5B6 | MADHLEGLssDDEETSTDITNFNLEK |
| 42 | Q9Y5B6 | IAEEIGIEGsDDDALVTGEQDEELSR |
| 43 | Q9Y580 | SFsSPENFQR |
| 44 | Q9Y570 | QCEGITsPEGSK |
| 45 | Q9Y520 | LPDLsPVENK |
| 46 | Q9Y520 | TPDLSNQNSSDQANEEWEtAsESsDFNER |
| 47 | Q9Y520 | TPDLSNQNSSDQANEEWEtAsESSDFNER |
| 48 | Q9Y520 | TPDLSNQNSSDQANEEWEtAsESSDFNER |
| 49 | Q9Y519 | TLLLssDDEF |
| 50 | Q9Y519 | TLLLssDDEF |
| 51 | Q9Y4P1 | FFDsEDEDFEILSL |
| 52 | Q9Y4H2 | VAsPTSGVK |
| 53 | Q9Y4H2 | HNsASVENVSLR |
| 54 | Q9Y4H2 | sPGEYINIDFGEPGAR |
| 55 | Q9Y4H2 | SNtPEsIAEtPPAR |
| 56 | Q9Y4H2 | SNtPEsIAEtPPAR |
| 57 | Q9Y4H2 | SNtPESIAETPPAR |
| 58 | Q9Y4F1 | VSAGEPGSHPsPAPR |
| 59 | Q9Y4F1 | LGAPENSGIStLER |
| 60 | Q9Y4E8 | GASAATGIPLESDEDsNDNDNDIENENCMHtN |
| 61 | Q9Y4E8 | GASAATGIPLEsDEDSNDNDNDIENENCMHTN |
| 62 | Q9Y4E8 | GPSTPKsPGASNFSTLPK |
| 63 | Q9Y4E1 | GLFsDEEDSEDLFSSQSASNLK |
| 64 | Q9Y4B5 | EDVtPPLsPDDLK |
| 65 | Q9Y4B5 | EDVtPPLsPDDLK |
| 66 | Q9Y4B5 | GRsPsPIGVGSEMCR |
| 67 | Q9Y4B5 | GRsPsPIGVGSEMCR |
| 68 | Q9Y4B5 | DHAPsIPTSPFGDSLESSTELRR |
| 69 | Q9Y4B5 | DHAPSIPTsPFGDSLESSTELR |
| 70 | Q9Y4B5 | EGPVGGEsDSEEMFEK |
| 71 | Q9Y446 | LssGFDDIDLPSAVK |
| 72 | Q9Y446 | LSsGFDDIDLPSAVK |
| 73 | Q9Y446 | sAVDLSCSR |

| | | |
|---|---|---|
| 74 | Q9Y446 | ADYDTLsLR |
| 75 | Q9Y3T9 | EAARsPDKPGGsPSASR |
| 76 | Q9Y3T9 | EAARsPDKPGGSPSASR |
| 77 | Q9Y3T9 | DLFDLNssEEDDTEGFSER |
| 78 | Q9Y3T9 | DLFDLNsSEEDDTEGFSER |
| 79 | Q9Y3Q8 | sPDPFGAVAAQK |
| 80 | Q9Y3P9 | EVLDEDtDEEKETLK |
| 81 | Q9Y3M8 | KKGDDsDEEDLCISNK |
| 82 | Q9Y3E1 | FTGYQAIQQQSSSETEGEGGNTADAssEEEGDRVEEDGK |
| 83 | Q9Y3E1 | FTGYQAIQQQSSSETEGEGGNTADAssEEEGDRVEEDGK |
| 84 | Q9Y3D3 | TDAEAtDTEATET |
| 85 | Q9Y388 | EVQAEQPSSSsPR |
| 86 | Q9Y2X7 | HGsGADSDYENTQSGDPLLGLEGK |
| 87 | Q9Y2X7 | HGsGADsDYENTQSGDPLLGLEGK |
| 88 | Q9Y2X3 | EEPLsEEEPCTSTAIASPEK |
| 89 | Q9Y2X3 | EEPLsEEEPCTSTAIAsPEK |
| 90 | Q9Y2W1 | ERsPALK |
| 91 | Q9Y2W1 | IDIsPSTFR |
| 92 | Q9Y2W1 | ASAVSELsPR |
| 93 | Q9Y2W1 | KsPVGKsPPSTGSTYGSSQK |
| 94 | Q9Y2W1 | EKGSFsDTGLGDGK |
| 95 | Q9Y2W1 | WAHDKFsGEEGEIEDDESGTENR |
| 96 | Q9Y2W1 | FSGEEGEIEDDEsGTENR |
| 97 | Q9Y2W1 | KsPVGKsPPSTGSTYGSSQK |
| 98 | Q9Y2W1 | TDSEKPFRGsQsPK |
| 99 | Q9Y2W1 | TDSEKPFRGsQsPK |
| 100 | Q9Y2V2 | ERsPsPLR |
| 101 | Q9Y2V2 | ERsPsPLR |
| 102 | Q9Y2V2 | ERsPsPLRGNVVPsPLPTR |
| 103 | Q9Y2V2 | TFsATVR |
| 104 | Q9Y2V2 | sRERSPsPLR |
| 105 | Q9Y2U8 | VLLGFSSDEsDVEASPR |
| 106 | Q9Y2U5 | GSDIDNPTLTVMDIsPPSRSPR |
| 107 | Q9Y2K7 | GDEEGLGGEEEEEEEEEEEDDsAEEGGAAR |
| 108 | Q9Y2K7 | LtPVRPAAAsPIVSGAR |
| 109 | Q9Y2K7 | LtPVRPAAAsPIVSGAR |
| 110 | Q9Y2K7 | SCDEPLtPPPHsPTSMLQLIHDPVSPR |
| 111 | Q9Y2K7 | SCDEPLTPPPHsPTSMLQLIHDPVSPR |
| 112 | Q9Y2K7 | SCDEPLtPPPHsPTSMLQLIHDPVsPR |
| 113 | Q9Y2K6 | AVPIAVADEGEsEsEDDDLKPR |
| 114 | Q9Y2K6 | AVPIAVADEGEsEsEDDDLKPR |

| | | |
|---|---|---|
| 115 | Q9Y2H5 | sAVFPGEGK |
| 116 | Q9Y2H5 | sIHEVDISNLEAALR |
| 117 | Q9Y2H5 | MLsVQCATPSPPTsPASPAPPANPLSSESPR |
| 118 | Q9Y2H5 | MLSVQCATPsPPTSPASPAPPANPLSSESPR |
| 119 | Q9Y2H5 | MLSVQCATPSPPtsPASPAPPANPLSSESPR |
| 120 | Q9Y2F5 | RAsPPDPSPSPSAASASER |
| 121 | Q9Y2F5 | LDTGsPEPETR |
| 122 | Q9Y2D5 | QVLQSTQsPR |
| 123 | Q9Y281 | AsGVTVNDEVIK |
| 124 | Q9Y266 | ELtDEEAER |
| 125 | Q9Y241 | STDTGVsLPSYEEDQGSK |
| 126 | Q9UQR0 | RsPQQTVPYVVPLsPK |
| 127 | Q9UQR0 | RsPQQTVPYVVPLsPK |
| 128 | Q9UQN3 | ATIsDEEIER |
| 129 | Q9UQE7 | GDVEGSQsQDEGEGSGESER |
| 130 | Q9UQB8 | SSsMAAGLER |
| 131 | Q9UQB8 | SLsPPQSQSK |
| 132 | Q9UQ80 | MsGEDEQQEQTIAEDLVVTK |
| 133 | Q9UQ35 | sRsPQWR |
| 134 | Q9UQ35 | sRsPQWR |
| 135 | Q9UQ35 | GRSECDssPEPK |
| 136 | Q9UQ35 | GRSECDssPEPK |
| 137 | Q9UQ35 | GRsECDSsPEPK |
| 138 | Q9UQ35 | sRsRsLVR |
| 139 | Q9UQ35 | sRsRsLVR |
| 140 | Q9UQ35 | sRsRsLVR |
| 141 | Q9UQ35 | sRsRtPPAIR |
| 142 | Q9UQ35 | sRsRtPPAIR |
| 143 | Q9UQ35 | sRsPLAIR |
| 144 | Q9UQ35 | sRsPLAIR |
| 145 | Q9UQ35 | VKPEtPPR |
| 146 | Q9UQ35 | ELsNSPLRENSFGsPLEFR |
| 147 | Q9UQ35 | ELSNsPLR |
| 148 | Q9UQ35 | sRTPPVTR |
| 149 | Q9UQ35 | sRsRtPPVTR |
| 150 | Q9UQ35 | sRsRtPPVTR |
| 151 | Q9UQ35 | RsRsVsPCSNVESR |
| 152 | Q9UQ35 | RsRsVsPCSNVESR |
| 153 | Q9UQ35 | EKSAtRPsPsPER |
| 154 | Q9UQ35 | EKSAtRPsPsPER |
| 155 | Q9UQ35 | ARsRtPPSAPSQSR |

| | | |
|---|---|---|
| 156 | Q9UQ35 | ARsRtPPSAPSQSR |
| 157 | Q9UQ35 | SCFESsPDPELK |
| 158 | Q9UQ35 | SCFEssPDPELK |
| 159 | Q9UQ35 | sPVPSAFSDQSR |
| 160 | Q9UQ35 | NHsGSRtPPVALNSSR |
| 161 | Q9UQ35 | NHSGsRtPPVALNSSR |
| 162 | Q9UQ35 | NHSGsRtPPVALNSSR |
| 163 | Q9UQ35 | RsLsGSSPCPK |
| 164 | Q9UQ35 | RsLsGSSPCPK |
| 165 | Q9UQ35 | CRsPGMLEPLGSSR |
| 166 | Q9UQ35 | RsRsVsPCSNVESR |
| 167 | Q9UQ35 | AQtPPGPSLSGSK |
| 168 | Q9UQ35 | RPsPQPSPR |
| 169 | Q9UQ35 | RVPsPTPAPK |
| 170 | Q9UQ35 | SLsYSPVER |
| 171 | Q9UQ35 | SGMsPEQSR |
| 172 | Q9UQ35 | HGGsPQPLATTPLSQEPVNPPSEASPtR |
| 173 | Q9UQ35 | AQTPPGPSLSGSKsPCPQEK |
| 174 | Q9UQ35 | THTTALAGRsPsPASGR |
| 175 | Q9UQ35 | HAsSSPEsPKPAPAPGSHR |
| 176 | Q9UQ35 | HASSsPESPKPAPAPGSHR |
| 177 | Q9UQ35 | THTTALAGRsPsPASGR |
| 178 | Q9UQ35 | HGGSPQPLATTPLSQEPVNPPSEAsPTR |
| 179 | Q9UQ35 | ELSNsPLRENSFGsPLEFR |
| 180 | Q9UQ35 | GEFSAsPMLK |
| 181 | Q9UQ35 | MSCFSRPsMsPTPLDR |
| 182 | Q9UQ35 | MSCFSRPsMsPTPLDR |
| 183 | Q9UQ35 | SLSYsPVER |
| 184 | Q9UQ35 | sCFESsPDPELK |
| 185 | Q9UQ35 | sRTsPITR |
| 186 | Q9UQ35 | sRTsPVTR |
| 187 | Q9UQ35 | sRsRTsPVTR |
| 188 | Q9UQ35 | sRTsPVTR |
| 189 | Q9UQ35 | sRSRtsPITR |
| 190 | Q9UQ35 | QSHSGSIsPYPK |
| 191 | Q9UQ35 | sLSYsPVER |
| 192 | Q9UQ35 | RSSRssPELTR |
| 193 | Q9UQ35 | RSsRSsPELTR |
| 194 | Q9UQ35 | RSSRSsPELTR |
| 195 | Q9UQ35 | RsLsGSsPCPK |
| 196 | Q9UQ35 | EKsATRPsPsPER |

| | | |
|---|---|---|
| 197 | Q9UQ35 | ELSNsPLRENsFGSPLEFR |
| 198 | Q9UQ35 | QGSITsPQANEQSVtPQR |
| 199 | Q9UQ35 | HGGsPQPLATTPLSQEPVNPPsEASPTR |
| 200 | Q9UQ35 | QGSITSPQANEQSVtPQR |
| 201 | Q9UQ35 | AGMSSNQSISsPVLDAVPR |
| 202 | Q9UQ35 | AGMSSNQSISSPVLDAVPRtPSR |
| 203 | Q9UQ35 | SRsSsPVTELASR |
| 204 | Q9UQ35 | GEGDAPFSEPGTTSTQRPsSPETATK |
| 205 | Q9UQ35 | sRsRtPLISR |
| 206 | Q9UQ35 | sRsRtPLISR |
| 207 | Q9UQ35 | sRSRtsPITR |
| 208 | Q9UQ35 | GEGDAPFSEPGTTSTQRPSsPETATK |
| 209 | Q9UQ35 | RssRSSPELTR |
| 210 | Q9UQ35 | sRTsPAPWK |
| 211 | Q9UQ35 | sRTsPAPWK |
| 212 | Q9UQ35 | HASSSPEsPKPAPAPGSHR |
| 213 | Q9UQ35 | GGRsRSSSPVTELASR |
| 214 | Q9UQ35 | SRsSsPVTELASR |
| 215 | Q9UQ35 | sRsRTsPAPWK |
| 216 | Q9UQ35 | sRSssPVTELASR |
| 217 | Q9UQ35 | HGGSPQPLATTPLsQEPVNPPSEAsPTR |
| 218 | Q9UQ35 | tSPPLLDR |
| 219 | Q9UPU7 | DTSPDKGELVsDEEEDT |
| 220 | Q9UPU5 | VSDQNsPVLPK |
| 221 | Q9UPT8 | TGSGSPFAGNsPAR |
| 222 | Q9UPT8 | TGSGsPFAGNSPAR |
| 223 | Q9UPT8 | AAKPGPAEAPSPTASPSGDAsPPATAPYDPR |
| 224 | Q9UPS8 | TLFEDRDsDsQDEVVVESLPTTSIK |
| 225 | Q9UPS8 | TLFEDRDsDsQDEVVVESLPTTSIK |
| 226 | Q9UPR0 | KKtVSFsSMPTEK |
| 227 | Q9UPR0 | KKtVsFSSMPTEK |
| 228 | Q9UPQ0 | sPEPEATLTFPFLDK |
| 229 | Q9UPQ0 | sFQGDDSDLLLK |
| 230 | Q9UPQ0 | DTDDIEsPKR |
| 231 | Q9UPQ0 | SDSLsPPR |
| 232 | Q9UPQ0 | QTPsPDVVLR |
| 233 | Q9UPQ0 | SRQtPsPDVVLR |
| 234 | Q9UPQ0 | sRQTPsPDVVLR |
| 235 | Q9UPQ0 | GSSDGRGsDSESDLPHR |
| 236 | Q9UPQ0 | GSSDGRGSDsESDLPHR |
| 237 | Q9UPP1 | DAEYIYPSLEsDDDDPALK |

| | | |
|---|---|---|
| 238 | Q9UPP1 | DAEYIYPsLEsDDDDPALK |
| 239 | Q9UPN9 | TFAPLPEFEQEEDDGEVtEDsDEDFIQPR |
| 240 | Q9UPN9 | TFAPLPEFEQEEDDGEVtEDsDEDFIQPR |
| 241 | Q9UPN7 | IQQFDDDEEEEDEEEAQGsGESDGEDGAWQGSQLAR |
| 242 | Q9UPN7 | IQQFDDDEEEEDEEEAQGSGEsDGEDGAWQGSQLAR |
| 243 | Q9UPN6 | ETVQTTQsPTPVEK |
| 244 | Q9UPN3 | LLDAEDVDVPsPDEK |
| 245 | Q9UPN3 | AFLAELEQNsPK |
| 246 | Q9UP95 | ESsPFLsPLEASR |
| 247 | Q9UP95 | ESsPFLsPLEASR |
| 248 | Q9UNZ2 | KKsPNELVDDLFK |
| 249 | Q9UNS2 | SMGSQEDDSGNKPSSYs |
| 250 | Q9UNF1 | HLDGEEDGSsDQsQASGTTGGR |
| 251 | Q9UNF1 | HLDGEEDGSsDQSQASGTTGGR |
| 252 | Q9UNF1 | HLDGEEDGsSDQSQASGTTGGR |
| 253 | Q9UNF1 | sDTSESGAGLTR |
| 254 | Q9UNE7 | LGAGGGsPEK |
| 255 | Q9UNE7 | LGAGGGsPEKsPSAQELK |
| 256 | Q9UN86 | YEDEVFGDsEPELDEEsEDEVEEEQEER |
| 257 | Q9UN86 | YEDEVFGDSEPELDEEsEDEVEEEQEER |
| 258 | Q9UMZ2 | SLsLGDKEISR |
| 259 | Q9ULX3 | GEDVPsEEEEEEENGFEDR |
| 260 | Q9ULX3 | KDDsDDDGGGWITPSNIK |
| 261 | Q9ULW0 | SSDQPLTVPVsPK |
| 262 | Q9ULU4 | DKAsPEPEKDFSEK |
| 263 | Q9ULU4 | TGQAGsLSGSPKPFSPQLSAPITTK |
| 264 | Q9ULU4 | TGQAGSLSGsPKPFSPQLSAPITTK |
| 265 | Q9ULU4 | STsPAsEKADPGAVK |
| 266 | Q9ULU4 | STsPAsEKADPGAVK |
| 267 | Q9ULL5 | KQETAAVCGEtDEEAGEsGGEGIFR |
| 268 | Q9ULL5 | KQETAAVCGEtDEEAGEsGGEGIFR |
| 269 | Q9ULL5 | NLETLPSFssDEEDSVAK |
| 270 | Q9ULL5 | NLETLPSFssDEEDSVAK |
| 271 | Q9ULJ3 | SFsASQSTDR |
| 272 | Q9ULJ3 | ESEVCPVPTNsPsPPPLPPPPPLPK |
| 273 | Q9ULJ3 | ESEVCPVPTNsPsPPPLPPPPPLPK |
| 274 | Q9ULI4 | LGQsPPGR |
| 275 | Q9ULH1 | RTLsDPPsPLPHGPPNK |
| 276 | Q9ULH1 | RTLsDPPsPLPHGPPNK |
| 277 | Q9ULH0 | LPsDEDEsGTEESDNTPLLK |
| 278 | Q9ULH0 | LPSDEDEsGTEESDNTPLLK |

| | | |
|---|---|---|
| 279 | Q9ULD4 | RsPsPYSLK |
| 280 | Q9ULD4 | RsPsPYSLK |
| 281 | Q9UL03 | sHEEVNTELK |
| 282 | Q9UKV3 | KsLsPGVSR |
| 283 | Q9UKV3 | KsLsPGVSR |
| 284 | Q9UKV3 | AAKLsEGSQPAEEEEDQETPSR |
| 285 | Q9UKV3 | AAKLsEGsQPAEEEEDQETPSR |
| 286 | Q9UKV3 | TAQVPsPPR |
| 287 | Q9UKV3 | KSSSISEEKGDsDDEKPR |
| 288 | Q9UKV3 | AEsPAEKVPEESVLPLVQK |
| 289 | Q9UKV3 | EKsKsPsPPR |
| 290 | Q9UKV3 | EKsKsPsPPR |
| 291 | Q9UKV3 | RLsQPESAEK |
| 292 | Q9UKV3 | GVPAGNsDTEGGQPGR |
| 293 | Q9UKS6 | DLHQGIEAAsDEEDLR |
| 294 | Q9UKS6 | ALYDYAGQEADELsFR |
| 295 | Q9UKS6 | GGRsPDEVTLTSIVPTR |
| 296 | Q9UKS6 | DGTAPPPQSPGSPGTGQDEEWsDEESPR |
| 297 | Q9UKS6 | DGTAPPPQsPGSPGTGQDEEWsDEESPR |
| 298 | Q9UKS6 | GGRsPDEVTLTsIVPTR |
| 299 | Q9UKS6 | SPDEVtLTsIVPTR |
| 300 | Q9UKM9 | DDGDEEGLLtHsEEELEHSQDTDADDGALQ |
| 301 | Q9UKM9 | DDGDEEGLLtHsEEELEHSQDTDADDGALQ |
| 302 | Q9UKM9 | DDGDEEGLLtHsEEELEHSQDtDADDGALQ |
| 303 | Q9UKM9 | TRDDGDEEGLLTHsEEELEHsQDTDADDGALQ |
| 304 | Q9UKL0 | EEsEDELEEANGNNPIDIEVDQNK |
| 305 | Q9UKJ3 | sWGHEsPEER |
| 306 | Q9UKJ3 | sWGHEsPEER |
| 307 | Q9UKJ3 | SQsPHYFR |
| 308 | Q9UKJ3 | KPsVSEEVQATPNK |
| 309 | Q9UK76 | NsSEASSGDFLDLK |
| 310 | Q9UK76 | EDLESsGLQR |
| 311 | Q9UK61 | LIPITGGNARsPEDQLGK |
| 312 | Q9UK59 | RLsDEHEPEQR |
| 313 | Q9UK58 | AEEKsPISINVK |
| 314 | Q9UK58 | GLNPDGTPALSTLGGFsPASKPSSPR |
| 315 | Q9UK58 | GLNPDGTPALSTLGGFsPASKPSsPR |
| 316 | Q9UK58 | GLNPDGTPALSTLGGFsPAsKPSSPR |
| 317 | Q9UJX5 | IKEEVLsEsEAENQQAGAAALAPEIVIK |
| 318 | Q9UJX5 | IKEEVLsEsEAENQQAGAAALAPEIVIK |
| 319 | Q9UJX2 | RVsPLNLSSVTP |

| | | |
|---|---|---|
| 320 | Q9UJX2 | RVsPLNLSSVtP |
| 321 | Q9UJV9 | ARTDEVPAGGsRsEAEDEDDEDYVPYVPLR |
| 322 | Q9UJ70 | SLGLSLsGGDQEDAGR |
| 323 | Q9UIS9 | LLPSVWSEsEDGAGsPPPYR |
| 324 | Q9UIS9 | LLPSVWsESEDGAGsPPPYR |
| 325 | Q9UIK4 | NCEsDTEEDIAR |
| 326 | Q9UID3 | AAAAAAGPSPGSGPGDsPEGPEGEAPER |
| 327 | Q9UI30 | GIPNMLLsEEETES |
| 328 | Q9UHY1 | sEGESQTVLSSGSDPK |
| 329 | Q9UHY1 | SEGESQTVLsSGSDPK |
| 330 | Q9UHY1 | VESSSSAPGLTSVSPPVTSTTSAASPEEEEEsEDESEILEESPCGR |
| 331 | Q9UHY1 | TPtPEPAEVETR |
| 332 | Q9UHW9 | LTSIGsDEDEETETYQEK |
| 333 | Q9UHR5 | KNVLSSLAVYAEDsEPEsDGEAGIEAVGSAAEEK |
| 334 | Q9UHR5 | KNVLSSLAVYAEDsEPEsDGEAGIEAVGSAAEEK |
| 335 | Q9UHR4 | LLEENETEAVTVPTPsPTPVR |
| 336 | Q9UHR4 | TPASTPVSGTPQAsPMIER |
| 337 | Q9UHI6 | NNsVSGLSVK |
| 338 | Q9UHI6 | SYLEGssDNQLKDSESTPVDDR |
| 339 | Q9UHI6 | SYLEGSsDNQLKDSESTPVDDR |
| 340 | Q9UHD8 | sFEVEEVETPNSTPPR |
| 341 | Q9UHB7 | NSYNNSQAPsPGLGSK |
| 342 | Q9UHB7 | ELLsPLSEPDDRyPLIVK |
| 343 | Q9UHB7 | ELLsPLsEPDDRYPLIVK |
| 344 | Q9UHB6 | ETPHsPGVEDAPIAK |
| 345 | Q9UHB6 | SEVQQPVHPKPLsPDSR |
| 346 | Q9UHB6 | SNtENLSQHFR |
| 347 | Q9UH62 | YNDWsDDDDDSNESK |
| 348 | Q9UGV2 | HQTMEVsC |
| 349 | Q9UGV2 | SVTSNQsDGTQESCESPDVLDR |
| 350 | Q9UGU0 | DLPsPDSR |
| 351 | Q9UGH3 | SSLAETLDsTGsLDPQR |
| 352 | Q9UGH3 | SSLAETLDsTGsLDPQR |
| 353 | Q9UFC0 | ACAsPSAQVEGsPVAGSDGSQPAVK |
| 354 | Q9UFC0 | ACAsPSAQVEGSPVAGSDGSQPAVK |
| 355 | Q9UER7 | TSVATQCDPEEIIVLsDsD |
| 356 | Q9UER7 | TSVATQCDPEEIIVLsDsD |
| 357 | Q9UDY2 | KVQVAALQAsPPLDQDDR |
| 358 | Q9UDY2 | AASSDQLRDNsPPPAFKPEPPK |
| 359 | Q9UDY2 | GRsIDQDYER |
| 360 | Q9UDY2 | SILKPSTPIPPQEGEEVGEsSEEQDNAPK |

| | | |
|---|---|---|
| 361 | Q9UDY2 | SILKPSTPIPPQEGEEVGESsEEQDNAPK |
| 362 | Q9UDY2 | DSQQTLINIPSLNDsDsEIEDISEIESNR |
| 363 | Q9UDY2 | DSQQTLINIPSLNDsDsEIEDISEIESNR |
| 364 | Q9UDY2 | GSYGsDAEEEEYR |
| 365 | Q9UDY2 | AYsPEYR |
| 366 | Q9UDY2 | LIsDFEDTDGEGGAYtDNELDEPAEEPLVSSITR |
| 367 | Q9UDY2 | LISDFEDTDGEGGAytDNELDEPAEEPLVSSITR |
| 368 | Q9UDY2 | LISDFEDtDGEGGAYTDNELDEPAEEPLVSSITR |
| 369 | Q9UDY2 | LISDFEDTDGEGGAytDNELDEPAEEPLVSSITR |
| 370 | Q9UDY2 | SRsWEDsPER |
| 371 | Q9UDY2 | SRsWEDSPER |
| 372 | Q9UBW7 | DIYDKDNYELDEDtD |
| 373 | Q9UBT2 | SITNGSDDGAQPSTSTAQEQDDVLIVDsDEEDSSNNADVSEEER |
| 374 | Q9UBM7 | AKsLDGVTNDR |
| 375 | Q9UBL6 | SLGVPAGEAsPGCtP |
| 376 | Q9UBL6 | SLGVPAGEAsPGCtP |
| 377 | Q9UBK8 | GQEEISGALPVAsPASSR |
| 378 | Q9UBF8 | sVENLPECGITHEQR |
| 379 | Q9UBC2 | STPSHGSVSSLNSTGSLsPK |
| 380 | Q9UBC2 | STPSHGSVSSLNSTGsLsPK |
| 381 | Q9UBB9 | GAAEEAELEDsDDEEKPVK |
| 382 | Q9P2P5 | ANsDTDLVTSESR |
| 383 | Q9P2I0 | EADIDssDEsDIEEDIDQPSAHK |
| 384 | Q9P2I0 | EADIDssDEsDIEEDIDQPSAHK |
| 385 | Q9P2I0 | EADIDssDEsDIEEDIDQPSAHK |
| 386 | Q9P2E9 | SHVEDGDIAGAPASsPEAPPAEQDPVQLK |
| 387 | Q9P2E9 | SHVEDGDIAGAPAssPEAPPAEQDPVQLK |
| 388 | Q9P2D1 | NIPsPGQLDPDTR |
| 389 | Q9P2B4 | VSSPLSPLsPGIKsPTIPR |
| 390 | Q9P2B4 | VSSPLsPLSPGIKsPTIPR |
| 391 | Q9P260 | AAMAPGGSGSGGGVNPFLsDsDEDDDEVAATEER |
| 392 | Q9P260 | AAMAPGGSGSGGGVNPFLsDsDEDDDEVAATEER |
| 393 | Q9P206 | GLAGPPAsPGK |
| 394 | Q9P206 | ALsGRAsPVPAPSSGLHAAVR |
| 395 | Q9P206 | ALsGRAsPVPAPSSGLHAAVR |
| 396 | Q9P206 | DQsPPPsPPPSYHPPPPPTK |
| 397 | Q9P206 | DQsPPPsPPPSYHPPPPPTK |
| 398 | Q9P206 | GsPSGGSTAEASDTLSIR |
| 399 | Q9P206 | FSSVSsPQPR |
| 400 | Q9P206 | KPsVGVPPPAsPSYPR |
| 401 | Q9P206 | KPsVGVPPPAsPSYPR |

| | | |
|---|---|---|
| 402 | Q9P206 | GGWDHGDTQsIQsSR |
| 403 | Q9P206 | GGWDHGDTQSIQsSR |
| 404 | Q9P206 | FSSVssPQPR |
| 405 | Q9P206 | GsPSGGsTAEASDTLSIR |
| 406 | Q9P1Y6 | AEAPSsPDVAPAGK |
| 407 | Q9P1Y6 | AEAPssPDVAPAGK |
| 408 | Q9P1Y6 | EAsPAPLAQGEPGR |
| 409 | Q9P1Y6 | ECsPTSSLER |
| 410 | Q9P1Y5 | DLPDGHAAsPR |
| 411 | Q9P107 | SLDsPTSsPGAGTR |
| 412 | Q9P107 | SLDsPTSsPGAGTR |
| 413 | Q9P107 | GPsPAAASPEGSPLR |
| 414 | Q9P107 | GPSPAAAsPEGsPLR |
| 415 | Q9P035 | WLDEsDAEMELR |
| 416 | Q9NZT2 | sVEPQDAGPLER |
| 417 | Q9NZT2 | SQGDEAGGHGEDRPEPLsPK |
| 418 | Q9NZT2 | KVEEEGsPGDPDHEASTQGR |
| 419 | Q9NZN8 | TNSMSSSGLGsPNR |
| 420 | Q9NZM3 | TVsPGSVsPIHGQGQVVENLK |
| 421 | Q9NZM3 | TVsPGSVsPIHGQGQVVENLK |
| 422 | Q9NZI8 | QGsPVAAGAPAK |
| 423 | Q9NZD8 | GSLGIsQEEQ |
| 424 | Q9NZ63 | GDsEsEEDEQDSEEVR |
| 425 | Q9NZ63 | GDsEsEEDEQDSEEVR |
| 426 | Q9NYV6 | EGDVDVsDsDDEDDNLPANFDTCHR |
| 427 | Q9NYV6 | EGDVDVsDsDDEDDNLPANFDTCHR |
| 428 | Q9NYV4 | HLLTDLPLPPELPGGDLsPPDsPEPK |
| 429 | Q9NYV4 | HLLTDLPLPPELPGGDLsPPDsPEPK |
| 430 | Q9NYV4 | SSSPFLSKRsLsR |
| 431 | Q9NYV4 | SSSPFLSKRsLsR |
| 432 | Q9NYV4 | sLsRsPLPSR |
| 433 | Q9NYV4 | QRsVsPYSR |
| 434 | Q9NYV4 | QRsVsPYSR |
| 435 | Q9NYV4 | HSsIsPVR |
| 436 | Q9NYV4 | HSsIsPVR |
| 437 | Q9NYV4 | RRSssPFLSK |
| 438 | Q9NYV4 | RRsssPFLSK |
| 439 | Q9NYL2 | YQQITPVNQsR |
| 440 | Q9NYL2 | SSsPTQYGLTK |
| 441 | Q9NYF8 | AEGEPQEEsPLK |
| 442 | Q9NYF8 | IDIsPSTLR |

| | | |
|---|---|---|
| 443 | Q9NYF8 | ETQsPEQVK |
| 444 | Q9NYF8 | FNDsEGDDTEETEDYR |
| 445 | Q9NYF8 | NtPSQHSHSIQHsPER |
| 446 | Q9NYF8 | NTPSQHSHSIQHsPER |
| 447 | Q9NYF8 | FNDsEGDDtEETEDYR |
| 448 | Q9NYF8 | YSPsQNsPIHHIPSR |
| 449 | Q9NYF8 | YsPSQNSPIHHIPSR |
| 450 | Q9NYF8 | AEGEWEDQEALDYFsDK |
| 451 | Q9NYF8 | EKsTFREEsPLR |
| 452 | Q9NYF8 | EKsTFREEsPLR |
| 453 | Q9NYF8 | DLFDYsPPLHK |
| 454 | Q9NYF8 | ySPSQNsPIHHIPSR |
| 455 | Q9NYF3 | FsLsPSLGPQASR |
| 456 | Q9NYF3 | FsLsPSLGPQASR |
| 457 | Q9NYD6 | TEQSLAGPKGsPSEsEKER |
| 458 | Q9NYD6 | TEQSLAGPKGsPSEsEKER |
| 459 | Q9NYB0 | YLLGDAPVsPSSQK |
| 460 | Q9NY61 | YLVDGTKPNAGsEEIssEDDELVEEK |
| 461 | Q9NY61 | YLVDGTKPNAGSEEIssEDDELVEEK |
| 462 | Q9NY61 | YLVDGTKPNAGSEEIssEDDELVEEK |
| 463 | Q9NY27 | NHSDSSTSESEVSSVsPLK |
| 464 | Q9NXV6 | GIsSSNEGVEEPSK |
| 465 | Q9NXG2 | DQQPsGsEGEDDDAEAALK |
| 466 | Q9NXG2 | DQQPsGsEGEDDDAEAALK |
| 467 | Q9NWZ8 | EDQALSKEEEMEtEsDAEVECDLSNMEITEELR |
| 468 | Q9NWZ8 | EDQALSKEEEMEtEsDAEVECDLSNMEITEELR |
| 469 | Q9NWZ5 | YFGTDAVPDGsDEEEVAYTG |
| 470 | Q9NWW5 | HGsVSADEAAR |
| 471 | Q9NWV8 | sEGEGEAASADDGSLNTSGAGPK |
| 472 | Q9NWV8 | MEVAEPSsPTEEEEEEEEHSAEPRPR |
| 473 | Q9NWV8 | MEVAEPssPTEEEEEEEEHSAEPRPR |
| 474 | Q9NWH9 | DGQDAIAQsPEK |
| 475 | Q9NWH9 | ISsKsPGHMVILDQTK |
| 476 | Q9NW82 | TMFAQVEsDDEEAK |
| 477 | Q9NW75 | DHsDsDDQMLVAK |
| 478 | Q9NW75 | DHsDsDDQMLVAK |
| 479 | Q9NVU7 | YIEIDsDEEPR |
| 480 | Q9NVU0 | EAANEAGDsSQDEAEDDVK |
| 481 | Q9NVU0 | EAANEAGDssQDEAEDDVK |
| 482 | Q9NVN3 | GDNWYsEDEDTDTEEYK |
| 483 | Q9NVN3 | GDNWYsEDEDtDTEEYK |

| | | |
|---|---|---|
| 484 | Q9NVM6 | QAQAQEsEEEEESR |
| 485 | Q9NVD7 | ATSPQKsPSVPKsPTPK |
| 486 | Q9NVD7 | SPSVPKsPTPKsPPSR |
| 487 | Q9NV70 | LTGSTSsLNK |
| 488 | Q9NV56 | VLTANsNPSsPSAAK |
| 489 | Q9NV56 | VLTANSNPSsPSAAK |
| 490 | Q9NUN5 | KSVIEGVDEDsDIsDDEPSVYSA |
| 491 | Q9NUN5 | KSVIEGVDEDsDIsDDEPSVYSA |
| 492 | Q9NTZ6 | sRsPHEAGFCVYLK |
| 493 | Q9NTZ6 | sRsPHEAGFCVYLK |
| 494 | Q9NTJ3 | TEsPATAAETASEELDNR |
| 495 | Q9NTJ3 | EEGPPPPSPDGASsDAEPEPPSGR |
| 496 | Q9NTJ3 | EEGPPPPsPDGASsDAEPEPPSGR |
| 497 | Q9NTJ3 | EEGPPPPSPDGAssDAEPEPPSGR |
| 498 | Q9NTI5 | EDILENEDEQNsPPK |
| 499 | Q9NTI5 | AEsPESSAIESTQSTPQK |
| 500 | Q9NTI5 | AEsPESSAIESTQStPQK |
| 501 | Q9NTI5 | METVSNASSSSNPSsPGR |
| 502 | Q9NTI5 | GRLDSsEMDHsENEDYTMSSPLPGK |
| 503 | Q9NTI5 | ENDSSEEVDVFQGSsPVDDIPQEETEEEEVSTVNVR |
| 504 | Q9NTI5 | GHTAsESDEQQWPEEK |
| 505 | Q9NS91 | NDLQDTEIsPR |
| 506 | Q9NS87 | SDKNQQGFsPK |
| 507 | Q9NS69 | AAAVAAAGAGEPQsPDELLPK |
| 508 | Q9NRZ9 | ILENSEDsSPECLF |
| 509 | Q9NRY4 | TSFSVGsDDELGPIR |
| 510 | Q9NRY4 | AGsPLCNSNLQDsEEDIEPSYSLFR |
| 511 | Q9NRY4 | AGsPLCNSNLQDsEEDIEPSYSLFR |
| 512 | Q9NRX5 | SDGsLEDGDDVHR |
| 513 | Q9NRG9 | FsPVLGR |
| 514 | Q9NRG0 | EEDEENDNDNEsDHDEADS |
| 515 | Q9NR30 | NEEPsEEEIDAPKPK |
| 516 | Q9NR30 | EEPSQNDIsPK |
| 517 | Q9NR30 | AEPSEVDMNsPK |
| 518 | Q9NR30 | LKNGFPHPEPDCNPSEAASEEsNsEIEQEIPVEQK |
| 519 | Q9NR30 | LKNGFPHPEPDCNPSEAAsEESNsEIEQEIPVEQK |
| 520 | Q9NR30 | LKNGFPHPEPDCNPsEAASEESNSEIEQEIPVEQK |
| 521 | Q9NR19 | SWsPPPEVSR |
| 522 | Q9NQZ2 | TSAAACAVTDLsDDSDFDEK |
| 523 | Q9NQZ2 | TSAAACAVTDLsDDsDFDEK |
| 524 | Q9NQZ2 | TSAAACAVtDLSDDsDFDEKAK |

| | | |
|---|---|---|
| 525 | Q9NQW6 | ARQPLSEASNQQPLsGGEEK |
| 526 | Q9NQW6 | SCEGQNPELLPKtPIsPLK |
| 527 | Q9NQW6 | TQsLPVTEK |
| 528 | Q9NQW6 | STLsQTVPSK |
| 529 | Q9NQS7 | IAQVsPGPR |
| 530 | Q9NQG6 | AIsAPTsPTR |
| 531 | Q9NQG6 | AIsAPTsPTR |
| 532 | Q9NQC3 | MEDLDQsPLVSSSDsPPRPQPAFK |
| 533 | Q9NQC3 | MEDLDQSPLVSSSDsPPRPQPAFK |
| 534 | Q9NQ55 | VGGsDEEASGIPSR |
| 535 | Q9NQ55 | LQDISELLATGAGLsEsEAEPDGDHNITELPQAVAGR |
| 536 | Q9NQ55 | LQDISELLATGAGLsEsEAEPDGDHNITELPQAVAGR |
| 537 | Q9NQ29 | RsEEKEAGEI |
| 538 | Q9NPQ8 | GLMAGGRPEGQYsEDEDtDTDEYK |
| 539 | Q9NPQ8 | GLMAGGRPEGQYSEDEDtDTDEYKEAK |
| 540 | Q9NPE2 | EPDPDsDWEPEER |
| 541 | Q9HCN4 | DSLsPVLHPSDLILTR |
| 542 | Q9HCN4 | GTLDEEDEEADsDTDDIDHR |
| 543 | Q9HCH0 | GPsPEPPPsPLQVPTYPQLTLEVPQAPEVLR |
| 544 | Q9HCH0 | GPsPEPPPsPLQVPTYPQLTLEVPQAPEVLR |
| 545 | Q9HCD6 | EYPsPPPsPLR |
| 546 | Q9HCD6 | EYPsPPPsPLR |
| 547 | Q9HCD6 | SCDELsPVsPTQGGYPSEPTR |
| 548 | Q9HCD6 | SCDELsPVsPTQGGYPSEPTR |
| 549 | Q9HCD6 | DCSYGAVTsPTSTLESR |
| 550 | Q9HCD5 | DRsPIRGsPR |
| 551 | Q9HCD5 | DRsPIRGsPR |
| 552 | Q9HCD5 | MNTAPSRPsPTR |
| 553 | Q9HC78 | DSQAEPTQPEQAAEAPAEGGPQTNQLETGASsPER |
| 554 | Q9HC52 | VDDKPSsPGDSSK |
| 555 | Q9HC35 | AsPSPQPSSQPLQIHR |
| 556 | Q9HC35 | ASPsPQPSSQPLQIHR |
| 557 | Q9HC35 | APVSSTESVIQSNTPtPPPSQPLNETAEEESR |
| 558 | Q9HB90 | SLQYGAEETPLAGsYGAADSFPK |
| 559 | Q9HAF1 | REPGsGtEsDTSPDFHNQENEPSQEDPEDLDGSVQGVKPQK |
| 560 | Q9HAF1 | REPGSGtEsDtSPDFHNQENEPSQEDPEDLDGSVQGVKPQK |
| 561 | Q9HAF1 | REPGSGtEsDtSPDFHNQENEPSQEDPEDLDGSVQGVKPQK |
| 562 | Q9H9J4 | HQQDsDLSAACsDADLHR |
| 563 | Q9H9J4 | HQQDsDLSAACsDADLHR |
| 564 | Q9H9J4 | HQQDsDLsAACSDADLHR |
| 565 | Q9H9J4 | sEQKDPLEEPK |

| | | |
|---|---|---|
| 566 | Q9H910 | TSDIFGsPVTATSR |
| 567 | Q9H8Y8 | VGDSTPVSEKPVSAAVDANASEsP |
| 568 | Q9H8W4 | SPLNDMsDDDDDDDSSD |
| 569 | Q9H8W4 | SPLNDMsDDDDDDDSsD |
| 570 | Q9H8U3 | SCGTDSQSENEAsPVK |
| 571 | Q9H8U3 | SCGTDSQsENEAsPVK |
| 572 | Q9H814 | ALEVGDMEDGQLsDsDSDMTVAPSDRPLQLPK |
| 573 | Q9H814 | ALEVGDMEDGQLSDsDsDMTVAPSDRPLQLPK |
| 574 | Q9H7Z6 | AAQGAAAAVAAGTSGVAGEGEPGPGENAAAEGTAPSPGRVsPPtPAR |
| 575 | Q9H7Z6 | AAQGAAAAVAAGTSGVAGEGEPGPGENAAAEGTAPSPGRVsPPtPAR |
| 576 | Q9H7Z6 | AAQGAAAAVAAGTSGVAGEGEPGPGENAAAEGTAPsPGRVSPPtPAR |
| 577 | Q9H7P9 | LESSDLtPPHSPPPSSR |
| 578 | Q9H7P9 | LESSDLtPPHsPPPSSR |
| 579 | Q9H7N4 | DREsRsPFLKPDER |
| 580 | Q9H7N4 | QRsPsPAPAPAPAAAAGPPTR |
| 581 | Q9H7N4 | QRsPsPAPAPAPAAAAGPPTR |
| 582 | Q9H7N4 | RRsAsPPPATSSSSSSR |
| 583 | Q9H7N4 | RRsAsPPPATSSSSSSR |
| 584 | Q9H7N4 | EVLYDsEGLsGEER |
| 585 | Q9H7N4 | EVLYDsEGLsGEER |
| 586 | Q9H7N4 | GAEETSWsGEER |
| 587 | Q9H7L9 | SAAGLLAPAPAQAGAPPAPEYYPEEDEELEsAEDDER |
| 588 | Q9H7L9 | SAAGLLAPAPAQAGAPPAPEYYPEEDEELEsAEDDERsCR |
| 589 | Q9H7L9 | RPASPssPEHLPATPAESPAQR |
| 590 | Q9H7L9 | RPAsPSsPEHLPATPAESPAQR |
| 591 | Q9H7L9 | RPASPssPEHLPATPAESPAQR |
| 592 | Q9H7D0 | LSPFHGSsPPQSTPLsPPPLtPK |
| 593 | Q9H7D0 | LSPFHGSSPPQSTPLsPPPLtPK |
| 594 | Q9H7D0 | LSPFHGSSPPQSTPLsPPPLtPK |
| 595 | Q9H7D0 | NsTELAPPLPVR |
| 596 | Q9H792 | sAPTSPTATNISSK |
| 597 | Q9H788 | RLsLGAQK |
| 598 | Q9H788 | TLsSSAQEDIIR |
| 599 | Q9H6Z4 | MPAPEPGAAPsNEEDDSDDDDVLAPSGATAAGAGDEGDGQTTGST |
| 600 | Q9H6Z4 | MPAPEPGAAPSNEEDDsDDDDVLAPSGATAAGAGDEGDGQTTGST |
| 601 | Q9H6Z4 | SAGGSSPEGGEDsDREDGNYCPPVK |
| 602 | Q9H6Y2 | TCEERPAEDGsDEEDPDSMEAPTR |
| 603 | Q9H6T3 | ILDELDKDDSTHESLsQEsESEEDGIHVDSQK |
| 604 | Q9H6T3 | ILDELDKDDSTHESLsQEsESEEDGIHVDSQK |
| 605 | Q9H6H4 | AGGLQDsDTEDECWsDTEAVPR |
| 606 | Q9H6H4 | AGGLQDsDtEDECWSDTEAVPR |

| | | |
|---|---|---|
| 607 | Q9H6H4 | AGGLQDsDTEDECWsDTEAVPR |
| 608 | Q9H6F5 | LQQGAGLESPQGQPEPGAAsPQR |
| 609 | Q9H6F5 | LQQGAGLEsPQGQPEPGAAsPQR |
| 610 | Q9H6F5 | AGLGsPERPPK |
| 611 | Q9H6F5 | ALVEFESNPEETREPGsPPSVQR |
| 612 | Q9H6F5 | LGGLRPEsPESLTSVSR |
| 613 | Q9H6F5 | QPEYSPEsPR |
| 614 | Q9H6F5 | AGLGSPERPPKTsPGsPR |
| 615 | Q9H694 | sPSHSGNAGDLK |
| 616 | Q9H694 | VLSANHGDPSIQTSGSEQTsPK |
| 617 | Q9H694 | VLSANHGDPSIQTSGsEQTsPK |
| 618 | Q9H5H4 | sPGLVPPsPEFAPR |
| 619 | Q9H5H4 | sPGLVPPsPEFAPR |
| 620 | Q9H501 | ALAEEAsEEELPsDVDLNDPYFAEEVK |
| 621 | Q9H501 | ALAEEAsEEELPsDVDLNDPYFAEEVK |
| 622 | Q9H501 | DGTsPEEEIEIER |
| 623 | Q9H501 | FKIDSNIsPK |
| 624 | Q9H4L7 | RNDDIsELEDLsELEDLKDAK |
| 625 | Q9H4L7 | RNDDIsELEDLsELEDLKDAK |
| 626 | Q9H4L7 | DTVIIVsEPsEDEESQGLPTMAR |
| 627 | Q9H4L7 | DTVIIVsEPsEDEEsQGLPTMAR |
| 628 | Q9H4L7 | DTVIIVsEPsEDEESQGLPTMAR |
| 629 | Q9H4L7 | GIQYIDLssDSEDVVSPNCSNTVQEK |
| 630 | Q9H4L7 | GIQYIDLSsDSEDVVSPNCSNTVQEK |
| 631 | Q9H4L7 | KLsSSSEPYEEDEFNDDQSIK |
| 632 | Q9H4L4 | NHLsPQQGGATPQVPSPCCR |
| 633 | Q9H4L4 | NHLsPQQGGATPQVPsPCCR |
| 634 | Q9H4G0 | DYSEADGLsER |
| 635 | Q9H4G0 | SLsPIIGK |
| 636 | Q9H4G0 | APEsDTGDEDQDQER |
| 637 | Q9H4G0 | SLDGAEFsRPAsVSENHDAGPDGDKR |
| 638 | Q9H4G0 | SLDGAEFsRPAsVSENHDAGPDGDKR |
| 639 | Q9H4G0 | LPSSPAsPSPK |
| 640 | Q9H4G0 | RLPSSPAsPsPK |
| 641 | Q9H4G0 | SLPELDRDKsDsDTEGLLFSR |
| 642 | Q9H4G0 | DKSDsDTEGLLFSR |
| 643 | Q9H4A4 | ASGEHsPGSGAAR |
| 644 | Q9H4A3 | DVDDGSGsPHSPHQLSSK |
| 645 | Q9H4A3 | DVDDGsGSPHsPHQLSSK |
| 646 | Q9H4A3 | DVDDGSGSPHsPHQLSSK |
| 647 | Q9H410 | THDHQLESSLsPVEVFAK |

| | | |
|---|---|---|
| 648 | Q9H3R2 | HSsMPRPDY |
| 649 | Q9H3Q1 | AGPDLPSLPSHALEDEGWAAAAPSPGsAR |
| 650 | Q9H3Q1 | AGPDLPSLPSHALEDEGWAAAAPsPGSAR |
| 651 | Q9H3Q1 | AGEPDGEsLDEQPSSSSSK |
| 652 | Q9H3Q1 | sLSSsPVKK |
| 653 | Q9H3Q1 | SLSSsPVKK |
| 654 | Q9H3N1 | KVEEEQEADEEDVsEEEAESK |
| 655 | Q9H307 | GFsDSGGGPPAK |
| 656 | Q9H307 | ESRQEsDPEDDDVKKPALQSSVVATSK |
| 657 | Q9H2Y7 | AAHVPENsDtEQDVLTVKPVR |
| 658 | Q9H2Y7 | AAHVPENsDtEQDVLTVKPVR |
| 659 | Q9H2Y7 | SLsESSVIMDR |
| 660 | Q9H2Y7 | ATGDGssPELPSLER |
| 661 | Q9H2Y7 | ATGDGssPELPSLER |
| 662 | Q9H2Y7 | SLsESsVIMDR |
| 663 | Q9H2P0 | LMHNAsDSEVDQDDVVEWK |
| 664 | Q9H2P0 | LMHNAsDsEVDQDDVVEWK |
| 665 | Q9H2H9 | RsLTNsHLEK |
| 666 | Q9H2H9 | RsLTNsHLEK |
| 667 | Q9H2H9 | SGLELTELQNMTVPEDDNIsNDSNDFtEVENGQINSK |
| 668 | Q9H2H9 | SGLELTELQNMTVPEDDNIsNDsNDFTEVENGQINSK |
| 669 | Q9H2G2 | DVISNTSDVIGTCEAADVAQKVDEDSAEDTQsNDGK |
| 670 | Q9H1E3 | EEDEEPEsPPEK |
| 671 | Q9H1E3 | EKtPsPKEEDEEPESPPEK |
| 672 | Q9H1E3 | EKtPsPKEEDEEPESPPEK |
| 673 | Q9H1E3 | NSQEDsEDSEDKDVK |
| 674 | Q9H1E3 | NSQEDSEDsEDKDVK |
| 675 | Q9H1E3 | KVVDYSQFQEsDDADEDYGR |
| 676 | Q9H1E3 | ATVTPsPVK |
| 677 | Q9H1E3 | ATVtPsPVK |
| 678 | Q9H1E3 | SGKNsQEDsEDSEDKDVK |
| 679 | Q9H1B7 | KAsPEPPDSAEGALK |
| 680 | Q9H1B7 | NSSsPVsPASVPGQR |
| 681 | Q9H1B7 | RNsSSPVsPASVPGQR |
| 682 | Q9H1B7 | NSSsPVsPASVPGQR |
| 683 | Q9H1A4 | NFDFEGsLsPVIAPK |
| 684 | Q9H1A4 | NFDFEGSLsPVIAPK |
| 685 | Q9H0W8 | WKEPGSGGPQNLsGPGGR |
| 686 | Q9H0W8 | SESGHsQPGLYGIER |
| 687 | Q9H0H5 | sIGSAVDQGNESIVAK |
| 688 | Q9H0G5 | VEENPDADsDFDAK |

| | | |
|---|---|---|
| 689 | Q9H0G5 | VEENPDADsDFDAKssADDEIEETR |
| 690 | Q9H0G5 | VEENPDADsDFDAKsSADDEIEETR |
| 691 | Q9H0D6 | AEDsDsEPEPEDNVR |
| 692 | Q9H0D6 | AEDsDsEPEPEDNVR |
| 693 | Q9H0D6 | NsPGSQVASNPR |
| 694 | Q9H0D6 | MQNNSSPSIsPNTSFTSDGSPSPLGGIK |
| 695 | Q9H0D6 | MQNNSsPSISPNTSFTSDGSPSPLGGIK |
| 696 | Q9H063 | LSKsQGGEEEGPLSDK |
| 697 | Q9H019 | NAsVPNLR |
| 698 | Q9GZY8 | ERsMsENAVR |
| 699 | Q9GZR7 | AQAVsEEEEEEEGK |
| 700 | Q9GZR7 | sPGKAEAESDALPDDTVIESEALPSDIAAEAR |
| 701 | Q9GZR7 | SPGKAEAEsDALPDDTVIESEALPSDIAAEAR |
| 702 | Q9GZR7 | AQAVSEEEEEEEGKSSsPK |
| 703 | Q9C0E2 | HQQQLLAsPGSSTVDNK |
| 704 | Q9C0C9 | LIHGEDsDsEGEEEGR |
| 705 | Q9C0C9 | LIHGEDsDsEGEEEGR |
| 706 | Q9C0C2 | GWsQEGPVK |
| 707 | Q9C0C2 | sQEADVQDWEFR |
| 708 | Q9C0C2 | HNGSLsPGLEAR |
| 709 | Q9C0C2 | HNGsLSPGLEAR |
| 710 | Q9C0C2 | REsAAsGLGGLLEEEGAGAGAAQEEVLEPGR |
| 711 | Q9C0C2 | REsAAsGLGGLLEEEGAGAGAAQEEVLEPGR |
| 712 | Q9C0C2 | ASRVPssDEEVVEEPQSR |
| 713 | Q9C0C2 | ASRVPssDEEVVEEPQSR |
| 714 | Q9C0C2 | NRsAEEGELAESK |
| 715 | Q9C0C2 | AsPEPPGPESSSR |
| 716 | Q9C0C2 | DsLGTYSSR |
| 717 | Q9C0C2 | EAAFsPGQQDWSR |
| 718 | Q9C0C2 | RFsEGVLQSPSQDQEK |
| 719 | Q9C0C2 | YESQEPLAGQEsPLPLATR |
| 720 | Q9C0C2 | VSGAGFsPSSK |
| 721 | Q9C0C2 | GSGGLFsPSTAHVPDGALGQR |
| 722 | Q9C0C2 | LDsPPPSPITEASEAAEAAEAGNLAVSSR |
| 723 | Q9C0C2 | LDsPPPsPITEASEAAEAAEAGNLAVSSR |
| 724 | Q9C0C2 | DTQsPSTCSEGLLGWSQK |
| 725 | Q9C0B5 | GVGsPEPGPTAPYLGR |
| 726 | Q9C0B5 | GDsLKEPTSIAESSR |
| 727 | Q9C0B5 | SLGSAsPGPGQPPLSsPTR |
| 728 | Q9C0B5 | SLGSASPGPGQPPLSsPTR |
| 729 | Q9C086 | AWLDEDSNLsPsPLR |

| | | |
|---|---|---|
| 730 | Q9C086 | AWLDEDsNLSPsPLR |
| 731 | Q9C086 | AWLDEDSNLsPsPLR |
| 732 | Q9BZQ8 | RAsAILPGVLGSETLSNEVFQEsEEEKQPEVPSSLAK |
| 733 | Q9BZI7 | KLDKENLsDER |
| 734 | Q9BZI7 | FLESYAtDNEK |
| 735 | Q9BZF1 | GYSSPEPDIQDssGsEAQSVKPSTR |
| 736 | Q9BZF1 | GYSSPEPDIQDssGsEAQSVKPSTR |
| 737 | Q9BZF1 | GYSSPEPDIQDssGsEAQSVKPSTR |
| 738 | Q9BZE4 | TAAGEYDSVsEsEDEEMLEIR |
| 739 | Q9BZE4 | TAAGEYDSVsEsEDEEMLEIR |
| 740 | Q9BZE4 | TAAGEYDsVsESEDEEMLEIR |
| 741 | Q9BZC7 | VSEEDQsLENsEADVK |
| 742 | Q9BZC7 | VSEEDQsLENsEADVK |
| 743 | Q9BZ23 | RAssASVPAVGASAEGTR |
| 744 | Q9BZ23 | RAssASVPAVGASAEGTR |
| 745 | Q9BYX2 | AVsEGCAsEDEVEGEA |
| 746 | Q9BYX2 | AVSEGCAsEDEVEGEA |
| 747 | Q9BYW2 | SSQSEGIFLGsEsDEDSVR |
| 748 | Q9BYW2 | SSQSEGIFLGsEsDEDSVR |
| 749 | Q9BYG3 | TVDSQGPtPVCtPTFLER |
| 750 | Q9BYG3 | TVDSQGPtPVCtPTFLER |
| 751 | Q9BY89 | sPFQPGVLGSR |
| 752 | Q9BY89 | sPKsPFQPGVLGSR |
| 753 | Q9BY89 | KRQsLYENQV |
| 754 | Q9BY44 | NTVSQsISGDPEIDKK |
| 755 | Q9BXP5 | ERFsPPRHELsPPQK |
| 756 | Q9BXP5 | GDsDDEYDR |
| 757 | Q9BXB4 | SFSLASSSNsPISQR |
| 758 | Q9BX95 | NsLTGEEGQLAR |
| 759 | Q9BWU0 | NWEDEDFYDsDDDTFLDR |
| 760 | Q9BWH2 | KsNQIPTEVR |
| 761 | Q9BWG6 | DENVEFDsDEEEPPDLPLD |
| 762 | Q9BWF3 | LHVGNIsPTCTNK |
| 763 | Q9BW85 | LLEDsDsEDEAAPSPLQPALRPNPTAILDEAPKPK |
| 764 | Q9BW85 | LLEDsDsEDEAAPSPLQPALRPNPTAILDEAPKPK |
| 765 | Q9BW71 | EEsEEsEAEPVQR |
| 766 | Q9BW71 | EEsEESEAEPVQR |
| 767 | Q9BW71 | RPPTPCsDPER |
| 768 | Q9BW71 | RPPtPCsDPER |
| 769 | Q9BW71 | SLKEsEQEsEEEILAQK |
| 770 | Q9BW71 | ESEQEsEEEILAQK |

| | | |
|---|---|---|
| 771 | Q9BVV8 | RYGLLANTEDPTEMAsLDsDEETVFESR |
| 772 | Q9BVV8 | RYGLLANTEDPTEMAsLDsDEETVFESR |
| 773 | Q9BVS4 | EGSEFSFsDGEVAEK |
| 774 | Q9BVS4 | SSGDPEQIKEDsLsEESADAR |
| 775 | Q9BVS4 | SSGDPEQIKEDsLsEESADAR |
| 776 | Q9BVS4 | EGsEFSFsDGEVAEK |
| 777 | Q9BVS4 | EGSEFsFsDGEVAEK |
| 778 | Q9BVJ6 | DYLLsEsEDEGDNDGER |
| 779 | Q9BVJ6 | DYLLsEsEDEGDNDGER |
| 780 | Q9BVJ6 | EAATQEDPEQLPELEAHGVsEsEGEERPVAEEEILLR |
| 781 | Q9BVJ6 | EAATQEDPEQLPELEAHGVsEsEGEERPVAEEEILLR |
| 782 | Q9BVG9 | DAGGPRPEsPVPAGR |
| 783 | Q9BVG4 | GADsGEEKEEGINR |
| 784 | Q9BVC5 | KSPSGPVKsPPLsPVGTTPVK |
| 785 | Q9BVC5 | KSPSGPVKsPPLSPVGttPVK |
| 786 | Q9BVC5 | KsPSGPVKSPPLsPVGTTPVK |
| 787 | Q9BVC5 | KSPSGPVKsPPLSPVGttPVK |
| 788 | Q9BUZ4 | GsLDESSLGFGYPK |
| 789 | Q9BUR4 | EGDPVSLSTPLETEFGsPSELSPR |
| 790 | Q9BUR4 | EGDPVSLSTPLETEFGsPSELsPR |
| 791 | Q9BUR4 | GDPPRLsPDPVAGSAVSQELR |
| 792 | Q9BUR4 | IEEQELSENTSLPAEEANGSLsEEEANGPELGSGK |
| 793 | Q9BUQ8 | KRSsLsPGR |
| 794 | Q9BUQ8 | KRSsLsPGR |
| 795 | Q9BUL5 | GESAADsDGWDSAPSDLR |
| 796 | Q9BUH6 | LAAAEETAVsPR |
| 797 | Q9BUA3 | AEsPSPAPPPGLR |
| 798 | Q9BUA3 | NLDPDPEPPsPDsPTETFAAPAEVR |
| 799 | Q9BUA3 | NLDPDPEPPsPDsPTETFAAPAEVR |
| 800 | Q9BU76 | RPAEATSsPTsPERPR |
| 801 | Q9BU76 | RPAEATSsPTsPERPR |
| 802 | Q9BTU6 | VAAAAGSGPsPPGsPGHDR |
| 803 | Q9BTU6 | VAAAAGSGPsPPGSPGHDR |
| 804 | Q9BTU6 | MDETsPLVsPER |
| 805 | Q9BTU6 | MDETsPLVsPER |
| 806 | Q9BTK6 | DLFSLDSEDPSPAsPPLR |
| 807 | Q9BTE3 | VsPSTSYTPSR |
| 808 | Q9BTC0 | sPPEGDTTLFLSR |
| 809 | Q9BTC0 | RNsVERPAEPVAGAATPSLVEQQK |
| 810 | Q9BTC0 | YPLCSADAAVSTtPPGsPPPPPLPEPPVLK |
| 811 | Q9BTC0 | QEAIPDLEDsPPVsDSEEQQESAR |

| | | |
|---|---|---|
| 812 | Q9BTC0 | YPLCSADAAVSTtPPGsPPPPPPLPEPPVLK |
| 813 | Q9BTC0 | QEAIPDLEDsPPVsDSEEQQESAR |
| 814 | Q9BTC0 | GGDDHDDtsDsDSDGLTLK |
| 815 | Q9BTC0 | GGDDHDDtsDsDSDGLTLK |
| 816 | Q9BTC0 | GGDDHDDtsDsDSDGLTLK |
| 817 | Q9BTA9 | DAGDPsPPNK |
| 818 | Q9BTA9 | sPsPGPNHTSNSSNASNATVVPQNSSAR |
| 819 | Q9BTA9 | sPsPGPNHTSNSSNASNATVVPQNSSAR |
| 820 | Q9BRT9 | MTEEVDFLGQDsDGGsEEVVLTPAELIER |
| 821 | Q9BRT9 | MTEEVDFLGQDsDGGsEEVVLTPAELIER |
| 822 | Q9BRK4 | AsPDSSSCGERsPPPPPPPPSDEALLHCVLEGK |
| 823 | Q9BRJ6 | ELDEEGsDPPLPGR |
| 824 | Q9BRD0 | HGtPDPSPR |
| 825 | Q9BRD0 | HGtPDPsPR |
| 826 | Q9BRD0 | DRHDtPDPsPR |
| 827 | Q9BRD0 | DRHDtPDPsPR |
| 828 | Q9BRD0 | ARHDsPDPsPPR |
| 829 | Q9BRD0 | ARHDsPDPsPPR |
| 830 | Q9BRD0 | ARHDtPDPsPLR |
| 831 | Q9BRD0 | ARHDtPDPsPLR |
| 832 | Q9BRD0 | HDsPDPsPPRR |
| 833 | Q9BRD0 | HDsPDPsPPRR |
| 834 | Q9BRD0 | HDsPDLAPNVTYSLPR |
| 835 | Q9BR39 | ETPRPEGGSPsPAGtPPQPK |
| 836 | Q9BR39 | ETPRPEGGSPsPAGtPPQPK |
| 837 | Q9BQQ3 | KPPGTPPPsALPLGAPPPDALPPGPtPEDsPSLETGSR |
| 838 | Q9BQQ3 | KPPGTPPPsALPLGAPPPDALPPGPtPEDsPSLETGSR |
| 839 | Q9BQQ3 | KPPGtPPPSALPLGAPPPDALPPGPTPEDSPSLETGSR |
| 840 | Q9BQG0 | EIPSATQsPISK |
| 841 | Q9BQE9 | TDDSQPPTLGQEILEEPsLPSSEVADEPPTLTK |
| 842 | Q9BQE3 | DYEEVGADsADGEDEGEEY |
| 843 | Q9BQA1 | KEtPPPLVPPAAR |
| 844 | Q9BPX3 | LNLAQFLNEDLs |
| 845 | Q9BPX3 | CQTAEADsEsDHEVPEPESEMK |
| 846 | Q9BPX3 | CQTAEADsEsDHEVPEPESEMK |
| 847 | Q9BPX3 | TLHCEGTEINsDDEQESK |
| 848 | Q99959 | LEIsPDSsPER |
| 849 | Q99959 | LEIsPDSsPER |
| 850 | Q99958 | VETLSPESALQGsPR |
| 851 | Q99958 | VETLsPESALQGsPR |
| 852 | Q99871 | GGDDYsEDEGDSSVSR |

| | | |
|---|---|---|
| 853 | Q99856 | AAAAGLGHPAsPGGSEDGPPGsEEEDAAR |
| 854 | Q99856 | AAAAGLGHPAsPGGSEDGPPGsEEEDAAR |
| 855 | Q99856 | AAAAGLGHPAsPGGsEDGPPGsEEEDAAR |
| 856 | Q99733 | ADHSFSDGVPsDSVEAAK |
| 857 | Q99733 | ADHsFSDGVPSDSVEAAK |
| 858 | Q99733 | ADHSFsDGVPSDSVEAAK |
| 859 | Q99733 | EFITGDVEPTDAESEWHsENEEEEK |
| 860 | Q99717 | VLTQMGSPLNPISsVs |
| 861 | Q99717 | VLTQMGSPLNPISsVs |
| 862 | Q99638 | SLFFGSILAPVRsPQGPSPVLAEDsEGEG |
| 863 | Q99638 | SLFFGSILAPVRsPQGPsPVLAEDsEGEG |
| 864 | Q99638 | SLFFGSILAPVRsPQGPSPVLAEDsEGEG |
| 865 | Q99613 | QPLLLsEDEEDTK |
| 866 | Q99590 | AETASQSQRsPIsDNsGCDAPGNSNPSLSVPSSAESEK |
| 867 | Q99590 | FHsPSTTWSPNK |
| 868 | Q99590 | TEELIEsPKLESSEGEIIQTVDR |
| 869 | Q99590 | TEELIEsPKLEsSEGEIIQTVDR |
| 870 | Q99590 | HFSEDNNEMIPMECDsFCSDQNEsEVEPSVNADLK |
| 871 | Q99590 | HFSEDNNEMIPMECDsFCsDQNESEVEPSVNADLK |
| 872 | Q99549 | GAEAFGDsEEDGEDVFEVEK |
| 873 | Q99543 | NAsAsFQELEDK |
| 874 | Q99543 | NAsASFQELEDK |
| 875 | Q99543 | ELsEEsEDEELQLEEFPMLK |
| 876 | Q99543 | ELsEEsEDEELQLEEFPMLK |
| 877 | Q99501 | VSSPsPELGTTPASIFR |
| 878 | Q99460 | TSSAFVGKtPEAsPEPK |
| 879 | Q99460 | TSSAFVGKtPEAsPEPK |
| 880 | Q99459 | GGLNtPLHESDFSGVtPQR |
| 881 | Q99459 | GGLNtPLHESDFSGVtPQR |
| 882 | Q99442 | EELEQQtDGDCEEDEEEENDGETPK |
| 883 | Q96TA1 | GLLAQGLRPEsPPPAGPLLNGAPAGESPQPK |
| 884 | Q96TA1 | GLLAQGLRPEsPPPAGPLLNGAPAGEsPQPK |
| 885 | Q96TA1 | QVVSVVQDEEVGLPFEASPEsPPPASPDGVTEIR |
| 886 | Q96TA1 | QVVSVVQDEEVGLPFEASPESPPPAsPDGVTEIR |
| 887 | Q96TA1 | QVVsVVQDEEVGLPFEASPESPPPASPDGVtEIR |
| 888 | Q96TA1 | AAPEASsPPAsPLQHLLPGK |
| 889 | Q96TA1 | AAPEASsPPASPLQHLLPGK |
| 890 | Q96TA1 | QVVSVVQDEEVGLPFEAsPESPPPAsPDGVTEIR |
| 891 | Q96T88 | DSELsDTDSGCCLGQSESDK |
| 892 | Q96T58 | sQsPVHLR |
| 893 | Q96T58 | sQsPVHLR |

| | | |
|---|---|---|
| 894 | Q96T58 | HGsFHEDEDPIGsPR |
| 895 | Q96T58 | HGSFHEDEDPIGsPR |
| 896 | Q96T58 | RPQsPGASPsQAERLPsDSER |
| 897 | Q96T58 | RPQsPGAsPSQAER |
| 898 | Q96T58 | DLEPGEVPsDSDEDGEHKsHsPR |
| 899 | Q96T58 | DSELKTPPsVGPPSVTVVTLESAPSALEK |
| 900 | Q96T37 | sLSPGGAALGYR |
| 901 | Q96T37 | SLsPGGAALGYR |
| 902 | Q96T37 | HCAPsPDRsPELSSSR |
| 903 | Q96T37 | HCAPsPDRSPELSSSR |
| 904 | Q96T37 | sRsPLDKDTYPPSASVVGASVGGHR |
| 905 | Q96T37 | sRsPLDKDTYPPSASVVGASVGGHR |
| 906 | Q96T23 | DAQRLsPIPEEVPK |
| 907 | Q96T23 | IEsDEEEDFENVGK |
| 908 | Q96T23 | IEtDEEESCDNAHGDANQPAR |
| 909 | Q96T23 | IEtDEEEsCDNAHGDANQPAR |
| 910 | Q96ST3 | GDLsDVEEEEEEEMDVDEATGAVK |
| 911 | Q96ST2 | MDsDEDEKEGEEEK |
| 912 | Q96ST2 | EAEDsDSDDNIKR |
| 913 | Q96ST2 | EAEDsDsDDNIKR |
| 914 | Q96ST2 | AAVLsDsEDEEK |
| 915 | Q96ST2 | AAVLsDsEDEEK |
| 916 | Q96ST2 | EKTIAsDsEEEAGK |
| 917 | Q96ST2 | EKTIAsDsEEEAGK |
| 918 | Q96ST2 | VVsDADDsDSDAVSDK |
| 919 | Q96ST2 | VVsDADDsDSDAVSDK |
| 920 | Q96ST2 | GPAsDsETEDASR |
| 921 | Q96ST2 | GPAsDsETEDASR |
| 922 | Q96ST2 | IsDSESEDPPRHQAsDsENEELPKPR |
| 923 | Q96ST2 | ISDsESEDPPR |
| 924 | Q96ST2 | GHHVTDSENDEPLNLNAsDsESEELHR |
| 925 | Q96ST2 | GHHVTDSENDEPLNLNAsDsESEELHR |
| 926 | Q96SB4 | GSAPHSESDLPEQEEEILGsDDDEQEDPNDYCK |
| 927 | Q96S82 | DMPGGFLFEGLsDDEDDFHPNTR |
| 928 | Q96S66 | ESSTESSQSAKPVSGQDTSGNTEGsPAAEK |
| 929 | Q96S55 | AKGPsPPGAK |
| 930 | Q96S55 | RPAAAAAAGSAsPR |
| 931 | Q96RU3 | TVsDNSLSNSR |
| 932 | Q96RU3 | TVsDNsLSNSR |
| 933 | Q96RT1 | HIVNHDDVFEESEELssDEEMK |
| 934 | Q96RT1 | HIVNHDDVFEESEELssDEEMK |

| | | |
|---|---|---|
| 935 | Q96RS0 | GIGLDEsELDsEAELMR |
| 936 | Q96RS0 | GIGLDEsELDsEAELMR |
| 937 | Q96RL1 | LEDAFIVIsDsDGEEPKEENGLQK |
| 938 | Q96RL1 | LEDAFIVIsDsDGEEPKEENGLQK |
| 939 | Q96QU8 | HSVTAAtPPPSPtSGESGDLLSNLLQSPSSAK |
| 940 | Q96QU8 | HSVTAATPPPsPTSGESGDLLSNLLQSPSSAK |
| 941 | Q96QT6 | TTsPSSDTDLLDR |
| 942 | Q96QT6 | TTsPSsDTDLLDR |
| 943 | Q96QR8 | GGGSGGGEEsEGEEVDED |
| 944 | Q96QR8 | GGGsGGGEEsEGEEVDED |
| 945 | Q96QR8 | ADGDsGsERGGGGGPCGFQPASR |
| 946 | Q96QR8 | ADGDsGsERGGGGGPCGFQPASR |
| 947 | Q96Q15 | NLATsADtPPSTVPGTGK |
| 948 | Q96Q15 | NLATsADtPPSTVPGTGK |
| 949 | Q96PU5 | sLSSPTVTLSAPLEGAK |
| 950 | Q96PU5 | DTLSNPQsPQPsPYNSPKPQHK |
| 951 | Q96PU5 | DTLSNPQsPQPsPYNSPKPQHK |
| 952 | Q96PU5 | sLSsPTVTLSAPLEGAK |
| 953 | Q96PU5 | DTLSNPQsPQPsPYNsPKPQHK |
| 954 | Q96PU4 | RPIsDDDCPSASK |
| 955 | Q96PN7 | SNSIDGSNVTVtPGPGEQTVDVEPR |
| 956 | Q96PK6 | QPtPPFFGR |
| 957 | Q96PK6 | LSEsQLSFR |
| 958 | Q96NT5 | ADPHLEFQQFPQsP |
| 959 | Q96NA2 | AEssEDETSSPAPSK |
| 960 | Q96NA2 | AESsEDETSSPAPSK |
| 961 | Q96N67 | SPsGSAFGSQENLR |
| 962 | Q96N64 | sPEAVGPELEAEEK |
| 963 | Q96N64 | VAESAAAAPQGGPELPPsPAsPPEQPPAPEER |
| 964 | Q96N64 | VAESAAAAPQGGPELPPsPAsPPEQPPAPEER |
| 965 | Q96MW1 | AALLAQYADVtDEEDEADEKDDSGATTMNIGSDK |
| 966 | Q96MU7 | GIsPIVFDR |
| 967 | Q96MH2 | TSGAPGsPQtPPER |
| 968 | Q96MH2 | TSGAPGsPQTPPER |
| 969 | Q96MH2 | MEsHsEDEDLAGAVGGLGWNSR |
| 970 | Q96MH2 | MEsHsEDEDLAGAVGGLGWNSR |
| 971 | Q96MH2 | TQsPGGCSAEAVLAR |
| 972 | Q96LR5 | VDDSPSTSGGSsDGDQR |
| 973 | Q96LD4 | GGIPAsPIDPFQSR |
| 974 | Q96L91 | KAsISLtDDEVDDEEETIEEEEANEGVVDHQTELSNLAK |
| 975 | Q96L91 | KAsISLtDDEVDDEEETIEEEEANEGVVDHQTELSNLAK |

| | | |
|---|---|---|
| 976 | Q96KR1 | DsDGVDGFEAEGK |
| 977 | Q96KC8 | DFDIAEQNEssDEESLR |
| 978 | Q96KC8 | DFDIAEQNESsDEEsLRK |
| 979 | Q96K76 | ETWDTAEEDsGtDSEYDESGK |
| 980 | Q96K21 | WsPPQNYK |
| 981 | Q96K21 | LPDsDDDEDEETAIQR |
| 982 | Q96JY6 | AGsPFsPPPSSSSLTGEAAISR |
| 983 | Q96JY6 | AGsPFsPPPSSSSLTGEAAISR |
| 984 | Q96JP2 | AVPsPPPPPIVK |
| 985 | Q96JM3 | GGsPDLWK |
| 986 | Q96JM3 | KPGPPLsPEIRsPAGSPELR |
| 987 | Q96JM3 | KPGPPLsPEIRsPAGsPELR |
| 988 | Q96JM3 | KPGPPLsPEIR |
| 989 | Q96JM3 | LAPVPsPEPQKPAPVsPESVK |
| 990 | Q96JM3 | LAPVPsPEPQKPAPVsPESVK |
| 991 | Q96JM3 | SSSVSPSSWKsPPAsPESWK |
| 992 | Q96JM3 | SSSVSPSSWKSPPAsPEsWK |
| 993 | Q96JM3 | DNQESsDAELSSSEYIK |
| 994 | Q96JM3 | TAPTLsPEHWK |
| 995 | Q96JM3 | DNQESsDAELsSSEYIK |
| 996 | Q96JM3 | KPSPSESPEPWKPFPAVsPEPR |
| 997 | Q96JM3 | KPsPSESPEPWKPFPAVsPEPR |
| 998 | Q96JM3 | GQEsSsDQEQVDVESIDFSK |
| 999 | Q96JM3 | GQEssSDQEQVDVESIDFSK |
| 1000 | Q96JG6 | SAYQEYDSDsDVPEELKR |
| 1001 | Q96JG6 | SAYQEYDsDsDVPEELK |
| 1002 | Q96JG6 | FMEQSRsPSVsPSK |
| 1003 | Q96JG6 | FMEQSRSPSVsPSKQPVSTSSK |
| 1004 | Q96JC9 | DNPsPEPQLDDIKR |
| 1005 | Q96I25 | RPDPDsDEDEDYER |
| 1006 | Q96HR8 | NDQEPPPEALDFsDDEKEK |
| 1007 | Q96HH9 | SVCGHLENTSVGNsPNPSSAENSFR |
| 1008 | Q96HH9 | SVCGHLENTsVGNsPNPSSAENSFR |
| 1009 | Q96GX9 | DISGPsPSK |
| 1010 | Q96GM8 | AADsDDGAVSAPAASDGGVSK |
| 1011 | Q96G74 | AsPPPQGPLPGPPGALHR |
| 1012 | Q96G74 | QAPGVGAVGGGsPEREEVGAGYNsEDEYEAAAAR |
| 1013 | Q96G74 | QAPGVGAVGGGsPEREEVGAGyNSEDEYEAAAAR |
| 1014 | Q96G74 | QAPGVGAVGGGsPEREEVGAGYNsEDEYEAAAAR |
| 1015 | Q96FS4 | AHsHEEASRPAATSTR |
| 1016 | Q96FS4 | SGsDAGEARPPTPASPR |

| | | |
|---|---|---|
| 1017 | Q96FS4 | TEFLHSQNSLsPR |
| 1018 | Q96FS4 | TEFLHSQNsLsPR |
| 1019 | Q96FF9 | THSVPAtPTSTPVPNPEAESSSK |
| 1020 | Q96F86 | SQDVAVsPQQQQCSK |
| 1021 | Q96F63 | LLQQQEEEEACLEEEEEEEDsDEEDQR |
| 1022 | Q96EZ8 | GDQVLNFsDAEDLIDDSK |
| 1023 | Q96EV8 | DISEGGESPVVQsDEEEVQVDTALATSHTDR |
| 1024 | Q96EV8 | DISEGGEsPVVQsDEEEVQVDTALATSHTDR |
| 1025 | Q96EV2 | DIKEEsDEEEEDDEESGR |
| 1026 | Q96EV2 | TNsGGGDGPHISSK |
| 1027 | Q96ET8 | MLQQDsNDDTEDVSLFDAEEETTNRPR |
| 1028 | Q96ET8 | MLQQDsNDDTEDVsLFDAEEETTNRPR |
| 1029 | Q96EQ0 | SFsSSAEEHS |
| 1030 | Q96EN8 | SLsPQEDALTGSR |
| 1031 | Q96EN8 | sLSPQEDALTGSR |
| 1032 | Q96EB6 | DGPGLERsPGEPGGAAPER |
| 1033 | Q96EB6 | ADEAALALQPGGsPSAAGADR |
| 1034 | Q96EB6 | ADEAALALQPGGsPsAAGADREAASSPAGEPLR |
| 1035 | Q96EB6 | ADEAALALQPGGsPSAAGADREAASsPAGEPLR |
| 1036 | Q96DX7 | AEGDEEGPsGAsEEEDT |
| 1037 | Q96DX7 | AEGDEEGPSGAsEEEDT |
| 1038 | Q96DF8 | GLsPAMsPALQR |
| 1039 | Q96DF8 | GLsPAMsPALQR |
| 1040 | Q96D71 | SSsLDMNR |
| 1041 | Q96D71 | RQsSSYDDPWK |
| 1042 | Q96D71 | RQssSYDDPWK |
| 1043 | Q96D46 | DSAIPVEsDTDDEGAPR |
| 1044 | Q96D46 | DSAIPVESDtDDEGAPR |
| 1045 | Q96CW6 | EFGYDSPHDLDsD |
| 1046 | Q96CW6 | EFGYDsPHDLDsD |
| 1047 | Q96CW1 | EEQSQITSQVtGQIGWR |
| 1048 | Q96CV9 | LNSSGSSEDsFVEIR |
| 1049 | Q96C57 | EAAVSASDILQESAIHsPGTVEK |
| 1050 | Q96C19 | ADLNQGIGEPQsPSR |
| 1051 | Q96BT3 | AAsPESASSTPESLQAR |
| 1052 | Q96BD0 | ASPGTPLSPGsLR |
| 1053 | Q96BD0 | ASPGTPLsPGSLR |
| 1054 | Q96BD0 | ASPGtPLSPGsLR |
| 1055 | Q96BD0 | ASPGTPLsPGsLRsAAHSPLDTSK |
| 1056 | Q96BD0 | SAAHsPLDTSK |
| 1057 | Q96B97 | sIEVENDFLPVEK |

| | | |
|---|---|---|
| 1058 | Q96B36 | LNtSDFQK |
| 1059 | Q96B36 | SSDEENGPPSsPDLDR |
| 1060 | Q96B36 | TEARssDEENGPPSsPDLDR |
| 1061 | Q96B21 | LNSDDTYQTALLSGsDEE |
| 1062 | Q96B21 | LNSDDTYQTALLsGsDEE |
| 1063 | Q96AY2 | LLssEsEDEEEFIPLAQR |
| 1064 | Q96AY2 | LLssEsEDEEEFIPLAQR |
| 1065 | Q96AY2 | LLssEsEDEEEFIPLAQR |
| 1066 | Q96AY2 | SSPSLDsGDsDSEELPTFAFLK |
| 1067 | Q96AY2 | SSPSLDsGDsDSEELPTFAFLK |
| 1068 | Q96AV8 | HGsFNTVQASER |
| 1069 | Q96AT1 | IQPQPPDEDGDHsDKEDEQPQVVVLK |
| 1070 | Q96AT1 | NSSLLsFDNEDENE |
| 1071 | Q96A57 | LSsTDDGYIDLQFK |
| 1072 | Q96A57 | LssTDDGYIDLQFK |
| 1073 | Q96A49 | EQDLPLAEAVRPKtPPVVIK |
| 1074 | Q96A49 | TQEDEEEISTsPGVSEFVSDAFDACNLNQEDLR |
| 1075 | Q969V6 | FGSTGStPPVsPTPSER |
| 1076 | Q969V6 | FGSTGStPPVsPTPSER |
| 1077 | Q969H6 | SCLLEEEEEsGEEAAEAME |
| 1078 | Q969H4 | sPSLSLAPLsPR |
| 1079 | Q969H4 | sPSLSLAPLsPR |
| 1080 | Q969G5 | APEPLGPADQSELGPEQLEAEVGEssDEEPVESR |
| 1081 | Q969G5 | APEPLGPADQSELGPEQLEAEVGEssDEEPVESR |
| 1082 | Q92974 | ERQELGsPEER |
| 1083 | Q92974 | LQDSSDPDTGSEEEGSSRLsPPHsPR |
| 1084 | Q92974 | LQDSSDPDTGSEEEGSSRLSPPHsPR |
| 1085 | Q92974 | SEsLESPR |
| 1086 | Q92945 | VQIsPDSGGLPER |
| 1087 | Q92922 | KHsPsPPPPTPTESR |
| 1088 | Q92922 | KHsPsPPPPTPTESR |
| 1089 | Q92917 | ENQVVELHYDAICQYMGPsDTDDD |
| 1090 | Q92900 | SQIDVALsQDSTYQGER |
| 1091 | Q92900 | SQIDVALsQDsTYQGER |
| 1092 | Q92888 | DGDGVPGGGPLsPAR |
| 1093 | Q92882 | TLSNAEDYLDDEDsD |
| 1094 | Q92797 | SPQTLAPVGEDAMKtPsPAAEDAR |
| 1095 | Q92797 | SPQTLAPVGEDAMKtPsPAAEDAR |
| 1096 | Q92769 | IACDEEFsDsEDEGEGGR |
| 1097 | Q92769 | IACDEEFsDsEDEGEGGR |
| 1098 | Q92766 | ANsGGVDLDSSGEFASIEK |

| | | |
|---|---|---|
| 1099 | Q92766 | ANSGGVDLDssGEFASIEK |
| 1100 | Q92766 | ANSGGVDLDssGEFASIEK |
| 1101 | Q92733 | IAAPELHKGDsDsEEDEPTK |
| 1102 | Q92733 | IAAPELHKGDsDsEEDEPTK |
| 1103 | Q92733 | QITQEEDDsDEEVAPENFFSLPEK |
| 1104 | Q92621 | LQDsFAsETNLDFR |
| 1105 | Q92621 | LQDsFAsETNLDFR |
| 1106 | Q92619 | AGsPSPQPSGELPR |
| 1107 | Q92619 | AGsPsPQPSGELPR |
| 1108 | Q92615 | EPSVPASCAVSATYERsPsPAHLPDDPK |
| 1109 | Q92615 | EPSVPASCAVSATYERsPsPAHLPDDPK |
| 1110 | Q92614 | LEGDsDVDsELEDR |
| 1111 | Q92614 | LEGDsDVDsELEDR |
| 1112 | Q92614 | AAsDDGsLKSSsPTSYWK |
| 1113 | Q92614 | YSHSYLsDsDTEAK |
| 1114 | Q92614 | YSHSYLsDSDTEAK |
| 1115 | Q92610 | SIKPSDsPR |
| 1116 | Q92609 | NISSSPsVEsLPGGR |
| 1117 | Q92609 | NISSSPsVEsLPGGR |
| 1118 | Q92597 | SRTAsGSSVTSLDGTR |
| 1119 | Q92597 | TASGSsVTsLDGTR |
| 1120 | Q92597 | SRTAsGSSVTsLDGTR |
| 1121 | Q92597 | SRtASGsSVTSLDGTR |
| 1122 | Q92597 | sRtAsGSSVTSLDGTR |
| 1123 | Q92539 | EEQAASAAAEDTCDVGVssDDDKGAQAAR |
| 1124 | Q92539 | EEQAASAAAEDTCDVGVssDDDKGAQAAR |
| 1125 | Q92538 | GYTsDSEVYTDHGRPGK |
| 1126 | Q92538 | SGCsDLEEAVDSGADKK |
| 1127 | Q92538 | ADAPDAGAQsDSELPSYHQNDVSLDR |
| 1128 | Q92538 | AASSSSPGsPVASSPSR |
| 1129 | Q92538 | AASSSSPGsPVASsPSR |
| 1130 | Q92538 | AASSsSPGSPVASsPSR |
| 1131 | Q92522 | AGGSAALsPSK |
| 1132 | Q92466 | sRsPLELEPEAK |
| 1133 | Q92466 | SRsPLELEPEAK |
| 1134 | Q8WZ73 | VPAEDETQsIDsEDSFVPGR |
| 1135 | Q8WZ73 | VPAEDETQSIDsEDSFVPGR |
| 1136 | Q8WYQ5 | EQSSDLTPsGDVsPVKPLSR |
| 1137 | Q8WYQ5 | EQSSDLTPSGDVsPVKPLSR |
| 1138 | Q8WYQ5 | YGGDsDHPsDGETSVQPMMTK |
| 1139 | Q8WYQ5 | YGGDsDHPsDGETSVQPMMTK |

| | | |
|---|---|---|
| 1140 | Q8WYP5 | EVsPSDVR |
| 1141 | Q8WYA6 | EYAENIGDGRsPEFR |
| 1142 | Q8WY36 | TADGRVsPAGGTLDDKPK |
| 1143 | Q8WXI9 | GRLtPSPDIIVLsDNEASSPR |
| 1144 | Q8WXI9 | GRLtPSPDIIVLSDNEASsPR |
| 1145 | Q8WXI9 | GRLtPsPDIIVLSDNEASSPR |
| 1146 | Q8WXI9 | GRLtPSPDIIVLSDNEASsPR |
| 1147 | Q8WXG6 | LAsDSDAESDSR |
| 1148 | Q8WXG6 | LASDsDAESDSR |
| 1149 | Q8WXG6 | KRsPTESVNTPVGK |
| 1150 | Q8WXE0 | sGEQIFTQDVRPEQLLEGK |
| 1151 | Q8WXE0 | SQsFALR |
| 1152 | Q8WXE0 | SHSLSRPGPtEGDAEGEAEGPVGSTLGSYAtLTR |
| 1153 | Q8WX93 | sRSRDsGDENEPIQER |
| 1154 | Q8WX93 | sRDsGDENEPIQER |
| 1155 | Q8WX93 | DsGDENEPIQER |
| 1156 | Q8WX93 | IAsDEEIQGTK |
| 1157 | Q8WX93 | SAPPsPPFPPPPAFPELAACtPPAsPEPMSALASR |
| 1158 | Q8WX93 | sAPPSPPFPPPPAFPELAACtPPAsPEPMSALASR |
| 1159 | Q8WX92 | KPsPAQAAETPALELPLPSVPAPAPL |
| 1160 | Q8WWY3 | QSVVYGGKstIR |
| 1161 | Q8WWY3 | QSVVYGGKstIR |
| 1162 | Q8WWY3 | SSGTASsVAFtPLQGLEIVNPQAAEK |
| 1163 | Q8WWY3 | SSGTASSVAFtPLQGLEIVNPQAAEK |
| 1164 | Q8WWY3 | SSGTAsSVAFtPLQGLEIVNPQAAEK |
| 1165 | Q8WWQ0 | KVLsDsEDEEKDADVPGTSTR |
| 1166 | Q8WWQ0 | KVLsDsEDEEKDADVPGTSTR |
| 1167 | Q8WWQ0 | GsISSTSEVHSPPNVGLR |
| 1168 | Q8WWQ0 | TAFYNEDDsEEEQR |
| 1169 | Q8WWQ0 | AQsYDIQAWK |
| 1170 | Q8WWM7 | EKEVDGLLTSEPMGsPVSSK |
| 1171 | Q8WWM7 | EsPSLASR |
| 1172 | Q8WWL2 | DRsFSEHDLAQLR |
| 1173 | Q8WWI1 | GEsLDNLDSPR |
| 1174 | Q8WWI1 | EVAATEEDVTRLPsPTsPFSSLSQDQAATSK |
| 1175 | Q8WWI1 | EVAATEEDVTRLPsPTSPFSSLSQDQAATSK |
| 1176 | Q8WWI1 | TSTTGVATTQsPTPR |
| 1177 | Q8WWI1 | TPNNVVStPAPsPDASQLASSLSSQK |
| 1178 | Q8WWI1 | TPNNVVSTPAPsPDASQLASSLSSQK |
| 1179 | Q8WWI1 | VTTEIQLPSQSPVEEQsPASLSSLR |
| 1180 | Q8WWI1 | VTTEIQLPSQsPVEEQSPASLSSLR |

| | | |
|---|---|---|
| 1181 | Q8WWI1 | MYsFDDVLEEGK |
| 1182 | Q8WWI1 | ATLSSTSGLDLMSESGEGEIsPQR |
| 1183 | Q8WW12 | TLSVAAAFNEDEDsEPEEMPPEAK |
| 1184 | Q8WVT3 | VRDEAEPGGEGDPGPEPAGtPsPSGEADGDCAPEDAAPSSGGAPR |
| 1185 | Q8WVT3 | VRDEAEPGGEGDPGPEPAGtPSPsGEADGDCAPEDAAPSSGGAPR |
| 1186 | Q8WVM8 | VNLEESSGVENsPAGARPK |
| 1187 | Q8WVM7 | MITSELPVLQDSTNETTAHSDAGsELEEtEVK |
| 1188 | Q8WVM7 | MITSELPVLQDSTNETTAHsDAGSELEEtEVK |
| 1189 | Q8WVC0 | ADMEDLFGsDADsEAER |
| 1190 | Q8WVC0 | ADMEDLFGsDADsEAER |
| 1191 | Q8WVC0 | KLtsDEEGEPSGK |
| 1192 | Q8WVC0 | KLTsDEEGEPSGK |
| 1193 | Q8WVC0 | KYVIsDEEEEDDD |
| 1194 | Q8WVC0 | KNAIAsDsEADsDTEVPK |
| 1195 | Q8WVC0 | KNAIAsDsEADsDTEVPK |
| 1196 | Q8WVC0 | KNAIAsDsEADsDTEVPK |
| 1197 | Q8WUX9 | IsDAELEAELEK |
| 1198 | Q8WUI4 | KEsAPPSLR |
| 1199 | Q8WUB8 | YLPLNTALYEPPLDPELPALDsDGDsDDGEDGRGDEK |
| 1200 | Q8WUB8 | YLPLNTALYEPPLDPELPALDsDGDsDDGEDGRGDEK |
| 1201 | Q8WUB8 | GTSDSSSGNVsEGESPPDSQEDSFQGR |
| 1202 | Q8WUA4 | DLDRPESQsPK |
| 1203 | Q8TF72 | DRPGsPEsPLLDAPFSR |
| 1204 | Q8TF72 | DRPGsPESPLLDAPFSR |
| 1205 | Q8TEP8 | GEVISsGSKPLsPGPCLDIPSILSNK |
| 1206 | Q8TEP8 | GEVISsGSKPLsPGPCLDIPSILSNK |
| 1207 | Q8TEA8 | SASsGAEGDVSSEREP |
| 1208 | Q8TEA8 | SAsSGAEGDVSSEREP |
| 1209 | Q8TE77 | QAsVHDSGEEGEA |
| 1210 | Q8TE77 | QAsVHDsGEEGEA |
| 1211 | Q8TE77 | sPPGSGASTPVGPWDQAVQR |
| 1212 | Q8TE67 | LGsTSHFPQEK |
| 1213 | Q8TE67 | SGYIPSNILEPLQPGtPGTQGQSPsR |
| 1214 | Q8TE67 | RSssPEDPERDEEVLNHVLR |
| 1215 | Q8TE67 | sSsPEDPERDEEVLNHVLR |
| 1216 | Q8TE67 | SGYIPSNILEPLQPGtPGTQGQsPSR |
| 1217 | Q8TDN4 | TLsGsPRPK |
| 1218 | Q8TDN4 | TLsGsPRPK |
| 1219 | Q8TDD1 | GAASQARGsDsEDGEFEIQAEDDAR |
| 1220 | Q8TDD1 | GAASQARGsDsEDGEFEIQAEDDAR |
| 1221 | Q8TDD1 | IDDRDsDEEGASDR |

| | | |
|---|---|---|
| 1222 | Q8TDB6 | DSCIsPSEPETK |
| 1223 | Q8TCJ2 | ENPPVEDsSDEDDKR |
| 1224 | Q8TCJ2 | ENPPVEDssDEDDKR |
| 1225 | Q8TC07 | NDsPTQIPVSSDVCR |
| 1226 | Q8TBF4 | KSTYFsDEEELsD |
| 1227 | Q8TBF4 | KSTYFsDEEELsD |
| 1228 | Q8TBB5 | QVLTAPGSAGQPRsEDEDSLEEAGSPAPGPCPR |
| 1229 | Q8TBB5 | QVLTAPGSAGQPRsEDEDsLEEAGSPAPGPCPR |
| 1230 | Q8TB72 | QAsPTEVVER |
| 1231 | Q8TAQ2 | KRsPSPsPtPEAK |
| 1232 | Q8TAQ2 | KRsPsPSPTPEAK |
| 1233 | Q8TAQ2 | TLTDEVNsPDSDR |
| 1234 | Q8TAD8 | LsPEVAPPAHR |
| 1235 | Q8TAD8 | RPDHSGGSPsPPTSEPAR |
| 1236 | Q8TAD8 | RPDHSGGsPSPPTSEPAR |
| 1237 | Q8NI35 | ALTDDsDENEEEDAFTDQK |
| 1238 | Q8NI27 | IDTHPsPSHSSTVK |
| 1239 | Q8NHZ8 | EDVEVVGGsDGEGAIGLSSDPK |
| 1240 | Q8NHQ9 | TVDLGIsDLEDDC |
| 1241 | Q8NHM5 | TENSLANENQQPIKsEPEsEGEEPK |
| 1242 | Q8NHM5 | TENSLANENQQPIKsEPEsEGEEPK |
| 1243 | Q8NHG8 | DRPVGGsPGGPR |
| 1244 | Q8NHG8 | sLGGAVGSVASGAR |
| 1245 | Q8NFC6 | DKDVTLsPVK |
| 1246 | Q8NFC6 | YYsDsDDELTVEQR |
| 1247 | Q8NFC6 | YYsDsDDELTVEQR |
| 1248 | Q8NFC6 | KQHYLssEDEPDDNPDVLDSR |
| 1249 | Q8NFC6 | KQHYLssEDEPDDNPDVLDSR |
| 1250 | Q8NEY8 | DNTFFREsPVGR |
| 1251 | Q8NEM2 | GLFQDEDsCSDCSYR |
| 1252 | Q8NEM2 | GLFQDEDSCsDCSYR |
| 1253 | Q8NEL9 | EPTSVSENEGISTIPsPVTSPVLSR |
| 1254 | Q8NE71 | KLsVPtsDEEDEVPAPKPR |
| 1255 | Q8NE71 | KLSVPTsDEEDEVPAPKPR |
| 1256 | Q8NE71 | KLSVPtsDEEDEVPAPKPR |
| 1257 | Q8NE71 | AVsEEQQPALK |
| 1258 | Q8NE71 | AEQGsEEEGEGEEEEEEGGESK |
| 1259 | Q8NDX5 | MDRtPPPPTLsPAAITVGR |
| 1260 | Q8NDX5 | MDRtPPPPTLsPAAITVGR |
| 1261 | Q8NDT2 | QRsLsPVAAPPLR |
| 1262 | Q8NDT2 | QRsLsPVAAPPLR |

| | | |
|---|---|---|
| 1263 | Q8NDT2 | ASGDPGASGMsPR |
| 1264 | Q8NDI1 | KPsEDEVLNK |
| 1265 | Q8NDI1 | ATDEDMQsLAsLMSMK |
| 1266 | Q8NDI1 | ATDEDMQsLAsLMSMK |
| 1267 | Q8NDI1 | DLSTSPKPsPIPsPVLGR |
| 1268 | Q8NDI1 | DLSTSPKPsPIPsPVLGR |
| 1269 | Q8NDI1 | DLSTsPKPSPIPsPVLGR |
| 1270 | Q8ND56 | sPVSTRPLPSASQK |
| 1271 | Q8ND30 | TLsINEEEPEGGFSK |
| 1272 | Q8NCF5 | TEFLDLDNSPLSPPsPR |
| 1273 | Q8NC44 | QALDsEEEEEDVAAK |
| 1274 | Q8NC44 | NAPPGGDEPLAEtEsESEAELAGFSPVVDVK |
| 1275 | Q8NC44 | NAPPGGDEPLAEtEsESEAELAGFSPVVDVK |
| 1276 | Q8NC44 | ATTPQLTDVSEDLDQQsLPsEPEETLSR |
| 1277 | Q8NC44 | ATTPQLTDVSEDLDQQsLPsEPEETLSR |
| 1278 | Q8NB78 | AAATGNAsPGKLEHSK |
| 1279 | Q8NAV1 | VSALEEDMDDVEssEEEEEEDEK |
| 1280 | Q8NAV1 | VSALEEDMDDVEssEEEEEEDEK |
| 1281 | Q8NA72 | TDESsPVLsPR |
| 1282 | Q8N9B5 | EASPEsEDEEEALPCTDWEN |
| 1283 | Q8N8A6 | RVNDAEPGsPEAPQGK |
| 1284 | Q8N5Y2 | SSsPIPLtPSK |
| 1285 | Q8N5P1 | IDDEIDtEVEETQEEK |
| 1286 | Q8N5F7 | NPEPDsDEHtPVEDEEPK |
| 1287 | Q8N5F7 | NPEPDsDEHtPVEDEEPK |
| 1288 | Q8N5F7 | IGELGAPEVWGLsPK |
| 1289 | Q8N556 | SGTSSPQsPVFR |
| 1290 | Q8N556 | KKPSTDEQTssAEEDVPTCGYLNVLSNSR |
| 1291 | Q8N556 | KKPSTDEQTssAEEDVPTCGYLNVLSNSR |
| 1292 | Q8N556 | SGTsSPQsPVFR |
| 1293 | Q8N488 | DKEIsPSVTK |
| 1294 | Q8N3X1 | ATGGLCLLGAYADsDDDDNDVSEK |
| 1295 | Q8N3X1 | ALEEGDGSVSGSsPR |
| 1296 | Q8N3V7 | DRAsPAAAEEVVPEWASCLK |
| 1297 | Q8N3V7 | SSPGLYTSPGQDSLQPTAVSPPYGGDIsPVSPsR |
| 1298 | Q8N3V7 | SSPGLYTSPGQDSLQPTAVSPPYGGDIsPVsPSR |
| 1299 | Q8N3V7 | SSPGLYTSPGQDSLQPTAVsPPYGGDISPVSPSR |
| 1300 | Q8N3V7 | SSPGLYTSPGQDSLQPTAVSPPyGGDISPVsPSR |
| 1301 | Q8N3U4 | NSLLAGGDDDTMSVIsGISSR |
| 1302 | Q8N3U4 | NSLLAGGDDDTMsVISGISSR |
| 1303 | Q8N3U4 | NSLLAGGDDDTMSVIsGIsSR |

| | | |
|---|---|---|
| 1304 | Q8N3F8 | APsAsPLALHASR |
| 1305 | Q8N3F8 | APsAsPLALHASR |
| 1306 | Q8N3E9 | ALsDREEEEEDDEEEEEEVEAAAQR |
| 1307 | Q8N3D4 | KGsDALRPPVPQGEDEVPK |
| 1308 | Q8N350 | LAEPVVATPALVAAAPTsPDHsPA |
| 1309 | Q8N350 | LAEPVVATPALVAAAPTsPDHsPA |
| 1310 | Q8N302 | ASEAPsPPRsPPPPTSPEPELAQLR |
| 1311 | Q8N302 | ASEAPSPPRsPPPPtSPEPELAQLR |
| 1312 | Q8N1P7 | EPsPHPGVGLTSGSSR |
| 1313 | Q8N1P7 | GVQDSEGsPISSLTQK |
| 1314 | Q8N1P7 | MVPPVVVGsPPGsPSR |
| 1315 | Q8N1P7 | MVPPVVVGsPPGsPSR |
| 1316 | Q8N1P7 | DVPSPGGLSAPSsPR |
| 1317 | Q8N1G0 | HGLQLGAQsPGR |
| 1318 | Q8N163 | LEDSEVRSVASNQsEMEFSSLQDMPK |
| 1319 | Q8N163 | LEDSEVRsVAsNQsEMEFSSLQDMPK |
| 1320 | Q8N163 | LEDSEVRsVAsNQsEMEFSSLQDMPK |
| 1321 | Q8N128 | RVIHFVSGETMEEYstDEDEVDGLEK |
| 1322 | Q8N128 | RVIHFVSGETMEEYstDEDEVDGLEK |
| 1323 | Q8N122 | VLDTSSLTQSAPAsPTNK |
| 1324 | Q8N122 | VLDTSSLTQsAPAsPTNK |
| 1325 | Q8N108 | YFDTNsEVEEEsEEDEDYIPSEDWK |
| 1326 | Q8N108 | YFDTNsEVEEEsEEDEDYIPSEDWK |
| 1327 | Q8N108 | LLDESEsAAsSR |
| 1328 | Q8N108 | LLDESEsAAsSR |
| 1329 | Q8IZL8 | GsPDGSLQTGKPSAPK |
| 1330 | Q8IZL8 | LRsPRGSPDGsLQTGKPSAPK |
| 1331 | Q8IZ21 | SSsPVQVEEEPVR |
| 1332 | Q8IYH5 | GLsSSEKDNIER |
| 1333 | Q8IYB3 | AsPsPPPKR |
| 1334 | Q8IYB3 | AsPsPPPKR |
| 1335 | Q8IYB3 | REsPSPAPKPR |
| 1336 | Q8IYB3 | VPKPEPIPEPKEPsPEK |
| 1337 | Q8IYB3 | REsPsPAPKPR |
| 1338 | Q8IYB3 | RRtAsPPPPPK |
| 1339 | Q8IYB3 | RRtPtPPPR |
| 1340 | Q8IYB3 | RRtPtPPPR |
| 1341 | Q8IYB3 | RRsPsPAPPPR |
| 1342 | Q8IYB3 | RRsPsPAPPPR |
| 1343 | Q8IYB3 | RRtPsPPPR |
| 1344 | Q8IYB3 | RRtPsPPPR |

| | | |
|---|---|---|
| 1345 | Q8IYB3 | RRtAsPPPPPK |
| 1346 | Q8IYB3 | KSRVsVsPGR |
| 1347 | Q8IYB3 | KSRVsVsPGR |
| 1348 | Q8IYB3 | KVELsESEEDK |
| 1349 | Q8IYB3 | HRPsPPAtPPPK |
| 1350 | Q8IYB3 | HRPsPPAtPPPK |
| 1351 | Q8IYB3 | AAsPSPQsVR |
| 1352 | Q8IYB3 | KVELSEsEEDKGGK |
| 1353 | Q8IYB3 | AAsPSPQSVR |
| 1354 | Q8IYB3 | AAsPsPQSVR |
| 1355 | Q8IYB3 | KETEsEAEDNLDDLEK |
| 1356 | Q8IYB3 | KEtEsEAEDNLDDLEK |
| 1357 | Q8IYB3 | LsPSAsPPR |
| 1358 | Q8IYB3 | LsPSAsPPR |
| 1359 | Q8IYB3 | RLSPsAsPPR |
| 1360 | Q8IYB3 | RRsPsPPPTR |
| 1361 | Q8IYB3 | RRsPsPPPTR |
| 1362 | Q8IYB3 | QsPsPSTRPIR |
| 1363 | Q8IYB3 | QsPsPSTRPIR |
| 1364 | Q8IYB3 | EKtPELPEPSVK |
| 1365 | Q8IYB3 | RYsPPIQR |
| 1366 | Q8IYB3 | RYsPsPPPK |
| 1367 | Q8IYB3 | RYsPsPPPK |
| 1368 | Q8IYB3 | RQSPsPStRPIR |
| 1369 | Q8IYB3 | SVsGsPEPAAK |
| 1370 | Q8IYB3 | sVSGsPEPAAK |
| 1371 | Q8IYB3 | APQTSSsPPPVR |
| 1372 | Q8IYB3 | KPPAPPsPVQSQsPSTNWSPAVPVK |
| 1373 | Q8IYB3 | KPPAPPsPVQSQsPSTNWSPAVPVK |
| 1374 | Q8IYB3 | APQTsSsPPPVR |
| 1375 | Q8IY81 | ALDISLssGEEDEGDEEDSTAGTTK |
| 1376 | Q8IY81 | ALDISLssGEEDEGDEEDSTAGTTK |
| 1377 | Q8IY81 | ALDIsLSsGEEDEGDEEDSTAGTTK |
| 1378 | Q8IY67 | AADVSVTHRPPLsPK |
| 1379 | Q8IY17 | IEPPTSYVSDGCADGEEsDCLtEYEEDAGPDCSR |
| 1380 | Q8IY17 | IEPPTSYVSDGCADGEEsDCLtEYEEDAGPDCSR |
| 1381 | Q8IXT5 | RPLEEDFRRsPtEDFR |
| 1382 | Q8IXT5 | sRsPLGFYVHLK |
| 1383 | Q8IXT5 | sRsPLGFYVHLK |
| 1384 | Q8IXM2 | KVYEDSGIPLPAEsPK |
| 1385 | Q8IX90 | sPQLSDFGLER |

| | | |
|---|---|---|
| 1386 | Q8IX03 | LGASEAAAFDsDESEAVGATR |
| 1387 | Q8IWX8 | sPtPPSSAGLGSNSAPPIPDSR |
| 1388 | Q8IWX8 | sRsPtPPSSAGLGSNSAPPIPDSR |
| 1389 | Q8IWX8 | sPtPPSSAGLGSNSAPPIPDSR |
| 1390 | Q8IWW6 | ATtPPNQGRPDsPVYANLQELK |
| 1391 | Q8IWW6 | ATtPPNQGRPDsPVYANLQELK |
| 1392 | Q8IWW6 | AtTPPNQGRPDsPVYANLQELK |
| 1393 | Q8IWE2 | IHGVNSGSSEGAQPNTENGVPEITDAATDQGPAEsPPTSPSSASR |
| 1394 | Q8IWE2 | IHGVNSGSSEGAQPNTENGVPEITDAATDQGPAESPPTsPSSASR |
| 1395 | Q8IW50 | GYSSLDQSPDEKPLVALDtDSDDDFDMsR |
| 1396 | Q8IW50 | GYSSLDQSPDEKPLVALDtDsDDDFDMSR |
| 1397 | Q8IVT2 | LQKsQSSDLLER |
| 1398 | Q8IVT2 | NALFPEVFsPTPDENSDQNSR |
| 1399 | Q8IVT2 | ALSsDSILSPAPDAR |
| 1400 | Q8IVT2 | ALsSDSILSPAPDAR |
| 1401 | Q8IVT2 | HLsESSGKPLSTK |
| 1402 | Q8IVT2 | AStPDWVSEGPQPGLR |
| 1403 | Q8IVT2 | SQsSDLLER |
| 1404 | Q8IVT2 | ALSSDSILsPAPDAR |
| 1405 | Q8IVL1 | TTLSEsPLSsPAAsPK |
| 1406 | Q8IVL1 | TTLSEsPLSsPAAsPK |
| 1407 | Q8IVL1 | TTLSEsPLSsPAAsPK |
| 1408 | Q8IVF2 | DAHDVsPTSTDTEAQLTVER |
| 1409 | Q8IVF2 | DAHDVsPTStDTEAQLTVER |
| 1410 | Q8IUW5 | AMVADNSLYDPESPVTPSTPGSPPVsPGPLSPGGTPGK |
| 1411 | Q8IUW5 | AMVADNSLYDPESPVTPSTPGsPPVsPGPLSPGGTPGK |
| 1412 | Q8IU81 | KAsPEPEGEAAGK |
| 1413 | Q8IU81 | AGGAsPAASSTAQPPTQHR |
| 1414 | Q86YS7 | NQTYSFsPSK |
| 1415 | Q86YP4 | RPPsPDVIVLsDNEQPSSPR |
| 1416 | Q86YP4 | RPPsPDVIVLSDNEQPSsPR |
| 1417 | Q86YP4 | RPPsPDVIVLSDNEQPSsPR |
| 1418 | Q86YP4 | RPPsPDVIVLSDNEQPsSPR |
| 1419 | Q86XP3 | YMAENPTAGVVQEEEEDNLEYDsDGNPIAPTK |
| 1420 | Q86X95 | NLTANDPSQEYVAsEGEEDPEVEFLK |
| 1421 | Q86X53 | EEDGADAsEEDLTR |
| 1422 | Q86X27 | KSVAAEGALLPQtPPsPR |
| 1423 | Q86X27 | KSVAAEGALLPQtPPsPR |
| 1424 | Q86X27 | SSsSEsLSDKGSELKK |
| 1425 | Q86X27 | SSsSEsLSDKGSELKK |
| 1426 | Q86WR7 | APsPPVEHPR |

| | | |
|---|---|---|
| 1427 | Q86WR7 | MAGNEALsPTSPFR |
| 1428 | Q86WR7 | MAGNEALsPTsPFR |
| 1429 | Q86WR7 | SRsFTLDDESLK |
| 1430 | Q86WR7 | sRsFTLDDESLK |
| 1431 | Q86WB0 | DTSATSQSVNGsPQAEQPSLESTSK |
| 1432 | Q86WB0 | SQDATFsPGSEQAEK |
| 1433 | Q86WB0 | SQDATFsPGsEQAEK |
| 1434 | Q86WB0 | SQDATFSPGSEQAEKsPGPIVSR |
| 1435 | Q86WB0 | sWDSSSPVDRPEPEAASPTTR |
| 1436 | Q86WB0 | SWDSSSPVDRPEPEAAsPTTR |
| 1437 | Q86WB0 | SWDSSsPVDRPEPEAAsPTTR |
| 1438 | Q86W92 | SSsLGNLK |
| 1439 | Q86W92 | ALEYSNGIFDCQsPTSPFMGSLR |
| 1440 | Q86W92 | ALEYSNGIFDCQSPtsPFMGSLR |
| 1441 | Q86W92 | DFAARsPSAsITDEDSNV |
| 1442 | Q86W92 | DFAARsPSAsITDEDSNV |
| 1443 | Q86W56 | SCDPGEDCASCQQDEIDVVPEsPLsDVGSEDVGTGPK |
| 1444 | Q86W56 | SCDPGEDCASCQQDEIDVVPEsPLsDVGSEDVGTGPK |
| 1445 | Q86W56 | LENVSQLSLDKsPTEK |
| 1446 | Q86VR2 | AMDNHsDsEEELAAFCPQLDDSTVAR |
| 1447 | Q86VR2 | AMDNHsDsEEELAAFCPQLDDSTVAR |
| 1448 | Q86VR2 | GQTPLTEGsEDLDGHSDPEEsFAR |
| 1449 | Q86VR2 | GQTPLTEGsEDLDGHsDPEESFAR |
| 1450 | Q86VR2 | ELAItDsEHsDAEVSCTDNGTFNLSR |
| 1451 | Q86VR2 | ELAItDsEHsDAEVSCTDNGTFNLSR |
| 1452 | Q86VR2 | ELAItDsEHsDAEVSCTDNGTFNLSR |
| 1453 | Q86VM9 | LGsPKPER |
| 1454 | Q86VM9 | sPQPPSR |
| 1455 | Q86VM9 | KLGVsVsPSR |
| 1456 | Q86VM9 | KLGVsVsPSR |
| 1457 | Q86VM9 | SQDQDsEVNELSR |
| 1458 | Q86VM9 | AsDLEDEESAAR |
| 1459 | Q86V48 | EKPDsDDDLDIASLVTAK |
| 1460 | Q86V48 | SHsAPSEVGFSDAR |
| 1461 | Q86UU1 | TLQPPEsPR |
| 1462 | Q86UU1 | KLsSGDLR |
| 1463 | Q86UU1 | TRSPsPtLGESLAPHK |
| 1464 | Q86UU1 | TRsPsPTLGESLAPHK |
| 1465 | Q86UU1 | tRSPSPTLGESLAPHK |
| 1466 | Q86UU1 | TFsDGLATR |
| 1467 | Q86UU1 | KNsITEIsDNEDDLLEYHR |

| | | |
|---|---|---|
| 1468 | Q86UU1 | KNsITEISDNEDDLLEYHR |
| 1469 | Q86UU0 | EAPGsPPLsPR |
| 1470 | Q86UU0 | EAPGsPPLSPR |
| 1471 | Q86UU0 | DRsVsVDSGEQR |
| 1472 | Q86UU0 | ANQIsPSNSSLK |
| 1473 | Q86US8 | TQTPQLHFLDtDDEVsPTSWGDSR |
| 1474 | Q86US8 | TQTPQLHFLDTDDEVsPTSWGDSR |
| 1475 | Q86UP2 | EIQNGNLHEsDSESVPR |
| 1476 | Q86UE8 | SVSTSSPAGAAIASTSGASNNsSSN |
| 1477 | Q86U86 | LSAITMALQYGsEsEEDAALAAAR |
| 1478 | Q86U86 | LSAITMALQYGsEsEEDAALAAAR |
| 1479 | Q86TN4 | KPLSLAGDEETECQSsPK |
| 1480 | Q86TC9 | TPVDEsDDEIQHDEIPTGK |
| 1481 | Q86TC9 | EAEQAASEAAGGDTtPGSsPSSLYYEEPLGQPPR |
| 1482 | Q86TC9 | EAEQAASEAAGGDTtPGSsPSSLYYEEPLGQPPR |
| 1483 | Q86TB9 | RSTsPIIGsPPVR |
| 1484 | Q86TB9 | RsTSPIIGsPPVR |
| 1485 | Q86TB9 | RSTsPIIGsPPVR |
| 1486 | Q86T82 | SSLALCLDsDsEDELKR |
| 1487 | Q86T82 | SSLALCLDsDsEDELKR |
| 1488 | Q86SQ0 | NFsCGsVEFDEADLESLR |
| 1489 | Q86SQ0 | NFsCGsVEFDEADLESLR |
| 1490 | Q86SQ0 | LsTGTTVEDVQK |
| 1491 | Q86SQ0 | TSASEGNPYVSSTLSVPAsPR |
| 1492 | Q7Z7F0 | GFGLVAYAADssDEEEEHGGHK |
| 1493 | Q7Z7F0 | GFGLVAYAADssDEEEEHGGHK |
| 1494 | Q7Z6Z7 | AEsPEEVACR |
| 1495 | Q7Z6Z7 | DGGsGNSTIIVSR |
| 1496 | Q7Z6Z7 | DGGSGNsTIIVSR |
| 1497 | Q7Z6Z7 | GSGTAsDDEFENLR |
| 1498 | Q7Z6Z7 | DEPPPLsPAPLtPATPSSLDPFFSR |
| 1499 | Q7Z6Z7 | DEPPPLsPAPLtPATPSSLDPFFSR |
| 1500 | Q7Z6Z7 | DEPPPLsPAPLtPAtPSSLDPFFSR |
| 1501 | Q7Z6Z7 | AQCETLsPDGLPEEQPQTTK |
| 1502 | Q7Z6Z7 | SNHAAEEAssEDEEEEEVQAMQSFNSTQQNETEPNQQVVGTEER |
| 1503 | Q7Z6Z7 | SNHAAEEAssEDEEEEEVQAMQSFNSTQQNETEPNQQVVGTEER |
| 1504 | Q7Z6Z7 | sHHAASTTTAPTPAAR |
| 1505 | Q7Z6Z7 | sNHAAEEASSEDEEEEEVQAMQSFNSTQQNETEPNQQVVGTEER |
| 1506 | Q7Z6E9 | ENFsPER |
| 1507 | Q7Z6E9 | LEVTEIVKPsPK |
| 1508 | Q7Z5L9 | KPsPEPEGEVGPPK |

| | | |
|---|---|---|
| 1509 | Q7Z5L9 | LEEPPELNRQsPNPR |
| 1510 | Q7Z5L9 | NsNSPPsPSSMNQR |
| 1511 | Q7Z5L9 | NSNsPPsPSSMNQR |
| 1512 | Q7Z5L9 | NSNSPPsPSSMNQR |
| 1513 | Q7Z5L9 | RPAsVSSSAAVEHEQR |
| 1514 | Q7Z5K2 | VEEESTGDPFGFDsDDESLPVSSK |
| 1515 | Q7Z5K2 | RPEsPSEIsPIK |
| 1516 | Q7Z5K2 | RPEsPSEISPIK |
| 1517 | Q7Z5K2 | YFGFDDLsEsEDDEDDDCQVER |
| 1518 | Q7Z5K2 | YFGFDDLsEsEDDEDDDCQVER |
| 1519 | Q7Z5J4 | GAGGsPVGVEEGLVNVGTGQK |
| 1520 | Q7Z589 | RRtNsSSSSPVVLK |
| 1521 | Q7Z589 | RRtNsSSSSPVVLK |
| 1522 | Q7Z569 | ECINAAPDsPSK |
| 1523 | Q7Z4V5 | ARGDsEALDEES |
| 1524 | Q7Z4V5 | GESAEDKEHEEGRDsEEGPR |
| 1525 | Q7Z4S6 | EKELsPPPGLPSK |
| 1526 | Q7Z4S6 | NLQDGQISDTGDLGEDIAsN |
| 1527 | Q7Z4S6 | LTVSQGNTSVQQDKsDEsDSSLSEVHR |
| 1528 | Q7Z4S6 | LTVSQGNTSVQQDKsDEsDSSLSEVHR |
| 1529 | Q7Z4S6 | IPEPsPVTR |
| 1530 | Q7Z4S6 | KLsSSDAPAQDTGSSAAAVETDASR |
| 1531 | Q7Z4S6 | NLQDGQIsDTGDLGEDIASN |
| 1532 | Q7Z460 | NSSNTSVGsPSNTIGR |
| 1533 | Q7Z460 | SRsDIDVNAAASAK |
| 1534 | Q7Z422 | sKsPPKVPIVIQDDSLPAGPPPQIR |
| 1535 | Q7Z422 | sKsPPKVPIVIQDDSLPAGPPPQIR |
| 1536 | Q7Z417 | NDsWGSFDLR |
| 1537 | Q7Z417 | NLsSDEATNPISR |
| 1538 | Q7Z417 | GADNDGsGSESGYTTPK |
| 1539 | Q7Z417 | GADNDGsGsESGYTTPK |
| 1540 | Q7Z406 | LEEGVAsDEEAEEAQPGSGPSPEPEGSPPAHPQ |
| 1541 | Q7Z406 | LEEGVAsDEEAEEAQPGSGPSPEPEGsPPAHPQ |
| 1542 | Q7Z406 | LEEGVAsDEEAEEAQPGSGPsPEPEGSPPAHPQ |
| 1543 | Q7Z333 | GQVIIIsDsDDDDDER |
| 1544 | Q7Z333 | GQVIIIsDsDDDDDER |
| 1545 | Q7Z309 | IDFTPVsPAPsPTR |
| 1546 | Q7Z309 | IDFTPVsPAPsPTR |
| 1547 | Q7Z2W4 | FFQGSQEFLASASASAERsCtPsPDQISHR |
| 1548 | Q7Z2W4 | FFQGSQEFLASASASAERsCtPsPDQISHR |
| 1549 | Q7Z2W4 | AsLEDAPVDDLTR |

| | | |
|---|---|---|
| 1550 | Q7Z2W4 | FFQGSQEFLASASASAERsCtPsPDQISHR |
| 1551 | Q7Z2W4 | TVFsPTLPAAR |
| 1552 | Q7Z2W4 | FFQGSQEFLASASAsAERsCTPsPDQISHR |
| 1553 | Q7RTP6 | GPsQATsPIR |
| 1554 | Q7RTP6 | GPsQATsPIR |
| 1555 | Q7L9L4 | HAEAtLGSGNLR |
| 1556 | Q7L8J4 | GLSDHVsLDGQELGTR |
| 1557 | Q7L4I2 | EQSEVSVsPR |
| 1558 | Q7L4I2 | AAsDTERDGLAPEKTsPDR |
| 1559 | Q7L4I2 | AAsDTERDGLAPEK |
| 1560 | Q7L2J0 | TLNAEtPKSsPLPAK |
| 1561 | Q7L2J0 | CAPSAGsPAAAVGREsPGAAATSSSGPQAQQHR |
| 1562 | Q7L2J0 | CAPSAGsPAAAVGREsPGAAATSSSGPQAQQHR |
| 1563 | Q7L2J0 | TLNAEtPKsSPLPAK |
| 1564 | Q7L1V2 | DKDQPPsPSPPPQSEALSSTSR |
| 1565 | Q7L1V2 | DKDQPPSPsPPPQSEALSSTSR |
| 1566 | Q7L1Q6 | FVEWLKNAEEEsEsEAEEGD |
| 1567 | Q7L1Q6 | FVEWLKNAEEEsEsEAEEGD |
| 1568 | Q7L014 | AALGLQDsDDEDAAVDIDEQIESMFNSK |
| 1569 | Q7KZ85 | SDFVEsEAEEsEEEYNDEGEVVPR |
| 1570 | Q7KZ85 | SDFVEsEAEEsEEEYNDEGEVVPR |
| 1571 | Q76FK4 | FLEtDsEEEQEEVNEK |
| 1572 | Q76FK4 | FLEtDsEEEQEEVNEK |
| 1573 | Q76FK4 | NSsPGEASLLEK |
| 1574 | Q71RC2 | DGLNQTTIPVsPPSTTKPSR |
| 1575 | Q70J99 | DVSGFSDPYCLLGIEQGVGVPGGsPGSR |
| 1576 | Q70E73 | MEQLsDEEIDHGAEEDsDKEDQDLDK |
| 1577 | Q70E73 | MEQLsDEEIDHGAEEDsDKEDQDLDK |
| 1578 | Q6ZW76 | DVTsPINER |
| 1579 | Q6ZUT6 | sPPTQVAISSDSAR |
| 1580 | Q6ZUT6 | GESVASTASsVPCsPQEPDLAPLDLSLGGAGIPGPR |
| 1581 | Q6ZUT6 | GESVASTASsVPCsPQEPDLAPLDLSLGGAGIPGPR |
| 1582 | Q6ZTN6 | GPGsPPRtPPAPGPPSFEEQLR |
| 1583 | Q6ZTN6 | GPGsPPRtPPAPGPPSFEEQLR |
| 1584 | Q6ZSZ5 | SLsPILPGR |
| 1585 | Q6ZSZ5 | sLsPILPGR |
| 1586 | Q6ZSR9 | LGGAVPFAPPEVsPEQAK |
| 1587 | Q6ZS17 | FSTYSQsPPDtPSLR |
| 1588 | Q6ZS17 | FSTYSQsPPDTPSLR |
| 1589 | Q6ZRV2 | RLsLGQGDSTEAATEER |
| 1590 | Q6ZRS2 | LQPPSPLGPEGSVEEsEAEAsGEEEEGDGTPR |

| | | |
|---|---|---|
| 1591 | Q6ZRS2 | LQPPSPLGPEGSVEEsEAEAsGEEEEGDGTPR |
| 1592 | Q6ZRS2 | LQPPsPLGPEGSVEEsEAEAsGEEEEGDGTPR |
| 1593 | Q6ZMQ8 | CYSVQEPsEDsEEEAPAVPVVVAESQSAR |
| 1594 | Q6ZMQ8 | CYSVQEPsEDsEEEAPAVPVVVAESQSAR |
| 1595 | Q6Y7W6 | SQsWEER |
| 1596 | Q6Y7W6 | ALSSGGSITsPPLsPALPK |
| 1597 | Q6Y7W6 | ALSSGGSITsPPLsPALPK |
| 1598 | Q6Y7W6 | SEsENWR |
| 1599 | Q6Y7W6 | ALsSGGSITsPPLSPALPK |
| 1600 | Q6XQN6 | LQALVNSLCAGQsP |
| 1601 | Q6WKZ4 | DSGSDTAsAIIPSTTPSVDSDDESVVKDK |
| 1602 | Q6WKZ4 | DSGSDTASAIIPSTTPSVDSDDEsVVK |
| 1603 | Q6WCQ1 | DQPDGSSLsPAQSPSQSQPPAASSLR |
| 1604 | Q6WCQ1 | DQPDGSSLsPAQsPSQSQPPAASSLR |
| 1605 | Q6WCQ1 | SKsNPDFLK |
| 1606 | Q6VN20 | SQDSYPGsPSLsPR |
| 1607 | Q6VN20 | SQDSYPGsPSLsPR |
| 1608 | Q6UN15 | DHsPTPSVFNSDEER |
| 1609 | Q6UN15 | DHSPtPSVFNSDEER |
| 1610 | Q6UN15 | DHsPTPsVFNSDEER |
| 1611 | Q6T4R5 | VQQEIDsDEsPVAR |
| 1612 | Q6T4R5 | VQQEIDsDESPVAR |
| 1613 | Q6R327 | GRNDsGEENVPLDLTR |
| 1614 | Q6QNY0 | VAGEAAEtDsEPEPEPEPTAAPR |
| 1615 | Q6QNY0 | VAGEAAEtDsEPEPEPEPTAAPR |
| 1616 | Q6Q0C0 | RSDsAIsVR |
| 1617 | Q6Q0C0 | TPsSSsTLAYSPR |
| 1618 | Q6Q0C0 | TPsSSsTLAYSPR |
| 1619 | Q6PKG0 | TPRtPRtPQLK |
| 1620 | Q6PKG0 | TPRtPRtPQLK |
| 1621 | Q6PKG0 | tPRTPRtPQLK |
| 1622 | Q6PKG0 | ETESAPGsPR |
| 1623 | Q6PKG0 | ETEsAPGsPR |
| 1624 | Q6PKG0 | sLPTTVPESPNYR |
| 1625 | Q6PKG0 | SLPTTVPEsPNYR |
| 1626 | Q6PKG0 | KNTFTAWsDEEsDYEIDDR |
| 1627 | Q6PKG0 | KNTFTAWSDEEsDyEIDDR |
| 1628 | Q6PKG0 | GLSAsLPDLDSENWIEVK |
| 1629 | Q6PKG0 | EGTGQQEREsPRPLQLPGAEGPAIsDGEEGGGEPGAGGGAAGAAGAGR |
| 1630 | Q6PK04 | VQAGPGsPR |
| 1631 | Q6PJG2 | AsQEANLLTLAQK |

| | | |
|---|---|---|
| 1632 | Q6PJG2 | SFELPPYtPPPILsPVR |
| 1633 | Q6PJG2 | SFELPPYtPPPILsPVR |
| 1634 | Q6PJG2 | TNSAEVtPPVLSVMGEATPVSIEPR |
| 1635 | Q6PJG2 | TNSAEVtPPVLSVMGEAtPVSIEPR |
| 1636 | Q6PJG2 | tNSAEVtPPVLSVMGEAtPVSIEPR |
| 1637 | Q6PID6 | KSEAPAEVTHFsPK |
| 1638 | Q6PD74 | AFWMAIGGDRDEIEGLssDEEH |
| 1639 | Q6PD74 | AFWMAIGGDRDEIEGLssDEEH |
| 1640 | Q6PD62 | GsGsEQEGEDEEGGER |
| 1641 | Q6PD62 | GsGsEQEGEDEEGGER |
| 1642 | Q6PD62 | GGEFDEFVNDDtDDDLPISK |
| 1643 | Q6PD62 | GEEGsDDDETENGPKPK |
| 1644 | Q6PCB5 | AEPPsPVHCVAAAAPTATVSEKEPFGK |
| 1645 | Q6P6C2 | YQEDSDPERsDyEEQQLQKEEEAR |
| 1646 | Q6P6C2 | YQEDsDPERSDYEEQQLQK |
| 1647 | Q6P4R8 | SEAEDLAEPLSSTEGVAPLSQAPsPLAIPAIK |
| 1648 | Q6P4R8 | SEAEDLAEPLSSTEGVAPLsQAPsPLAIPAIK |
| 1649 | Q6P4R8 | SEAEDLAEPLsSTEGVAPLsQAPsPLAIPAIK |
| 1650 | Q6P2E9 | DSQDASAEQsDHDDEVASLASASGGFGTK |
| 1651 | Q6P1M3 | ARNsGTQsDGEEKQPGLVMER |
| 1652 | Q6P158 | DLQEQDADAGsERGLsGEEEDDEPDCCNDER |
| 1653 | Q6P158 | DLQEQDADAGsER |
| 1654 | Q6P0N0 | NTSNIPVILEPEtEEsENEFYIK |
| 1655 | Q6P0N0 | NTSNIPVILEPEtEEsENEFYIK |
| 1656 | Q6NZY4 | KSEAGHASsPDSEVTSLCQK |
| 1657 | Q6NYC8 | LsPGESAYQK |
| 1658 | Q6NWY9 | GsPSSHLLGADHGLR |
| 1659 | Q6KC79 | AITSLLGGGsPK |
| 1660 | Q6KC79 | NAASFPLRsPQPVCSPAGSEGtPK |
| 1661 | Q6KC79 | NAASFPLRsPQPVCsPAGSEGTPK |
| 1662 | Q6KB66 | sLTGCWSAGTISK |
| 1663 | Q6IQ23 | KVTsPLQsPTK |
| 1664 | Q6IQ23 | KVTsPLQsPTK |
| 1665 | Q6IN85 | TNLTSQSSTTNLPGSPGSPGsPGSPGsPGSVPK |
| 1666 | Q6IN85 | TNLTSQSSTTNLPGSPGSPGsPGSPGsPGSVPK |
| 1667 | Q6IE81 | DPLQNsPGSEGK |
| 1668 | Q6ICG6 | VTSFsTPPtPER |
| 1669 | Q6ICG6 | VTSFsTPPtPER |
| 1670 | Q6IBW4 | sPQQSAALPR |
| 1671 | Q6GYQ0 | RGsSPGsLEIPK |
| 1672 | Q6GYQ0 | RGssPGSLEIPK |

| | | |
|---|---|---|
| 1673 | Q6GQQ9 | STPEsGDsDKESVGSSSTSNEGGR |
| 1674 | Q6GQQ9 | STPESGDsDKESVGSSSTSNEGGR |
| 1675 | Q6DN90 | NsWDSPAFSNDVIR |
| 1676 | Q6BDS2 | TVsQQsFDGVSLDSSGPEDR |
| 1677 | Q6BDS2 | TVsQQsFDGVSLDSSGPEDR |
| 1678 | Q68EM7 | sPsPPTQHTGQPPGQPSAPSQLSAPR |
| 1679 | Q68EM7 | sPsPPTQHTGQPPGQPSAPSQLSAPR |
| 1680 | Q68DQ2 | SDGSDTTEQESTNLPsPNK |
| 1681 | Q68CZ2 | KLsLGQYDNDAGGQLPFSK |
| 1682 | Q676U5 | sVSSFPVPQDNVDTHPGSGK |
| 1683 | Q66PJ3 | sAGEEEDGPVLTDEQK |
| 1684 | Q66K74 | SAsPHDVDLCLVsPCEFEHR |
| 1685 | Q66K74 | sASPHDVDLCLVsPCEFEHR |
| 1686 | Q66K74 | LsLsPLR |
| 1687 | Q66K74 | LSLsPLR |
| 1688 | Q66K74 | SAsPHDVDLCLVsPCEFEHR |
| 1689 | Q66K14 | ASVVDPSTESSPAPQEGSEQPAsPAsPLSSR |
| 1690 | Q66K14 | ASVVDPSTESSPAPQEGSEQPASPAsPLSSR |
| 1691 | Q66K14 | KAsVVDPSTESSPAPQEGSEQPASPAsPLSSR |
| 1692 | Q659C4 | IGsPLsPK |
| 1693 | Q659C4 | IGsPLsPK |
| 1694 | Q658Y4 | KLsDASDER |
| 1695 | Q641Q2 | AGNsDsEEDDANGRVELILEPK |
| 1696 | Q641Q2 | AGNsDsEEDDANGRVELILEPK |
| 1697 | Q641Q2 | ERRtPsDDEEDNLFAPPK |
| 1698 | Q641Q2 | ERRtPsDDEEDNLFAPPK |
| 1699 | Q641Q2 | ASALLFssDEEDQWNIPASQTHLASDSR |
| 1700 | Q641Q2 | ASALLFssDEEDQWNIPASQTHLASDSR |
| 1701 | Q641Q2 | GLFsDEEDSEDLFSSQSASK |
| 1702 | Q641Q2 | LTDEDFsPFGsGGGLFSGGK |
| 1703 | Q641Q2 | LTDEDFsPFGSGGGLFSGGK |
| 1704 | Q641Q2 | RsRPTsFADELAAR |
| 1705 | Q641Q2 | RsRPTsFADELAAR |
| 1706 | Q641Q2 | VSLLFEDDVDSGGSLFGsPPTSVPPATK |
| 1707 | Q63HN8 | DFATPSLHTSDQsPGK |
| 1708 | Q5VZL5 | AKSEDsDVELsD |
| 1709 | Q5VZL5 | AKSEDsDVELsD |
| 1710 | Q5VZL5 | DNLVSSIHtDDsLEVER |
| 1711 | Q5VZL5 | DNLVSSIHtDDsLEVER |
| 1712 | Q5VZK9 | RsWGQQAQEYQEQK |
| 1713 | Q5VZK9 | DGQSsPQPSPR |

| | | |
|---|---|---|
| 1714 | Q5VZ89 | SCsFSSESR |
| 1715 | Q5VZ89 | THsFENVSCHLPDSR |
| 1716 | Q5VZ89 | HSQPsPEPHsPTEPPAWGSSIVK |
| 1717 | Q5VZ89 | HSQPsPEPHsPTEPPAWGSSIVK |
| 1718 | Q5VWG9 | IPPMLsPVHVQDSTDLAPPsPEPPMLAPVAK |
| 1719 | Q5VWG9 | IPPMLsPVHVQDSTDLAPPsPEPPMLAPVAK |
| 1720 | Q5VV41 | GLGKPGGQGDAIQLsPK |
| 1721 | Q5VV41 | HQsFGAAVLSR |
| 1722 | Q5VV41 | GHKGsFKDDPQLYQEIQER |
| 1723 | Q5VV41 | AQRHsDSSLEEK |
| 1724 | Q5VV41 | GLNTSQEsDDDILDESSSPEGTQK |
| 1725 | Q5VV41 | GLNTsQEsDDDILDESSSPEGTQK |
| 1726 | Q5VV41 | GLNtsQESDDDILDESSSPEGTQK |
| 1727 | Q5VUA4 | RVsPsPPR |
| 1728 | Q5VUA4 | RVsPsPPR |
| 1729 | Q5VUA4 | sPGLCSDSLEK |
| 1730 | Q5VTR2 | ALVVPEPEPDSDsNQER |
| 1731 | Q5VTR2 | ALVVPEPEPDsDSNQER |
| 1732 | Q5VTL8 | RRsQsIEQESQEK |
| 1733 | Q5VTL8 | RRsQsIEQESQEK |
| 1734 | Q5VT52 | DVEDMELsDVEDDGSK |
| 1735 | Q5VSL9 | AAsPPASASDLIEQQQK |
| 1736 | Q5UIP0 | VEEPSQCLASGTAISELIIEDNNAsPQK |
| 1737 | Q5UIP0 | SNEsVDIQDQEEK |
| 1738 | Q5UIP0 | ASQGLLSSIENsESDSSEAKEEGSR |
| 1739 | Q5UIP0 | ASQGLLSSIENsEsDSSEAK |
| 1740 | Q5THJ4 | EVQDKDYPLtPPPsPTVDEPK |
| 1741 | Q5THJ4 | EVQDKDYPLtPPPsPTVDEPK |
| 1742 | Q5TAQ9 | VHDRsEEEEEEEEEEEEEQPR |
| 1743 | Q5T8P6 | LNHsPPQSSSR |
| 1744 | Q5T8P6 | EGSTQQLQTTsPK |
| 1745 | Q5T8I3 | ILQSQDFSLDssAEEEGLR |
| 1746 | Q5T8I3 | ILQSQDFSLDssAEEEGLR |
| 1747 | Q5T8D3 | AESSDsGAEsEEEEAQEEVK |
| 1748 | Q5T8D3 | AESSDsGAEsEEEEAQEEVK |
| 1749 | Q5T4S7 | HVtLPSsPR |
| 1750 | Q5T4S7 | HVTLPSsPR |
| 1751 | Q5T4S7 | TGsTSSKEDDYESDAATIVQK |
| 1752 | Q5T3I0 | DLESCsDDDNQGSK |
| 1753 | Q5T3I0 | DLEsCsDDDNQGSK |
| 1754 | Q5T200 | EVsPEVVR |

| | | |
|---|---|---|
| 1755 | Q5T200 | GPRtPsPPPPIPEDIALGK |
| 1756 | Q5T200 | GPRtPsPPPPIPEDIALGK |
| 1757 | Q5T200 | sLsPSHLTEDR |
| 1758 | Q5T200 | SLsPSHLTEDR |
| 1759 | Q5T200 | STsPAGQHHsPISSR |
| 1760 | Q5T200 | STsPAGQHHsPISSR |
| 1761 | Q5T200 | SKLsPsPSLR |
| 1762 | Q5T200 | SKLsPSPSLR |
| 1763 | Q5T200 | GNIETTSEDGQVFsPK |
| 1764 | Q5T200 | GNIETTsEDGQVFSPK |
| 1765 | Q5T200 | sAsPYPSHSLSSPQR |
| 1766 | Q5T200 | SAsPYPSHSLSsPQR |
| 1767 | Q5T200 | sTSPAGQHHsPISSR |
| 1768 | Q5T1M5 | SSLsGDEEDELFK |
| 1769 | Q5T1M5 | RPsQEQSASASSGQPQAPLNR |
| 1770 | Q5T1M5 | EVAPDGPLQESSTRLsLTsDPEEGDPLALGPESPGEPQPPQLK |
| 1771 | Q5T1M5 | EVAPDGPLQESSTRLSLTSDPEEGDPLALGPEsPGEPQPPQLK |
| 1772 | Q5T1M5 | DSAAPsPIPGADNLSADPVVsPPTSIPFK |
| 1773 | Q5T0W9 | sFPLFDNSK |
| 1774 | Q5T0W9 | sFNGTDNHIR |
| 1775 | Q5T0W9 | TPsPGPVESK |
| 1776 | Q5T0N5 | TIsDGTISASK |
| 1777 | Q5T011 | AEGIEGETLTAsPQAPGsPEDSEGVPLISLPR |
| 1778 | Q5T011 | AEGIEGETLTAsPQAPGsPEDSEGVPLISLPR |
| 1779 | Q5SXM2 | VGsEsEDEDLLSELELADR |
| 1780 | Q5SXM2 | VGsEsEDEDLLSELELADR |
| 1781 | Q5SW79 | LGSLsARsDSEATISR |
| 1782 | Q5SW79 | LGsLSAR |
| 1783 | Q5QJE6 | ESYTEEIVsEAESHVsGISR |
| 1784 | Q5QJE6 | ESYTEEIVsEAEsHVSGISR |
| 1785 | Q5QJE6 | ESYTEEIVsEAESHVsGISR |
| 1786 | Q5M775 | KsVSSPTSSNTPTPTK |
| 1787 | Q5M775 | KSVssPTSSNTPTPTK |
| 1788 | Q5JTV8 | DSHSsEEDEASSQTDLSQTISK |
| 1789 | Q5JTV8 | DSHssEEDEASSQTDLSQTISK |
| 1790 | Q5JTV8 | DsHsSEEDEASSQTDLSQTISK |
| 1791 | Q5JTV8 | VNFSEEGEtEEDDQDSSHSSVTTVK |
| 1792 | Q5JTV8 | VNFsEEGETEEDDQDSSHSSVTTVK |
| 1793 | Q5JTH9 | GDSIEEILADsEDEEDNEEEER |
| 1794 | Q5JTD0 | GsPEEELPLPAFEK |
| 1795 | Q5JTD0 | KDsLTQAQEQGNLLN |

| | | |
|---|---|---|
| 1796 | Q5JSH3 | VGNEsPVQELK |
| 1797 | Q5JSH3 | EYVSNDAAQsDDEEK |
| 1798 | Q5JSH3 | ASEsDTEEFYDAPEDVHLGGGYPVGSPGK |
| 1799 | Q5JSH3 | AsEsDTEEFYDAPEDVHLGGGYPVGSPGK |
| 1800 | Q5JSH3 | ASEsDTEEFYDAPEDVHLGGGYPVGsPGK |
| 1801 | Q5HYJ3 | ISNLsPEEEQGLWK |
| 1802 | Q5HYI7 | LTPAEEENNSFQRLsP |
| 1803 | Q5H9R7 | IQQFDDGGsDEEDIWEEK |
| 1804 | Q5H9R7 | NTVDLVTTCHIHSssDDEIDFK |
| 1805 | Q5H9R7 | NTVDLVTTCHIHssSDDEIDFK |
| 1806 | Q58WW2 | ADRLEGDRsEGsGQENENEDEE |
| 1807 | Q58WW2 | ADRLEGDRsEGsGQENENEDEE |
| 1808 | Q58WW2 | DSALQDtDDsDDDPVLIPGAR |
| 1809 | Q58WW2 | DSALQDtDDsDDDPVLIPGAR |
| 1810 | Q56P03 | YYDDIYFDsDsEDEDR |
| 1811 | Q56P03 | YYDDIYFDsDsEDEDR |
| 1812 | Q53SF7 | DQTAsAPAtPLVNK |
| 1813 | Q53SF7 | DQTAsAPATPLVNK |
| 1814 | Q53LP3 | DLVMGSsPQLK |
| 1815 | Q53GS9 | EREVDEDsEPER |
| 1816 | Q53EZ4 | SPTAALNEsLVECPK |
| 1817 | Q53EZ4 | VAASPKsPtAALNEsLVECPK |
| 1818 | Q53EL6 | FVsEGDGGR |
| 1819 | Q53EL6 | DSGRGDsVSDSGSDALR |
| 1820 | Q53EL6 | SGLTVPTsPK |
| 1821 | Q53EL6 | SGLTVPtSPK |
| 1822 | Q52LW3 | SFENVsVEsVDSSSEK |
| 1823 | Q52LW3 | SFENVsVEsVDSSSEK |
| 1824 | Q4LE39 | DIEVLsEDtDYEEDEVTK |
| 1825 | Q4LE39 | DIEVLsEDtDYEEDEVTK |
| 1826 | Q4G0J3 | DIEIStEEEKDTGDLK |
| 1827 | Q4G0J3 | DIEIstEEEKDTGDLK |
| 1828 | Q4G0J3 | KRsSSEDAESLAPR |
| 1829 | Q4G0J3 | KRSssEDAESLAPR |
| 1830 | Q4ADV7 | AIGSGESEtPPSTPTAQEPSSSGGFEFFR |
| 1831 | Q4ADV7 | AIGSGESEtPPStPTAQEPSSSGGFEFFR |
| 1832 | Q3YEC7 | AAQQDsDsDGEALGGNPMVAGFQDDVDLEDQPR |
| 1833 | Q3YEC7 | AAQQDsDsDGEALGGNPMVAGFQDDVDLEDQPR |
| 1834 | Q3YEC7 | ADDFPVRDDPsDVtDEDEGPAEPPPPPK |
| 1835 | Q3YEC7 | ADDFPVRDDPsDVtDEDEGPAEPPPPPK |
| 1836 | Q3YEC7 | GsPPLPAGPVPSQDITLSSEEEAEVAAPTK |

| | | |
|---|---|---|
| 1837 | Q3YEC7 | GSPPLPAGPVPSQDITLssEEEAEVAAPTK |
| 1838 | Q3YEC7 | GSPPLPAGPVPSQDITLssEEEAEVAAPTK |
| 1839 | Q3MIN7 | VIEPPAAsCPSsPR |
| 1840 | Q3MIN7 | VIEPPAAsCPSsPR |
| 1841 | Q3L8U1 | DELAELsEAEsEGDEKPK |
| 1842 | Q3L8U1 | DELAELsEAEsEGDEKPK |
| 1843 | Q3L8U1 | TLIKsEPVsPK |
| 1844 | Q3L8U1 | TLIKsEPVsPK |
| 1845 | Q3KQU3 | ASNEKESAAPAsPAPsPAPSPTPAPPQK |
| 1846 | Q3KQU3 | ASNEKESAAPAsPAPsPAPSPTPAPPQK |
| 1847 | Q3KQU3 | ESAAPAsPAPSPAPsPtPAPPQK |
| 1848 | Q3KQU3 | RSSQPsPTAVPASDSPPTK |
| 1849 | Q3B726 | KHsEEAEFTPPLK |
| 1850 | Q3B726 | KHsEEAEFTPPLKCsPK |
| 1851 | Q32MZ4 | IDGATQSsPAEPK |
| 1852 | Q2VPK5 | DCLIEDsDDEAGQS |
| 1853 | Q2M2Z5 | RAsPPVsPIPVSEYCESENK |
| 1854 | Q2M2Z5 | RAsPPVsPIPVSEYCESENK |
| 1855 | Q2KHR3 | GQDTVAIEGFtDEEDTEsGGEGQYR |
| 1856 | Q2KHR3 | GQDTVAIEGFtDEEDTEsGGEGQYR |
| 1857 | Q29RF7 | AAVGQEsPGGLEAGNAK |
| 1858 | Q27J81 | DPTSLLGVLQAEADsTsEGLEDAVHSR |
| 1859 | Q27J81 | DPTSLLGVLQAEADsTsEGLEDAVHSR |
| 1860 | Q1KMD3 | EEDEPEERsGDETPGSEVPGDK |
| 1861 | Q1KMD3 | SGDEtPGsEVPGDK |
| 1862 | Q1KMD3 | REEDEPEERsGDEtPGSEVPGDK |
| 1863 | Q1ED39 | ESGVAGDPWKEETDtDLEVVLEK |
| 1864 | Q1ED39 | ESGVAGDPWKEEtDtDLEVVLEK |
| 1865 | Q16666 | EVDATsPAPSTSSTVK |
| 1866 | Q16643 | LSsPVLHR |
| 1867 | Q16637 | GTGQSDDsDIWDDTALIK |
| 1868 | Q16637 | GTGQsDDSDIWDDTALIK |
| 1869 | Q16629 | RsAsPERMD |
| 1870 | Q16629 | RsAsPERMD |
| 1871 | Q16629 | YFQsPsR |
| 1872 | Q16629 | YFQsPsR |
| 1873 | Q16555 | TVtPASSAKTsPAK |
| 1874 | Q16555 | TVTPASsAKTsPAK |
| 1875 | Q16555 | TVTPASsAKTsPAK |
| 1876 | Q16555 | GLYDGPVCEVSVtPK |
| 1877 | Q16543 | TGDEKDVsV |

| | | |
|---|---|---|
| 1878 | Q16514 | IPGTPGAGGRLsPENNQVLTK |
| 1879 | Q16512 | TDVSNFDEEFTGEAPTLsPPR |
| 1880 | Q16512 | LNLGTDSDSsPQK |
| 1881 | Q16204 | LDQPVsAPPsPR |
| 1882 | Q16204 | LDQPVSAPPsPR |
| 1883 | Q16204 | RsNsPDKFK |
| 1884 | Q15942 | sPGAPGPLTLK |
| 1885 | Q15811 | SAFTPATATGSSPsPVLGQGEK |
| 1886 | Q15773 | LAIQGPEDsPSR |
| 1887 | Q15751 | SESDLSQPEsDEEGYALSGR |
| 1888 | Q15742 | SFSPKSPLELGEKLsPLPGGPGAGDPR |
| 1889 | Q15742 | SFSPKsPLELGEKLsPLPGGPGAGDPR |
| 1890 | Q15742 | SFsPKsPLELGEK |
| 1891 | Q15696 | DIYLsPDR |
| 1892 | Q15648 | SQtPPGVATPPIPK |
| 1893 | Q15637 | TGDLGIPPNPEDRsPSPEPIyNSEGK |
| 1894 | Q15637 | SPsPEPIYNSEGK |
| 1895 | Q15527 | DLGSTEDGDGTDDFLtDKEDEK |
| 1896 | Q15527 | EAFWEPTSSDEGGAAsDDSMTDLYPPELFTR |
| 1897 | Q15459 | FGESEEVEMEVEsDEEDDKQEK |
| 1898 | Q15459 | EKQsDDEVYAPGLDIESSLK |
| 1899 | Q15424 | SVVsFDK |
| 1900 | Q15424 | sVVSFDK |
| 1901 | Q15424 | TDCEPVGLEPAVEQSSAASELAEASSEELAEAPTEAPsPEAR |
| 1902 | Q15424 | TDCEPVGLEPAVEQSSAASELAEASSEELAEAPtEAPsPEAR |
| 1903 | Q15424 | TDCEPVGLEPAVEQSSAASELAEAsSEELAEAPTEAPsPEAR |
| 1904 | Q15424 | TDCEPVGLEPAVEQSSAASELAEAssEELAEAPTEAPsPEAR |
| 1905 | Q15424 | AETLSGLGDSGAAGAAALsSASSETGTR |
| 1906 | Q15418 | tPKDSPGIPPsAGAHQLFR |
| 1907 | Q15418 | DsPGIPPsAGAHQLFR |
| 1908 | Q15418 | DsPGIPPsAGAHQLFR |
| 1909 | Q15361 | YLsADsGDADDSDADLGSAVK |
| 1910 | Q15361 | YLSADsGDADDSDADLGSAVK |
| 1911 | Q15311 | RTEGYAAFQEDssGDEAESPSK |
| 1912 | Q15311 | RTEGYAAFQEDssGDEAESPSK |
| 1913 | Q15276 | AQsTDSLGTSGSLQSK |
| 1914 | Q15276 | AQSTDsLGTSGSLQSK |
| 1915 | Q15185 | DWEDDsDEDMSNFDR |
| 1916 | Q15185 | FSEMMNNMGGDEDVDLPEVDGADDDsQDSDDEKMPDLE |
| 1917 | Q15185 | FSEMMNNMGGDEDVDLPEVDGADDDSQDsDDEKMPDLE |
| 1918 | Q15154 | VTNDIsPESSPGVGR |

| | | |
|---|---|---|
| 1919 | Q15154 | NVRsDIsDQEEDEESEGCPVSINLSK |
| 1920 | Q15154 | NVRsDIsDQEEDEESEGCPVSINLSK |
| 1921 | Q15154 | NVRsDIsDQEEDEEsEGCPVSINLSK |
| 1922 | Q15154 | VTNDIsPEsSPGVGR |
| 1923 | Q15154 | VTNDIsPESsPGVGR |
| 1924 | Q15149 | RAsFAEK |
| 1925 | Q15149 | AQLEPVAsPAK |
| 1926 | Q15149 | SDEGQLsPATR |
| 1927 | Q15149 | GYYSPYSVSGSGSTAGsR |
| 1928 | Q15149 | SSsVGSSSSYPISPAVSR |
| 1929 | Q15149 | GYYSPYSVSGSGsTAGsR |
| 1930 | Q15149 | SSsVGsSSSYPISPAVSR |
| 1931 | Q15149 | GYYSPYSVsGSGSTAGsR |
| 1932 | Q15121 | QPsEEEIIK |
| 1933 | Q15061 | LQAKEsPQR |
| 1934 | Q15059 | SEsPPPLSDPK |
| 1935 | Q15058 | AHEEQDEEsQDNLFSSDR |
| 1936 | Q15054 | VALsDDETK |
| 1937 | Q15036 | LSSKLsAVsLR |
| 1938 | Q15036 | LSSKLsAVsLR |
| 1939 | Q15029 | MDTDLYDEFGNYIGPELDsDEDDDELGR |
| 1940 | Q15025 | ALEEALSIQtPPSSPPTAFGsPEGAGALLR |
| 1941 | Q15025 | ALEEALSIQtPPSSPPTAFGsPEGAGALLR |
| 1942 | Q15021 | YQPLAsTAsDNDFVTPEPR |
| 1943 | Q15021 | YQPLASTAsDNDFVTPEPR |
| 1944 | Q15020 | ATAAETSAsEPEAESK |
| 1945 | Q15020 | ATAAETsASEPEAESK |
| 1946 | Q14CW9 | LQWDGSSDLsPSDSGSSK |
| 1947 | Q14C86 | FsLCsDNLEGISEGPSNR |
| 1948 | Q14C86 | FsLCSDNLEGISEGPSNR |
| 1949 | Q14C86 | SRsSDIVSSVR |
| 1950 | Q14980 | TQPDGTSVPGEPAsPISQR |
| 1951 | Q14980 | APVPSTCSSTFPEELsPPSHQAK |
| 1952 | Q14978 | VADNsFDAK |
| 1953 | Q14978 | IKLQtPNtFPK |
| 1954 | Q14978 | IKLQtPNtFPK |
| 1955 | Q14978 | GGSISVQVNSIKFDsE |
| 1956 | Q14966 | NPFRGsPK |
| 1957 | Q14839 | KMsQPGsPSPK |
| 1958 | Q14839 | KMsQPGsPSPK |
| 1959 | Q14839 | TPTPSTPGDTQPNtPAPVPPAEDGIK |

| | | |
|---|---|---|
| 1960 | Q14839 | TPTPSTPGDtQPNtPAPVPPAEDGIK |
| 1961 | Q14739 | SAsASHQADIK |
| 1962 | Q14738 | RKsELPQDVYTIK |
| 1963 | Q14694 | NHSVNEEEQEEQGEGsEDEWEQVGPR |
| 1964 | Q14694 | TCNSPQNSTDSVSDIVPDsPFPGALGSDTR |
| 1965 | Q14684 | VGDGDLsAEEIPENEVSLR |
| 1966 | Q14684 | GsPTGGAQLLK |
| 1967 | Q14684 | VFCVEEEDsESSLQK |
| 1968 | Q14684 | VFCVEEEDSEsSLQK |
| 1969 | Q14678 | SYsAGNASQLEQLSR |
| 1970 | Q14676 | AQPFGFIDSDtDAEEERIPAtPVVIPMK |
| 1971 | Q14676 | AQPFGFIDsDtDAEEER |
| 1972 | Q14676 | SQASMVINsDtDDEEEVSAALTLAHLK |
| 1973 | Q14676 | SQASMVINsDtDDEEEVSAALTLAHLK |
| 1974 | Q14676 | LLLAEDsEEEVDFLSER |
| 1975 | Q14676 | SQPPGEDsDtDVDDDSRPPGRPAEVHLER |
| 1976 | Q14676 | SQPPGEDsDtDVDDDSRPPGRPAEVHLER |
| 1977 | Q14676 | SQTTTERDsDtDVEEEELPVENR |
| 1978 | Q14676 | SQTTTERDsDtDVEEEELPVENR |
| 1979 | Q14676 | AQPFGFIDSDtDAEEERIPAtPVVIPMK |
| 1980 | Q14669 | VREDDEDsDDDGsDEEIDESLAAQFLNSGNVR |
| 1981 | Q14669 | VREDDEDsDDDGsDEEIDESLAAQFLNSGNVR |
| 1982 | Q14643 | RDsVLAASR |
| 1983 | Q14573 | LGFVDVQNCIsR |
| 1984 | Q14566 | EIESEIDsEEELINK |
| 1985 | Q14527 | KTAVQYIEssDSEEIEtSELPQK |
| 1986 | Q14527 | KTAVQYIEssDSEEIEtSELPQK |
| 1987 | Q14527 | KTAVQYIEssDsEEIETSELPQK |
| 1988 | Q14498 | DKsPVREPIDNLTPEER |
| 1989 | Q14432 | WVEDSDEsGDtDDPEEEEEEAPAPNEEETCENNESPK |
| 1990 | Q14432 | WVEDSDEsGDtDDPEEEEEEAPAPNEEETCENNESPK |
| 1991 | Q14432 | WVEDsDEsGDtDDPEEEEEEAPAPNEEETCENNESPK |
| 1992 | Q14315 | LGsFGSITR |
| 1993 | Q14258 | ASAPsPNAQVACDHCLK |
| 1994 | Q14247 | AKTQTPPVsPAPQPTEERLPSSPVYEDAASFK |
| 1995 | Q14247 | AKTQtPPVsPAPQPTEER |
| 1996 | Q14247 | AKtQTPPVsPAPQPTEER |
| 1997 | Q14247 | AKTQtPPVsPAPQPTEERLPSsPVYEDAASFK |
| 1998 | Q14247 | LPSsPVYEDAAsFK |
| 1999 | Q14247 | LPsSPVYEDAAsFK |
| 2000 | Q14204 | TDsTSDGRPAWMR |

| | | |
|---|---|---|
| 2001 | Q14181 | sPHQLLSPSSFsPSATPSQK |
| 2002 | Q14181 | sPHQLLSPSSFSPSATPSQK |
| 2003 | Q14160 | VQsPEPPAPER |
| 2004 | Q14160 | NsLESISSIDR |
| 2005 | Q14160 | RSEACPCQPDSGsPLPAEEEK |
| 2006 | Q14160 | AFAAVPTSHPPEDAPAQPPtPGPAASPEQLsFR |
| 2007 | Q14160 | AFAAVPTSHPPEDAPAQPPtPGPAAsPEQLSFR |
| 2008 | Q14160 | MAESPCSPSGQQPPsPPsPDELPANVK |
| 2009 | Q14160 | MAESPCsPSGQQPPSPPsPDELPANVK |
| 2010 | Q14160 | MAESPCSPSGQQPPsPPsPDELPANVK |
| 2011 | Q14160 | LALDGETLGEEEQEDEQPPWASPsPTSR |
| 2012 | Q14160 | LALDGETLGEEEQEDEQPPWAsPsPTSR |
| 2013 | Q14157 | STSAPQMsPGSSDNQSSSPQPAQQK |
| 2014 | Q14157 | RYPSSISSsPQK |
| 2015 | Q14157 | RYPSsISSsPQK |
| 2016 | Q14155 | MsGFIYQGK |
| 2017 | Q14155 | KPsDEEFASR |
| 2018 | Q14152 | LGDSsLSR |
| 2019 | Q14147 | LQEEQDGGssDEDR |
| 2020 | Q14147 | LQEEQDGGssDEDR |
| 2021 | Q14137 | IGDEYAEDssDEEDIR |
| 2022 | Q14137 | IGDEYAEDssDEEDIR |
| 2023 | Q14134 | DARsPSGPSGSLENGTK |
| 2024 | Q14103 | IDASKNEEDEGHSNSsPR |
| 2025 | Q14103 | IDASKNEEDEGHsNSSPR |
| 2026 | Q14004 | RRsLsPLGGR |
| 2027 | Q14004 | RRsLsPLGGR |
| 2028 | Q14004 | GGDVsPSPYSSSSWR |
| 2029 | Q14004 | SLsPLGGRDDsPVSHR |
| 2030 | Q14004 | HSsIsPSTLTLK |
| 2031 | Q14004 | HSsIsPSTLTLK |
| 2032 | Q14004 | KsPsPAGGGSSPYSR |
| 2033 | Q14004 | SRKSPsPAGGGSsPYSR |
| 2034 | Q13895 | MPQDGsDDEDEEWPTLEK |
| 2035 | Q13769 | ALFKPPEDSQDDEsDsDAEEEQTTK |
| 2036 | Q13769 | ALFKPPEDSQDDEsDsDAEEEQTTK |
| 2037 | Q13614 | SASVVSSDSISTSADNFsPDLR |
| 2038 | Q13613 | GSsPSHSATSVHTSV |
| 2039 | Q13610 | EKLQEEGGGsDEEETGSPSEDGMQSAR |
| 2040 | Q13610 | EKLQEEGGGSDEEEtGsPSEDGMQSAR |
| 2041 | Q13601 | AsPSLERPEK |

| | | |
|---|---|---|
| 2042 | Q13596 | LPPPFPGLEPESEGAAGGsEPEAGDSDtEGEDIFTGAAVVSK |
| 2043 | Q13596 | LPPPFPGLEPESEGAAGGSEPEAGDsDtEGEDIFTGAAVVSK |
| 2044 | Q13596 | LPPPFPGLEPESEGAAGGSEPEAGDsDtEGEDIFTGAAVVSK |
| 2045 | Q13586 | LPDsPALAK |
| 2046 | Q13573 | GPPsPPAPVMHSPSR |
| 2047 | Q13573 | GPPsPPAPVMHsPSR |
| 2048 | Q13547 | IACEEEFsDsEEEGEGGR |
| 2049 | Q13547 | IACEEEFsDsEEEGEGGR |
| 2050 | Q13547 | MLPHAPGVQMQAIPEDAIPEEsGDEDEDDPDKR |
| 2051 | Q13542 | TVAISDAAQLPHDYCTTPGGTLFSTtPGGTR |
| 2052 | Q13542 | TVAISDAAQLPHDYCTtPGGTLFSTtPGGTR |
| 2053 | Q13541 | RVVLGDGVQLPPGDYSTtPGGTLFSTtPGGTR |
| 2054 | Q13541 | RVVLGDGVQLPPGDYSTTPGGTLFSTtPGGTR |
| 2055 | Q13541 | FLMECRNsPVTK |
| 2056 | Q13541 | FLMECRNsPVTKtPPR |
| 2057 | Q13523 | sRsPLLNDR |
| 2058 | Q13523 | sRsPLLNDR |
| 2059 | Q13523 | sLsPKPR |
| 2060 | Q13523 | sLsPKPR |
| 2061 | Q13523 | sRsPVDLR |
| 2062 | Q13523 | sRsPVDLR |
| 2063 | Q13523 | KKsPIINESR |
| 2064 | Q13523 | DAsPINRWsPTR |
| 2065 | Q13523 | DAsPINRWsPTR |
| 2066 | Q13523 | VQSGMGLILQGYEsGsEEEGEIHEK |
| 2067 | Q13523 | VQSGMGLILQGYEsGsEEEGEIHEK |
| 2068 | Q13523 | TRsPsPDDILER |
| 2069 | Q13523 | TRsPsPDDILER |
| 2070 | Q13523 | ERsKDAsPINR |
| 2071 | Q13501 | LTPVsPESSSTEEK |
| 2072 | Q13501 | LtPVsPESSSTEEK |
| 2073 | Q13501 | EVDPSTGELQsLQMPESEGPSSLDPSQEGPTGLK |
| 2074 | Q13501 | EVDPSTGELQSLQMPESEGPSsLDPSQEGPTGLK |
| 2075 | Q13501 | EVDPSTGELQSLQMPEsEGPSSLDPSQEGPTGLK |
| 2076 | Q13501 | EVDPSTGELQSLQMPESEGPsSLDPSQEGPTGLK |
| 2077 | Q13470 | sLESVLSLGPRPTGGGSsPPEIR |
| 2078 | Q13470 | sLESVLSLGPRPTGGGsSPPEIR |
| 2079 | Q13459 | VSPPAPGsAPETPEDK |
| 2080 | Q13459 | VSPPAPGsAPEtPEDK |
| 2081 | Q13459 | KKPGDASSLPDAGLsPGSQVDSK |
| 2082 | Q13459 | VQEKPDsPGGSTQIQR |

| | | |
|---|---|---|
| 2083 | Q13459 | SPLEHSsPEKEAPsPEK |
| 2084 | Q13459 | SPLEHsSPEKEAPsPEK |
| 2085 | Q13443 | HVsPVtPPR |
| 2086 | Q13443 | HVsPVtPPR |
| 2087 | Q13442 | KSLDsDEsEDEEDDYQQK |
| 2088 | Q13442 | KSLDsDEsEDEEDDYQQK |
| 2089 | Q13442 | KsLDSDEsEDEEDDYQQK |
| 2090 | Q13439 | EENPEsDGEPVVEDGTSVK |
| 2091 | Q13439 | TSsFTEQLDEGTPNR |
| 2092 | Q13435 | GFEEEHKDsDDDssDDEQEKKPEAPK |
| 2093 | Q13435 | GFEEEHKDsDDDssDDEQEKKPEAPK |
| 2094 | Q13435 | GFEEEHKDsDDDssDDEQEKKPEAPK |
| 2095 | Q13435 | SSLGQsASETEEDTVSVSK |
| 2096 | Q13435 | SSLGQSAsETEEDTVSVSK |
| 2097 | Q13435 | SSLGQSAsEtEEDTVSVSK |
| 2098 | Q13428 | AALAPAKEsPR |
| 2099 | Q13428 | KLGAGEGGEASVsPEK |
| 2100 | Q13428 | KLGAGEGGEAsVsPEK |
| 2101 | Q13428 | TSQVGAASAPAKEsPR |
| 2102 | Q13428 | AASAPAKEsPR |
| 2103 | Q13428 | TETLVEETAAEssEDDVVAPSQSLLSGYMTPGLTPANSQASK |
| 2104 | Q13428 | TETLVEEtAAESSEDDVVAPSQSLLSGYMTPGLTPANSQASK |
| 2105 | Q13427 | RsEtPPHWR |
| 2106 | Q13427 | RsEtPPHWR |
| 2107 | Q13427 | ADRDQsPFSK |
| 2108 | Q13427 | FDHESsPGtDEDKSG |
| 2109 | Q13427 | FDHESsPGtDEDKSG |
| 2110 | Q13426 | QEGETAICSEMTADRDPVYDEstDEESENQTDLSGLASAAVSK |
| 2111 | Q13426 | QEGETAICSEMTADRDPVYDEstDEESENQTDLSGLASAAVSK |
| 2112 | Q13425 | GPAGEAGAsPPVR |
| 2113 | Q13425 | sPSLGSDLTFATR |
| 2114 | Q13404 | LPQPPEGQCYsN |
| 2115 | Q13371 | NSATCHSEDsDLEID |
| 2116 | Q13286 | RFsDSEGEETVPEPR |
| 2117 | Q13286 | RFSDsEGEETVPEPR |
| 2118 | Q13283 | YQDEVFGGFVTEPQEEsEEEVEEPEER |
| 2119 | Q13283 | SSsPAPADIAQTVQEDLR |
| 2120 | Q13263 | AASAAAASAAAASAASGsPGPGEGSAGGEK |
| 2121 | Q13263 | LDLDLTADsQPPVFK |
| 2122 | Q13263 | LASPSGSTSsGLEVVAPEGTSAPGGGPGTLDDSATICR |
| 2123 | Q13263 | LAsPSGsTSSGLEVVAPEGTSAPGGGPGTLDDSATICR |

| | | |
|---|---|---|
| 2124 | Q13263 | LAsPSGSTSSGLEVVAPEGTSAPGGGPGTLDDSATICR |
| 2125 | Q13247 | ARsVsPPPK |
| 2126 | Q13247 | ARsVsPPPK |
| 2127 | Q13247 | SNsPLPVPPSK |
| 2128 | Q13243 | sVsRsPVPEK |
| 2129 | Q13243 | sVSRsPVPEK |
| 2130 | Q13243 | sVSRsPVPEK |
| 2131 | Q13242 | GsPHYFSPFRPY |
| 2132 | Q13242 | GsPHYFSPFRPy |
| 2133 | Q13242 | STsYGYSR |
| 2134 | Q13242 | GsPHYFsPFRPY |
| 2135 | Q13200 | APVQPQQsPAAAPGGTDEKPSGK |
| 2136 | Q13185 | KSLsDSESDDSK |
| 2137 | Q13177 | YLsFTPPEK |
| 2138 | Q13131 | SIDDEITEAKSGtAtPQR |
| 2139 | Q13131 | SIDDEITEAKsGTAtPQR |
| 2140 | Q13112 | TDTPPSSVPTSVISTPSTEEIQSETPGDAQGsPPELK |
| 2141 | Q13112 | GSsPGPRPVEGTPASR |
| 2142 | Q13112 | TQDPSsPGTtPPQAR |
| 2143 | Q13112 | TQDPSsPGTTPPQAR |
| 2144 | Q13111 | sCPELTSGPR |
| 2145 | Q13111 | sPSTTYLHTPTPSEDAAIPSK |
| 2146 | Q13098 | EGSQGELtPANSQSR |
| 2147 | Q13098 | EGsQGELTPANSQSR |
| 2148 | Q13085 | FIIGSVSEDNsEDEISNLVK |
| 2149 | Q13085 | FIIGsVSEDNSEDEISNLVK |
| 2150 | Q13085 | SSMsGLHLVK |
| 2151 | Q13085 | FIIGSVsEDNSEDEISNLVK |
| 2152 | Q13045 | NAEAVLQsPGLSGK |
| 2153 | Q13033 | SSGDVLETFNFLENADDsDEDEENDMIEGIPEGK |
| 2154 | Q12996 | RPNEDsDEDEEK |
| 2155 | Q12982 | KGsITEYTAAEEK |
| 2156 | Q12965 | NQYVPYPHAPGsQR |
| 2157 | Q12948 | TENGTCPsPPQPLsPAAALGSGSAAAVPK |
| 2158 | Q12948 | TENGTCPsPPQPLsPAAALGSGSAAAVPK |
| 2159 | Q12933 | AAASVtPPGsLELLQPGFSK |
| 2160 | Q12933 | AAASVtPPGsLELLQPGFSK |
| 2161 | Q12929 | ISAAASDSGVEsFDEGSSH |
| 2162 | Q12929 | ISAAASDsGVESFDEGSSH |
| 2163 | Q12929 | DSVSsVSDISQYR |
| 2164 | Q12906 | RPMEEDGEEKsPSK |

| | | |
|---|---|---|
| 2165 | Q12906 | DSSKGEDsAEETEAKPAVVAPAPVVEAVSTPSAAFPSDATAEQGPILTK |
| 2166 | Q12906 | DSSKGEDSAEETEAKPAVVAPAPVVEAVsTPSAAFPSDATAEQGPILTK |
| 2167 | Q12906 | DSSKGEDSAEETEAKPAVVAPAPVVEAVStPSAAFPSDATAEQGPILTK |
| 2168 | Q12888 | GGPGKLsPR |
| 2169 | Q12888 | NsPEDLGLSLTGDSCK |
| 2170 | Q12888 | ECSEAMEVETSVISIDsPQK |
| 2171 | Q12888 | ETAVPGPLGIEDIsPNLSPDDK |
| 2172 | Q12888 | ETAVPGPLGIEDISPNLsPDDK |
| 2173 | Q12888 | TSsGTSLSAMHSSGSSGK |
| 2174 | Q12888 | LPDGPTGssEEEEEFLEIPPFNK |
| 2175 | Q12888 | LPDGPTGssEEEEEFLEIPPFNK |
| 2176 | Q12874 | EEEEEEQIsEsEsEDEENEIIYNPK |
| 2177 | Q12874 | EEEEEEQIsEsEsEDEENEIIYNPK |
| 2178 | Q12874 | EEEEEEQIsEsEsEDEENEIIYNPK |
| 2179 | Q12873 | METEADAPsPAPSLGER |
| 2180 | Q12873 | METEADAPsPAPsLGER |
| 2181 | Q12873 | ELQGDGPPSSPtNDPTVK |
| 2182 | Q12872 | GVsQEKEAQISSAIVSSVQSK |
| 2183 | Q12857 | LKsVEDEMDsPGEEPFYTGQGR |
| 2184 | Q12857 | LKsVEDEMDsPGEEPFYTGQGR |
| 2185 | Q12802 | sQQSVSLSK |
| 2186 | Q12802 | VDSLVsLsEEDLESDQR |
| 2187 | Q12802 | VDSLVSLsEEDLESDQR |
| 2188 | Q12802 | ALQLSNsPGASSAFLK |
| 2189 | Q12802 | SGSLDSELSVsPK |
| 2190 | Q12802 | VDsLVSLsEEDLESDQR |
| 2191 | Q12802 | DMAECStPLPEDCSPTHSPR |
| 2192 | Q12802 | DMAECSTPLPEDCsPTHSPR |
| 2193 | Q12789 | DGsLEDDEDEEDDLDEGVGGK |
| 2194 | Q12789 | KNSSTDQGsDEEGSLQK |
| 2195 | Q12789 | KNsSTDQGsDEEGSLQK |
| 2196 | Q12774 | AEELsPAALSPsLEPIR |
| 2197 | Q12774 | AEELsPAALsPSLEPIR |
| 2198 | Q0JRZ9 | HsPVQMNR |
| 2199 | Q09666 | VDVKsPK |
| 2200 | Q09666 | IsAPNVDFNLEGPK |
| 2201 | Q09666 | AEAPLPsPK |
| 2202 | Q09666 | VDIDtPDIDIHGPEGK |
| 2203 | Q09666 | LKsEDGVEGDLGETQSR |
| 2204 | Q09666 | HRsNsFSDER |
| 2205 | Q09666 | HRsNsFSDER |

| | | |
|---|---|---|
| 2206 | Q09666 | GDRsPEPGQTWTR |
| 2207 | Q09666 | GGVTGsPEASISGSK |
| 2208 | Q09666 | FGTFGGLGsK |
| 2209 | Q09666 | ISMPDLDLHLKsPK |
| 2210 | Q09666 | VDIDtPDINIEGSEGK |
| 2211 | Q09666 | GHYEVTGsDDETGK |
| 2212 | Q09666 | VSMPDVELNLKsPK |
| 2213 | Q09666 | DDGVFVQEVTQNsPAAR |
| 2214 | Q09666 | LPsGSGAASPTGSAVDIR |
| 2215 | Q09666 | ASLGSLEGEAEAEASsPK |
| 2216 | Q09666 | ASLGsLEGEAEAEASSPK |
| 2217 | Q09666 | GGVTGsPEAsISGSK |
| 2218 | Q09666 | LPSGSGAAsPTGSAVDIR |
| 2219 | Q09666 | GPSLDIDtPDVNIEGPEGK |
| 2220 | Q09666 | DIDISsPEFK |
| 2221 | Q09666 | GPAFNMAsPESDFGINLK |
| 2222 | Q09666 | LPSGSGAASPTGsAVDIR |
| 2223 | Q09666 | VSVGAPDLSLEASEGsIK |
| 2224 | Q09161 | KTsDANETEDHLESLICK |
| 2225 | Q08J23 | AGEPNsPDAEEANSPDVTAGCDPAGVHPPR |
| 2226 | Q08J23 | AGEPNSPDAEEANsPDVTAGCDPAGVHPPR |
| 2227 | Q08J23 | ESTQLsPADLTEGKPTDPSK |
| 2228 | Q08AE8 | APTLAELDssEsEETLHK |
| 2229 | Q08AD1 | LNQSsPDNVTDTK |
| 2230 | Q08AD1 | SIsNEGLTLNNSHVSK |
| 2231 | Q08945 | EGMNPSYDEYADsDEDQHDAYLER |
| 2232 | Q08752 | DGsGDSHPDFPEDADIDLKDVDK |
| 2233 | Q08499 | SDSDYDLsPK |
| 2234 | Q08495 | STsPPPsPEVWADSR |
| 2235 | Q07960 | SDDSKSssPELVTHLK |
| 2236 | Q07955 | VDGPRsPsYGR |
| 2237 | Q07955 | VDGPRsPSYGR |
| 2238 | Q07157 | sVASSQPAKPTK |
| 2239 | Q07157 | sVAsSQPAKPTK |
| 2240 | Q07157 | KVQIPVSRPDPEPVsDNEEDsYDEEIHDPR |
| 2241 | Q07157 | KVQIPVSRPDPEPVsDNEEDsYDEEIHDPR |
| 2242 | Q07002 | DPPQECSTFSPTDSGEEPGQLsPGVQFQR |
| 2243 | Q07002 | RAsLSDIGFGK |
| 2244 | Q06210 | VDsTTCLFPVEEK |
| 2245 | Q05655 | AstFCGTPDYIAPEILQGLK |
| 2246 | Q05655 | AstFCGTPDYIAPEILQGLK |

| | | |
|---|---|---|
| 2247 | Q05519 | KPIETGsPK |
| 2248 | Q05519 | DYDEEEQGYDsEK |
| 2249 | Q05209 | TPLSFTNPLHsDDsDSDER |
| 2250 | Q05209 | TPLSFTNPLHsDDsDSDER |
| 2251 | Q05209 | DVDVSEDsPPPLPER |
| 2252 | Q04726 | VsPAHsPPENGLDK |
| 2253 | Q04726 | VsPAHsPPENGLDK |
| 2254 | Q04726 | ERESSANNSVsPSESLR |
| 2255 | Q04726 | DAPTsPASVASSSSTPSSK |
| 2256 | Q04726 | YDSDGDKSDDLVVDVSNEDPAtPR |
| 2257 | Q04726 | YDSDGDKsDDLVVDVSNEDPAtPR |
| 2258 | Q04726 | yDSDGDKsDDLVVDVSNEDPATPR |
| 2259 | Q04695 | LsGGLGAGSCR |
| 2260 | Q04695 | LSGGLGAGsCR |
| 2261 | Q04695 | QFTSSsSIK |
| 2262 | Q04695 | QFTSSSsIK |
| 2263 | Q04637 | EAALPPVsPLK |
| 2264 | Q04637 | RsFsKEVEER |
| 2265 | Q04637 | RSFsKEVEER |
| 2266 | Q04637 | AAsLTEDR |
| 2267 | Q03468 | LEDDsEEsDAEFDEGFK |
| 2268 | Q03468 | LEDDsEEsDAEFDEGFK |
| 2269 | Q03468 | KVPVQEIDDDFFPssGEEAEAASVGEGGGGGR |
| 2270 | Q03468 | KVPVQEIDDDFFPssGEEAEAASVGEGGGGGR |
| 2271 | Q03252 | AGGPAtPLsPTR |
| 2272 | Q03252 | AGGPAtPLsPTR |
| 2273 | Q03164 | AVFGESGGGGGSGEDEQFLGFGsDEEVR |
| 2274 | Q03164 | ALSSAVQASPTsPGGSPSSPSSGQR |
| 2275 | Q03164 | GPGEPDsPTPLHPPtPPILSTDR |
| 2276 | Q03164 | GPGEPDsPTPLHPPTPPILSTDR |
| 2277 | Q03111 | ALEVEEsNsEDEASFK |
| 2278 | Q03111 | ALEVEEsNsEDEASFK |
| 2279 | Q02952 | EGVTPWAsFK |
| 2280 | Q02952 | EGVtPWAsFKK |
| 2281 | Q02952 | SAEsPTSPVTSETGSTFK |
| 2282 | Q02952 | SAESPTsPVTSETGSTFK |
| 2283 | Q02880 | AsGsENEGDYNPGR |
| 2284 | Q02880 | AsGsENEGDYNPGR |
| 2285 | Q02880 | YFAEsDEEEDDVDFAMFN |
| 2286 | Q02880 | KVVEAVNsDsDSEFGIPK |
| 2287 | Q02880 | KVVEAVNsDsDSEFGIPK |

| | | |
|---|---|---|
| 2288 | Q02880 | KTSFDQDsDVDIFPSDFPTEPPSLPR |
| 2289 | Q02880 | YTFDFsEEEDDDADDDDDDNNDLEELK |
| 2290 | Q02880 | FDsNEEDSAsVFSPSFGLK |
| 2291 | Q02880 | FDsNEEDSASVFSPSFGLK |
| 2292 | Q02880 | ASPITNDGEDEFVPsDGLDKDEYTFsPGK |
| 2293 | Q02880 | ASPITNDGEDEFVPsDGLDKDEYTFSPGK |
| 2294 | Q02880 | AsPITNDGEDEFVPsDGLDKDEYTFsPGK |
| 2295 | Q02543 | SSGEIVYCGQVFEKsPLR |
| 2296 | Q02447 | IGPPsPGDDEEEAAAAAGAPAAAGATGDLASAQLGGAPNR |
| 2297 | Q02241 | SSTVAPAQPDGAEsEWtDVETR |
| 2298 | Q02241 | SSTVAPAQPDGAEsEWtDVETR |
| 2299 | Q02078 | GCDsPDPDTSYVLTPHTEEK |
| 2300 | Q02078 | GCDsPDPDTSYVLtPHTEEK |
| 2301 | Q01970 | sLGDEGLNR |
| 2302 | Q01850 | DLTGVNAQSEPVASGWELASVNPEPVSsPTtPPEYK |
| 2303 | Q01850 | DLTGVNAQSEPVASGWELASVNPEPVSsPTtPPEYK |
| 2304 | Q01831 | SEAAAPHTDAGGGLssDEEEGTSSQAEAAR |
| 2305 | Q01831 | SEAAAPHTDAGGGLssDEEEGTSSQAEAAR |
| 2306 | Q01813 | sFAGNLNTYK |
| 2307 | Q01804 | EEssEDENEVSNILR |
| 2308 | Q01804 | EEssEDENEVSNILR |
| 2309 | Q01433 | sLPGPAPCLK |
| 2310 | Q01167 | EGsPAPLEPEPGAAQPK |
| 2311 | Q01130 | sRsPPPVSK |
| 2312 | Q01130 | sRsPPPVSK |
| 2313 | Q01105 | RQsPLPPQK |
| 2314 | Q01082 | GEQVSQNGLPAEQGsPR |
| 2315 | Q01082 | ESsPIPsPTSDR |
| 2316 | Q01082 | ESsPIPsPTSDR |
| 2317 | Q01082 | TSsKESsPIPsPTSDR |
| 2318 | Q00839 | SSsPVNVK |
| 2319 | Q00839 | SsSPVNVK |
| 2320 | Q00613 | VKEEPPsPPQSPR |
| 2321 | Q00613 | VKEEPPsPPQsPR |
| 2322 | Q00536 | RVsLSEIGFGK |
| 2323 | Q00341 | VATLNsEEESDPPTYK |
| 2324 | P98175 | GLVAAYSGEsDsEEEQER |
| 2325 | P98175 | GLVAAYSGEsDSEEEQER |
| 2326 | P98175 | GLVAAYsGEsDsEEEQER |
| 2327 | P98175 | LASDDRPsPPR |
| 2328 | P98174 | EDEDtPPNsPNVDLGK |

| | | |
|---|---|---|
| 2329 | P98174 | EDEDtPPNsPNVDLGK |
| 2330 | P98082 | SSPNPFVGsPPK |
| 2331 | P85037 | SGGLQtPECLsR |
| 2332 | P85037 | SGGLQTPECLsR |
| 2333 | P85037 | EGsPIPHDPEFGSK |
| 2334 | P85037 | EEAPASPLRPLYPQIsPLK |
| 2335 | P85037 | EEAPAsPLRPLYPQIsPLK |
| 2336 | P85037 | sAPASPTHPGLMSPR |
| 2337 | P85037 | SAPAsPTHPGLMsPR |
| 2338 | P85037 | SAPAsPTHPGLMsPR |
| 2339 | P83369 | SAGAGsPARPPsPR |
| 2340 | P83369 | SAGAGsPARPPsPR |
| 2341 | P82094 | SVSEINsDDELSGK |
| 2342 | P82094 | sVSEINSDDELsGK |
| 2343 | P82094 | SNTEPQSPPIAsPK |
| 2344 | P80723 | AEGAATEEEGtPK |
| 2345 | P78362 | TVsASsTGDLPK |
| 2346 | P78362 | TVsASsTGDLPK |
| 2347 | P78356 | AEDEECENDGVGGNLLCSYGtPPDsPGNLLSFPR |
| 2348 | P78356 | AEDEECENDGVGGNLLCSYGtPPDsPGNLLSFPR |
| 2349 | P78346 | KPRPsEGDEDCLPASK |
| 2350 | P78332 | EKLQSFDsPER |
| 2351 | P78332 | EGETQGVAFEHESPADFQNSQsPVQDQDK |
| 2352 | P78316 | HNDIVDsDsDAEDR |
| 2353 | P78316 | HNDIVDsDsDAEDR |
| 2354 | P68363 | DYEEVGVDsVEGEGEEEGEEY |
| 2355 | P67809 | sVGDGETVEFDVVEGEK |
| 2356 | P67809 | AADPPAENSsAPEAEQGGAE |
| 2357 | P67809 | NEGSEsAPEGQAQQR |
| 2358 | P67809 | NYQQNYQNsESGEK |
| 2359 | P63208 | GKtPEEIR |
| 2360 | P63165 | sDQEAKPSTEDLGDK |
| 2361 | P62995 | RRsPsPYYSR |
| 2362 | P62995 | RRsPsPYYSR |
| 2363 | P62995 | sDSGEQNYGER |
| 2364 | P62888 | sLESINSR |
| 2365 | P62753 | LSsLRAsTSK |
| 2366 | P62753 | LSsLRAStSKsESSQK |
| 2367 | P62753 | LssLRAsTSK |
| 2368 | P62070 | FQEQECPPsPEPTR |
| 2369 | P61981 | DNLTLWTsDQQDDDGGEGNN |

| | | |
|---|---|---|
| 2370 | P61978 | DYDDMsPR |
| 2371 | P61247 | ADGYEPPVQEsV |
| 2372 | P60981 | AsGVQVADEVCR |
| 2373 | P60468 | PGPTPSGTNVGSSGRsPSK |
| 2374 | P58107 | QVsASELHTSGILGPETLR |
| 2375 | P56524 | QEPIEsDEEEAEPPREVEPGQR |
| 2376 | P56211 | YFDsGDYNMAK |
| 2377 | P55884 | ALENGDADEPSFsDPEDFVDDVsEEELLGDVLK |
| 2378 | P55884 | ALENGDADEPSFsDPEDFVDDVsEEELLGDVLK |
| 2379 | P55884 | ALENGDADEPsFSDPEDFVDDVsEEELLGDVLK |
| 2380 | P55884 | TEPAAEAEAASGPSEsPsPPAAEELPGSHAEPPVPAQGEAPGEQAR |
| 2381 | P55884 | TEPAAEAEAASGPSEsPSPPAAEELPGSHAEPPVPAQGEAPGEQAR |
| 2382 | P55884 | TEPAAEAEAASGPsESPsPPAAEELPGSHAEPPVPAQGEAPGEQAR |
| 2383 | P55327 | NSPTFKsFEEK |
| 2384 | P55327 | KLEDVKNsPTFK |
| 2385 | P55210 | ADDQGCIEEQGVEDsANEDSVDAKPDR |
| 2386 | P55197 | GSLsPRsPVSSLQIR |
| 2387 | P55197 | GSLsPRsPVSSLQIR |
| 2388 | P55196 | SSPNVANQPPsPGGK |
| 2389 | P55196 | LFsQGQDVSNK |
| 2390 | P55081 | EDssEEEEEEIDDEEIER |
| 2391 | P55081 | EDssEEEEEEIDDEEIER |
| 2392 | P55081 | IVEPEVVGEsDsEVEGDAWR |
| 2393 | P55081 | IVEPEVVGEsDsEVEGDAWR |
| 2394 | P55081 | RPDYAPMEssDEEDEEFQFIK |
| 2395 | P55081 | RPDYAPMEssDEEDEEFQFIK |
| 2396 | P55010 | EAEEEssGGEEEDEDENIEVVYSK |
| 2397 | P55010 | EAEEEssGGEEEDEDENIEVVYSK |
| 2398 | P54725 | EDKsPSEESAPTTSPESVSGSVPSSGSSGR |
| 2399 | P54274 | AEDVSSAAPsPR |
| 2400 | P54274 | AEDVSsAAPsPR |
| 2401 | P54259 | TEQELPRPQsPSDLDsLDGR |
| 2402 | P54259 | TEQELPRPQsPSDLDSLDGR |
| 2403 | P54259 | SEEIsESESEETNAPK |
| 2404 | P54259 | SEEIsEsESEETNAPK |
| 2405 | P54105 | EPVADEEEEDsDDDVEPITEFR |
| 2406 | P53999 | EQIsDIDDAVR |
| 2407 | P53985 | KEsKEEETSIDVAGKPNEVTK |
| 2408 | P53621 | DADsQNPDAPEGK |
| 2409 | P53621 | NLsPGAVESDVR |
| 2410 | P53611 | GtPQKDVIIK |

| | | |
|---|---|---|
| 2411 | P53396 | TAsFSESR |
| 2412 | P52948 | NLNNSNLFsPVNR |
| 2413 | P52948 | NLNNsNLFsPVNR |
| 2414 | P52948 | YGLQDsDEEEEEHPSK |
| 2415 | P52948 | DSENLAsPSEYPENGER |
| 2416 | P52907 | ADFDDRVsDEEK |
| 2417 | P52756 | GLVAAYSGDsDNEEELVER |
| 2418 | P52756 | GLVAAYsGDsDNEEELVER |
| 2419 | P52701 | IHNVGsPLK |
| 2420 | P52701 | SEEDNEIEsEEEVQPK |
| 2421 | P52701 | RVIsDSESDIGGsDVEFKPDTK |
| 2422 | P52701 | VIsDsESDIGGSDVEFKPDTK |
| 2423 | P52701 | VHVQFFDDsPTR |
| 2424 | P52655 | DGAEDGQVEEEPLNsEDDVSDEEGQELFDtENVVVCQYDK |
| 2425 | P52655 | DGAEDGQVEEEPLNsEDDVsDEEGQELFDTENVVVCQYDK |
| 2426 | P52594 | GTPSQsPVVGR |
| 2427 | P52594 | GtPSQsPVVGR |
| 2428 | P51946 | HEEEEWtDDDLVESL |
| 2429 | P51858 | AGDLLEDsPK |
| 2430 | P51858 | GNAEGsSDEEGKLVIDEPAK |
| 2431 | P51858 | GNAEGSsDEEGKLVIDEPAK |
| 2432 | P51825 | ELsPLISLPsPVPPLsPIHSNQQTLPR |
| 2433 | P51825 | ELsPLISLPsPVPPLsPIHSNQQTLPR |
| 2434 | P51825 | ELsPLISLPsPVPPLsPIHSNQQTLPR |
| 2435 | P51812 | DsPGIPPsANAHQLFR |
| 2436 | P51812 | tPKDsPGIPPSANAHQLFR |
| 2437 | P51812 | DsPGIPPsANAHQLFR |
| 2438 | P51812 | NQsPVLEPVGR |
| 2439 | P51784 | LLSPAGSSGAPAsPACSSPPSSEFMDVN |
| 2440 | P51784 | LLSPAGSSGAPAsPACsSPPSSEFMDVN |
| 2441 | P51532 | IPDPDsDDVsEVDAR |
| 2442 | P51532 | AENAEGQTPAIGPDGEPLDETsQMsDLPVK |
| 2443 | P51532 | AENAEGQTPAIGPDGEPLDETsQMsDLPVK |
| 2444 | P51531 | IAKEEEsEDEsNEEEEEEDEEESESEAK |
| 2445 | P51531 | IAKEEEsEDEsNEEEEEEDEEESESEAK |
| 2446 | P51531 | IAKEEEsEDEsNEEEEEEDEEEsESEAK |
| 2447 | P51116 | TDGsIsGDRQPVTVADYISR |
| 2448 | P51116 | TDGsIsGDRQPVTVADYISR |
| 2449 | P51114 | RGPNYTSGYGTNSELsNPSETESER |
| 2450 | P51114 | RGPNYTSGYGTNSELSNPsETESER |
| 2451 | P50914 | AALLKAsPK |

| | | |
|---|---|---|
| 2452 | P50548 | GEGPGEAGGPLtPR |
| 2453 | P50548 | GsVSDCSDGTSELEEPLGEDPR |
| 2454 | P50502 | ADEPssEEsDLEIDK |
| 2455 | P50502 | ADEPSsEEsDLEIDK |
| 2456 | P50502 | ADEPSSEEsDLEIDK |
| 2457 | P49959 | GVDFEssEDDDDDPFMNTSSLR |
| 2458 | P49959 | GVDFEssEDDDDDPFMNTSSLR |
| 2459 | P49959 | NYSEVIEVDEsDVEEDIFPTTSK |
| 2460 | P49915 | TLNMTTsPEEK |
| 2461 | P49840 | GEPNVSyICSR |
| 2462 | P49840 | GEPNVSyICsR |
| 2463 | P49815 | STsLNERPK |
| 2464 | P49792 | VGEDEDGsDEEVVHNEDIHFEPIVSLPEVEVK |
| 2465 | P49792 | LPPTFFCGVCsDTDEDNGNGEDFQSELQK |
| 2466 | P49792 | LPPTFFCGVCSDtDEDNGNGEDFQSELQK |
| 2467 | P49792 | KIESFGsPK |
| 2468 | P49792 | YIASVQGStPsPR |
| 2469 | P49792 | YIASVQGStPsPR |
| 2470 | P49792 | SALSPSKsPAK |
| 2471 | P49792 | NADSEIKHStPsPTR |
| 2472 | P49792 | NADSEIKHStPsPTR |
| 2473 | P49790 | IPSIVSsPLNsPLDR |
| 2474 | P49790 | IPSIVSsPLNsPLDR |
| 2475 | P49768 | AAVQELSSsILAGEDPEER |
| 2476 | P49756 | LGASNsPGQPNSVK |
| 2477 | P49736 | GNDPLTSsPGR |
| 2478 | P49736 | AESSESFTMASsPAQR |
| 2479 | P49736 | GLLYDsDEEDEERPAR |
| 2480 | P49736 | AEssESFTMASSPAQR |
| 2481 | P49736 | AESsESFtMASSPAQR |
| 2482 | P49736 | RTDALTssPGR |
| 2483 | P49736 | AIPELDAYEAEGLALDDEDVEELTAsQR |
| 2484 | P49736 | RTDALTSsPGR |
| 2485 | P49736 | GNDPLTsSPGR |
| 2486 | P49711 | KEDssDsENAEPDLDDNEDEEEPAVEIEPEPEPQPVTPAPPPAK |
| 2487 | P49711 | KEDssDsENAEPDLDDNEDEEEPAVEIEPEPEPQPVTPAPPPAK |
| 2488 | P49711 | KEDssDsENAEPDLDDNEDEEEPAVEIEPEPEPQPVTPAPPPAK |
| 2489 | P49711 | sKKEDSsDsENAEPDLDDNEDEEEPAVEIEPEPEPQPVTPAPPPAK |
| 2490 | P49450 | RRsPsPTPTPGPSR |
| 2491 | P49450 | RRsPsPTPTPGPSR |
| 2492 | P49354 | HSTENDsPTNVQQ |

| | | |
|---|---|---|
| 2493 | P49327 | ADEASELACPtPK |
| 2494 | P49023 | TSSVSNPQDSVGsPCSR |
| 2495 | P49023 | IsASSATR |
| 2496 | P49023 | QKsAEPsPTVMSTSLGSNLSELDR |
| 2497 | P49023 | QKsAEPsPTVMSTSLGSNLSELDR |
| 2498 | P48681 | sLGEQDQMTLRPPEK |
| 2499 | P48681 | TALETESQDSAEPsGsEEESDPVSLER |
| 2500 | P48681 | TALETESQDSAEPsGsEEESDPVSLER |
| 2501 | P48634 | TAsETRsEGSEYEEIPK |
| 2502 | P48634 | TAsETRsEGSEYEEIPK |
| 2503 | P48634 | ERSDsGGSSSEPFDR |
| 2504 | P47755 | ADLEEQLsDEEKVR |
| 2505 | P47712 | CSVsLSNVEAR |
| 2506 | P47712 | HIVSNDSSDsDDESHEPK |
| 2507 | P47712 | HIVSNDsSDsDDESHEPK |
| 2508 | P46940 | SKsVKEDSNLTLQEK |
| 2509 | P46937 | GDsETDLEALFNAVMNPK |
| 2510 | P46379 | AsPEPQRENAsPAPGTTAEEAMSR |
| 2511 | P46379 | AsPEPQRENAsPAPGTTAEEAMSR |
| 2512 | P46100 | RPTETNPVTSNsDEECNETVK |
| 2513 | P46100 | LTPVsLSNsPIK |
| 2514 | P46100 | LTPVsLSNsPIK |
| 2515 | P46100 | HKLTVsDGEsGEEK |
| 2516 | P46100 | HKLTVsDGEsGEEK |
| 2517 | P46087 | EAAAGIQWsEEEtEDEEEEK |
| 2518 | P46087 | EAAAGIQWsEEEtEDEEEEK |
| 2519 | P46087 | GPQPPTVsPIR |
| 2520 | P46087 | GTDTQTPAVLsPSK |
| 2521 | P46087 | EAAAGIQWsEEEtEDEEEEKEVtPESGPPK |
| 2522 | P46060 | ILDPNTGEPAPVLSSPPPADVSTFLAFPsPEK |
| 2523 | P46013 | SGGSGHAVAEPAsPEQELDQNK |
| 2524 | P46013 | TPVQYSQQQNsPQK |
| 2525 | P46013 | IACKsPQPDPVDTPASTK |
| 2526 | P46013 | IACKsPPPESVDTPTSTK |
| 2527 | P46013 | AQSLVISPPAPsPR |
| 2528 | P45880 | LTFDTTFsPNTGK |
| 2529 | P43243 | RDsFDDR |
| 2530 | P43243 | SYsPDGKESPSDK |
| 2531 | P43243 | SYsPDGKEsPSDK |
| 2532 | P42858 | LLSPQMsGEEEDSDLAAK |
| 2533 | P42858 | SGSIVELIAGGGSSCsPVLSR |

| | | |
|---|---|---|
| 2534 | P42696 | KRsVQEGENPDDGVR |
| 2535 | P42684 | DKsPSSLLEDAK |
| 2536 | P42684 | TVsTSSQPEENVDR |
| 2537 | P42684 | TVsTSsQPEENVDR |
| 2538 | P42166 | GPPDFSSDEEREPtPVLGSGAAAAGR |
| 2539 | P42166 | GPPDFSsDEEREPTPVLGSGAAAAGR |
| 2540 | P42166 | GPPDFsSDEEREPTPVLGsGAAAAGR |
| 2541 | P42166 | EQGTESRSStPLPTISSSAENTR |
| 2542 | P41252 | APLKPYPVsPSDK |
| 2543 | P41227 | GNsPPSSGEACR |
| 2544 | P41227 | DLSEVSETTESTDVKDSSEAsDSAS |
| 2545 | P40855 | NATDLQNSSMsEEELTK |
| 2546 | P40818 | SYsSPDITQAIQEEEK |
| 2547 | P40763 | FICVtPTTCSNTIDLPMsPR |
| 2548 | P40763 | FICVtPTTCSNTIDLPMsPR |
| 2549 | P40425 | TAVSVTQGGHsR |
| 2550 | P40222 | RPEGPGAQAPSsPR |
| 2551 | P40123 | SHTPSPTsPK |
| 2552 | P40123 | SHTPsPTSPK |
| 2553 | P40123 | SHTPSPtSPK |
| 2554 | P38432 | AFQLEEGEEtEPDCK |
| 2555 | P38398 | SDELLGsDDSHDGESEsNAK |
| 2556 | P38398 | SDELLGsDDsHDGESESNAK |
| 2557 | P38159 | DVYLsPR |
| 2558 | P38159 | VEQATKPsFESGR |
| 2559 | P38159 | DYGHsSSRDDYPSR |
| 2560 | P38159 | DsYGGPPR |
| 2561 | P37802 | NFsDNQLQEGK |
| 2562 | P37275 | NNDQPQSANANEPQDSTVNLQsPLK |
| 2563 | P36915 | EEQtDTsDGESVTHHIR |
| 2564 | P36915 | EEQTDTsDGESVTHHIR |
| 2565 | P36915 | DVAGATWGNGsGEEEEEEDGPAVLVEQQTDSAMEPTGPTQER |
| 2566 | P36915 | EEQtDtsDGESVTHHIR |
| 2567 | P36507 | LCDFGVSGQLIDsMANsFVGTR |
| 2568 | P36507 | LCDFGVSGQLIDsMANsFVGTR |
| 2569 | P35680 | GRLsGDEGsEDGDDYDTPPILK |
| 2570 | P35680 | GRLsGDEGsEDGDDYDTPPILK |
| 2571 | P35659 | EEsEEEEDEDDEEEEEEEK |
| 2572 | P35658 | ITPPAAKPGsPQAK |
| 2573 | P35658 | SPGsTPTTPTSSQAPQK |
| 2574 | P35658 | SPGSTPTtPTSSQAPQK |

| | | |
|---|---|---|
| 2575 | P35613 | KPEDVLDDDDAGsAPLK |
| 2576 | P35611 | SPGsPVGEGTGSPPK |
| 2577 | P35606 | STAQQELDGKPAsPTPVIVASHTANK |
| 2578 | P35580 | QLHLEGASLELsDDDTESK |
| 2579 | P35579 | GAGDGsDEEVDGK |
| 2580 | P35269 | GNsRPGTPSAEGGSTSSTLR |
| 2581 | P35269 | GNsRPGtPSAEGGSTSSTLR |
| 2582 | P35269 | STPQPPsGKTtPNSGDVQVTEDAVR |
| 2583 | P35269 | STPQPPsGKtTPNSGDVQVTEDAVR |
| 2584 | P35251 | ESVsPEDSEK |
| 2585 | P35251 | IIYDsDsESEETLQVK |
| 2586 | P35251 | IIYDsDsESEETLQVK |
| 2587 | P35221 | TPEELDDsDFETEDFDVR |
| 2588 | P34947 | DVLDIEQFStVK |
| 2589 | P34947 | DVLDIEQFsTVK |
| 2590 | P33991 | GLQVDLQsDGAAAEDIVASEQSLGQK |
| 2591 | P32004 | EAAGGNDSsGATsPINPAVALE |
| 2592 | P32004 | EAAGGNDsSGATSPINPAVALE |
| 2593 | P31949 | ISsPTETER |
| 2594 | P31943 | HTGPNsPDTANDGFVR |
| 2595 | P31350 | VPLAPITDPQQLQLsPLK |
| 2596 | P31321 | SNSQSDsHDEEVsPTPPNPVVK |
| 2597 | P31321 | SNSQSDSHDEEVsPTPPNPVVK |
| 2598 | P30989 | KADsVSSNHTLSSNATR |
| 2599 | P30622 | TASESISNLSEAGsIK |
| 2600 | P30622 | TASESISNLsEAGsIK |
| 2601 | P30622 | KVQAEDEANGLQTTPAsR |
| 2602 | P30622 | TASEsISNLSEAGsIK |
| 2603 | P30566 | AAGGDHGsPDSYR |
| 2604 | P30419 | GGLsPANDTGAK |
| 2605 | P30414 | ILIPsDIESSK |
| 2606 | P30050 | IGPLGLsPK |
| 2607 | P29966 | EELQANGsAPAADKEEPAAAGSGAASPSAAEKGEPAAAAAPEAGAsPVEK |
| 2608 | P29966 | EAPAEGEAAEPGsPTAAEGEAASAASSTSSPK |
| 2609 | P29966 | LSGFsFK |
| 2610 | P29966 | AEDGATPSPSNEtPK |
| 2611 | P29966 | AEDGATPsPSNETPK |
| 2612 | P29692 | KPATPAEDDEDDDIDLFGsDNEEEDK |
| 2613 | P29692 | KPAtPAEDDEDDDIDLFGsDNEEEDK |
| 2614 | P29692 | ATAPQTQHVsPMR |
| 2615 | P29590 | AVsPPHLDGPPsPR |

| | | |
|---|---|---|
| 2616 | P29590 | AVSPPHLDGPPsPRsPVIGSEVFLPNSNHVASGAGEAEER |
| 2617 | P29590 | AVSPPHLDGPPSPRsPVIGSEVFLPNSNHVASGAGEAEER |
| 2618 | P29590 | LARssPEQPRPSTSK |
| 2619 | P29375 | DLDLEPLsDLEEGLEETR |
| 2620 | P29353 | HGsFVNKPTR |
| 2621 | P29317 | LPsTSGSEGVPFR |
| 2622 | P29317 | VsIRLPsTSGsEGVPFR |
| 2623 | P29317 | LPSTSGsEGVPFR |
| 2624 | P28715 | NAPAAVDEGSIsPR |
| 2625 | P28715 | NAPAAVDEGsISPR |
| 2626 | P28715 | ENDLYVLPPLQEEEKHssEEEDEKEWQER |
| 2627 | P28715 | ENDLYVLPPLQEEEKHssEEEDEKEWQER |
| 2628 | P28715 | FDSSLLssDDETK |
| 2629 | P28715 | FDSSLLSsDDETK |
| 2630 | P28715 | ELTPAsPTCTNSVSK |
| 2631 | P28482 | VADPDHDHTGFLTEyVATR |
| 2632 | P28290 | sQsLPTTLLSPVR |
| 2633 | P28290 | SQsLPTTLLSPVR |
| 2634 | P28290 | DCRtPLGAsLDEQSSSTLK |
| 2635 | P28290 | DCRtPLGAsLDEQSSSTLK |
| 2636 | P28288 | sGKPPLQNNEK |
| 2637 | P28066 | GVNTFsPEGR |
| 2638 | P27824 | AEEDEILNRsPR |
| 2639 | P27824 | QKSDAEEDGGTVsQEEEDR |
| 2640 | P27824 | QKsDAEEDGGTVSQEEEDR |
| 2641 | P27816 | ELPPsPEKK |
| 2642 | P27816 | KCsLPAEEDSVLEK |
| 2643 | P27816 | DMSPLSEtEMALGKDVtPPPETEVVLIK |
| 2644 | P27816 | EAQTLDSQIQETsI |
| 2645 | P27816 | DMEsPTKLDVTLAK |
| 2646 | P27816 | AsPIKMDLAPSK |
| 2647 | P27816 | AsPSKPASAPASR |
| 2648 | P27816 | DMsPLSETEMALGK |
| 2649 | P27816 | DMsPLsETEMALGK |
| 2650 | P27708 | AsDPGLPAEEPK |
| 2651 | P27361 | IADPEHDHTGFLtEyVATR |
| 2652 | P27361 | IADPEHDHTGFLtEyVATR |
| 2653 | P26368 | EEHGGLIRsPR |
| 2654 | P26358 | EADDDEEVDDNIPEMPsPK |
| 2655 | P26358 | SKsDGEAKPEPSPSPR |
| 2656 | P25788 | ESLKEEDEsDDDNM |

| | | |
|---|---|---|
| 2657 | P25490 | DIDHETVVEEQIIGENsPPDYSEYMTGK |
| 2658 | P25440 | ADTTTPTPTAILAPGsPAsPPGSLEPK |
| 2659 | P25440 | ADTTTPTPTAILAPGsPAsPPGSLEPK |
| 2660 | P25205 | RSEDEsEtEDEEEKSQEDQEQK |
| 2661 | P25205 | RSEDEsEtEDEEEKSQEDQEQK |
| 2662 | P25205 | DGDSYDPYDFSDTEEEMPQVHtPK |
| 2663 | P25205 | DGDSYDPYDFSDtEEEMPQVHTPK |
| 2664 | P25205 | DGDSYDPYDFsDTEEEMPQVHTPK |
| 2665 | P24928 | YSPTsPTYSPTSPK |
| 2666 | P24928 | YSPTsPTYSPTTPK |
| 2667 | P24928 | YSPTSPTYSPTsPK |
| 2668 | P24928 | YSPtSPTYSPTsPK |
| 2669 | P24534 | DDDDIDLFGsDDEEESEEAK |
| 2670 | P23588 | SQSSDTEQQsPTSGGGK |
| 2671 | P23588 | YAALsVDGEDENEGEDYAE |
| 2672 | P23588 | ERSRTGsESSQTGTSTTSSR |
| 2673 | P23588 | ESEKsLENETLNK |
| 2674 | P23588 | REsEKsLENETLNK |
| 2675 | P23588 | ERHPsWR |
| 2676 | P23588 | ERHPsWRsEETQER |
| 2677 | P23588 | EEDCHsPTSKPPKPDQPLK |
| 2678 | P23588 | SQSsDTEQQSPTSGGGK |
| 2679 | P23588 | SQssDTEQQSPTSGGGK |
| 2680 | P23588 | SRTGSESsQTGTSTTSSR |
| 2681 | P23528 | AsGVAVSDGVIK |
| 2682 | P23497 | RVIGQDHDFsEssEEEAPAEASSGALR |
| 2683 | P23497 | RVIGQDHDFsEssEEEAPAEASSGALR |
| 2684 | P23497 | RVIGQDHDFsEssEEEAPAEASSGALR |
| 2685 | P23396 | DEILPTtPISEQK |
| 2686 | P23246 | SEEKIsDSEGFK |
| 2687 | P23193 | EPAITSQNsPEAR |
| 2688 | P22626 | GGNFGFGDsR |
| 2689 | P22607 | IARLssGEGPTLANVSELELPADPK |
| 2690 | P22607 | IARLssGEGPTLANVSELELPADPK |
| 2691 | P22392 | NIIHGsDSVK |
| 2692 | P22314 | IHVSDQELQsANAsVDDSRLEELK |
| 2693 | P22314 | IHVSDQELQsANAsVDDSRLEELK |
| 2694 | P22059 | MLAEsDEsGDEESVSQTDK |
| 2695 | P22059 | MLAEsDEsGDEESVSQTDK |
| 2696 | P22059 | DQCCSGKGDMsDEDDENEFFDAPEIITMPENLGHK |
| 2697 | P22059 | TGsNISGASSDISLDEQYK |

| | | |
|---|---|---|
| 2698 | P22059 | RTGSNISGAsSDISLDEQYK |
| 2699 | P22059 | RTGSNIsGASSDISLDEQYK |
| 2700 | P21796 | LTFDSSFsPNTGK |
| 2701 | P21333 | CSGPGLsPGMVR |
| 2702 | P21333 | APsVANVGSHCDLSLK |
| 2703 | P21333 | AFGPGLQGGSAGsPAR |
| 2704 | P21291 | GFGFGQGAGALVHsE |
| 2705 | P21127 | GtsPRPPEGGLGYSQLGDDDLK |
| 2706 | P21127 | GTsPRPPEGGLGYSQLGDDDLK |
| 2707 | P21127 | DLLSDLQDIsDSER |
| 2708 | P20810 | EGITGPPADSSKPIGPDDAIDALSSDFTCGsPTAAGK |
| 2709 | P20700 | LKLsPSPSSR |
| 2710 | P20700 | LKLsPsPSSR |
| 2711 | P20700 | AGGPTTPLsPTR |
| 2712 | P20700 | AGGPTtPLsPTR |
| 2713 | P20042 | DLKIEsDVQEPtEPEDDLDIMLGNK |
| 2714 | P20042 | DLKIEsDVQEPTEPEDDLDIMLGNK |
| 2715 | P20020 | SATSSSPGSPLHSLETsL |
| 2716 | P19532 | AASDPLLSSVsPAVsK |
| 2717 | P19532 | AASDPLLSSVsPAVSK |
| 2718 | P19338 | EDsDEEEDDDsEEDEEDDEDEDEDEDEIEPAAMK |
| 2719 | P19338 | EDsDEEEDDDsEEDEEDDEDEDEDEDEIEPAAMK |
| 2720 | P19338 | AAAAAPASEDEDDEDDEDDEDDDDDEEDDsEEEAMETTPAK |
| 2721 | P19338 | AAAAAPAsEDEDDEDDEDDEDDDDDEEDDSEEEAMETTPAK |
| 2722 | P19338 | KVVVsPTK |
| 2723 | P19338 | EVEEDsEDEEMsEDEEDDSSGEEVVIPQK |
| 2724 | P19338 | EVEEDSEDEEMsEDEEDDssGEEVVIPQK |
| 2725 | P19338 | EVEEDSEDEEMsEDEEDDssGEEVVIPQK |
| 2726 | P19338 | AAAAAPAsEDEDDEDDEDDEDDDDDEEDDsEEEAMETtPAK |
| 2727 | P19338 | EVEEDSEDEEMsEDEEDDssGEEVVIPQK |
| 2728 | P18858 | KQSQIQNQQGEDsGsDPEDTY |
| 2729 | P18858 | KQSQIQNQQGEDsGsDPEDTY |
| 2730 | P18858 | TIQEVLEEQsEDEDR |
| 2731 | P18858 | VLGSEGEEEDEALsPAK |
| 2732 | P18858 | VLGsEGEEEDEALSPAK |
| 2733 | P18858 | AETPTESVsEPEVATK |
| 2734 | P18858 | AEtPTESVSEPEVATK |
| 2735 | P18754 | RRsPPADAIPK |
| 2736 | P18615 | SIsADDDLQESSR |
| 2737 | P18583 | ESDQTLAALLsPK |
| 2738 | P18583 | SVESTsPEPSK |

| | | |
|---|---|---|
| 2739 | P18583 | RRsFsIsPVR |
| 2740 | P18583 | RRsFsIsPVR |
| 2741 | P18583 | EMEHNTVCAAGTsPVGEIGEEK |
| 2742 | P18206 | DPSAsPGDAGEQAIR |
| 2743 | P17987 | HGsYEDAVHSGALND |
| 2744 | P17812 | DTYSDRSGSssPDSEITELK |
| 2745 | P17812 | DTYSDRSGSsSPDSEITELK |
| 2746 | P17812 | DTYSDRsGSSSPDSEITELK |
| 2747 | P17812 | SGsSsPDSEITELK |
| 2748 | P17676 | AYLGYQAVPSGSSGSLSTSSSSSPPGtPSPADAK |
| 2749 | P17676 | AYLGYQAVPSGSSGSLSTSSSSsPPGTPSPADAK |
| 2750 | P17544 | TDSVIIADQtPtPTR |
| 2751 | P17544 | TDSVIIADQtPtPTR |
| 2752 | P17535 | LAsPELER |
| 2753 | P17535 | DEPQTVPDVPSFGEsPPLsPIDMDTQER |
| 2754 | P17535 | DEPQTVPDVPSFGEsPPLsPIDMDTQER |
| 2755 | P17480 | GKLPEsPK |
| 2756 | P17275 | sRDATPPVsPINMEDQER |
| 2757 | P17275 | DAtPPVsPINMEDQER |
| 2758 | P17275 | DAtPPVsPINMEDQER |
| 2759 | P17096 | EEEEGISQEssEEEQ |
| 2760 | P17096 | EEEEGISQEsSEEEQ |
| 2761 | P17096 | EEEEGIsQEssEEEQ |
| 2762 | P17029 | EATGLsPQAAQEK |
| 2763 | P16989 | sEAGEATTTTTTTLPQAPTEAAAAAPQDPAPK |
| 2764 | P16949 | ASGQAFELILsPR |
| 2765 | P16949 | AsGQAFELILSPR |
| 2766 | P16949 | RKsHEAEVLK |
| 2767 | P16949 | ESVPEFPLsPPK |
| 2768 | P16615 | EFDELNPsAQR |
| 2769 | P16455 | GAGATSGsPPAGR |
| 2770 | P16403 | sETAPAAPAAAPPAEK |
| 2771 | P16401 | SETAPAETATPAPVEKsPAK |
| 2772 | P16401 | SETAPAETAtPAPVEK |
| 2773 | P16401 | sETAPAETATPAPVEK |
| 2774 | P16383 | AADssDsDGAEESPAEPGAPR |
| 2775 | P16383 | AADssDsDGAEESPAEPGAPR |
| 2776 | P16383 | AADssDsDGAEESPAEPGAPR |
| 2777 | P16333 | KPsVPDsASPADDSFVDPGER |
| 2778 | P15924 | AEsGPDLR |
| 2779 | P15924 | GLPSPYNMSSAPGsR |

| | | |
|---|---|---|
| 2780 | P15924 | GLPSPYNMSsAPGsR |
| 2781 | P15924 | SMsFQGIR |
| 2782 | P15924 | AsSKGGGGyTCQSGSGWDEFTK |
| 2783 | P15924 | AssKGGGGYTCQSGSGWDEFTK |
| 2784 | P15924 | GGGGYTCQsGSGWDEFTK |
| 2785 | P15531 | NIIHGsDSVESAEK |
| 2786 | P15408 | DEQLsPEEEEK |
| 2787 | P15408 | RsPPAPGLQPMR |
| 2788 | P15408 | QEPLEEDsPSSSSAGLDK |
| 2789 | P15336 | NDSVIVADQtPtPTR |
| 2790 | P15336 | NDSVIVADQtPtPTR |
| 2791 | P14618 | LDIDsPPITAR |
| 2792 | P13861 | GDSEsEEDEDLEVPVPSR |
| 2793 | P13861 | GDsEsEEDEDLEVPVPSR |
| 2794 | P13861 | RVsVCAETYNPDEEEEDTDPR |
| 2795 | P13807 | NSVDTATSSSLSTPSEPLsPTSSLGEER |
| 2796 | P13807 | NSVDTATSSSLSTPSEPLsPTSsLGEER |
| 2797 | P13489 | sLDIQSLDIQCEELSDAR |
| 2798 | P13051 | HAPsPEPAVQGTGVAGVPEESGDAAAIPAK |
| 2799 | P13051 | APAGQEEPGtPPsSPLSAEQLDR |
| 2800 | P13051 | KAPAGQEEPGtPPSsPLSAEQLDR |
| 2801 | P13051 | APAGQEEPGtPPsSPLSAEQLDR |
| 2802 | P12931 | sLEPAENVHGAGGGAFPASQTPSKPASADGHR |
| 2803 | P12270 | TDGFAEAIHsPQVAGVPR |
| 2804 | P12270 | EGVQGPLNVsLSEEGK |
| 2805 | P11717 | ALSSLHGDDQDsEDEVLTIPEVK |
| 2806 | P11532 | GLSPLPSPPEMMPtSPQsPR |
| 2807 | P11474 | AEPAsPDSPKGSSETETEPPVALAPGPAPTR |
| 2808 | P11388 | ELKPQKsVVsDLEADDVK |
| 2809 | P11388 | ELKPQKsVVsDLEADDVK |
| 2810 | P11388 | IKNENTEGsPQEDGVELEGLK |
| 2811 | P11388 | VPDEEENEEsDNEK |
| 2812 | P11388 | GSVPLSSsPPATHFPDETEITNPVPK |
| 2813 | P11388 | GSVPLSssPPATHFPDETEITNPVPK |
| 2814 | P11388 | FTMDLDsDEDFsDFDEKtDDEDFVPSDASPPK |
| 2815 | P11388 | FTMDLDsDEDFsDFDEK |
| 2816 | P11388 | FTMDLDsDEDFsDFDEK |
| 2817 | P11387 | SGDHLHNDsQIEADFR |
| 2818 | P11171 | EEsPQsKAETELK |
| 2819 | P11171 | EEsPQsKAETELK |
| 2820 | P10644 | EDEIsPPPPNPVVK |

| | | |
|---|---|---|
| 2821 | P10644 | tDSREDEIsPPPPNPVVK |
| 2822 | P10636 | AKTDHGAEIVYKsPVVsGDTsPR |
| 2823 | P10636 | AKTDHGAEIVYKsPVVsGDTsPR |
| 2824 | P10588 | AAEDDSASPPGAAsDAEPGDEERPGLQVDCVVCGDK |
| 2825 | P10588 | AAEDDSAsPPGAAsDAEPGDEERPGLQVDCVVCGDK |
| 2826 | P10451 | ISHELDsASSEVN |
| 2827 | P10451 | ISHELDsAsSEVN |
| 2828 | P10412 | SETAPAAPAAPAPAEKtPVK |
| 2829 | P10412 | sETAPAAPAAPAPAEK |
| 2830 | P10398 | GGSDGtPRGsPSPASVSSGR |
| 2831 | P10398 | GGSDGtPRGSPsPASVSSGR |
| 2832 | P10398 | GGSDGtPRGSPsPASVSSGR |
| 2833 | P0DJ93 | ELVGDTGSQEGDHEPsGsETEEDTSSSPHR |
| 2834 | P0DJ93 | ELVGDTGSQEGDHEPsGsETEEDTSSSPHR |
| 2835 | P0C1Z6 | RTPAPPEPGsPAPGEGPSGR |
| 2836 | P0C1Z6 | LLPYPTLASPAsD |
| 2837 | P0C1Z6 | LLPYPTLAsPAsD |
| 2838 | P09884 | sIGASPNPFSVHTATAVPSGK |
| 2839 | P09874 | GGsDDSSKDPIDVNYEK |
| 2840 | P09651 | SEsPKEPEQLR |
| 2841 | P09651 | sKSESPKEPEQLR |
| 2842 | P08910 | LQCsDTEQVEADLE |
| 2843 | P08833 | AQETsGEEISK |
| 2844 | P08729 | SIHFSsPVFTSR |
| 2845 | P08670 | ETNLDsLPLVDTHSK |
| 2846 | P08651 | SGSMEEDVDTSPGGDYYTSPSsPTSSSR |
| 2847 | P08621 | GGGGDMAEPsEAGDAPPDDGPPGELGPDGPDGPEEK |
| 2848 | P08621 | GGGGGQDNGLEGLGNDsR |
| 2849 | P08621 | YDERPGPsPLPHR |
| 2850 | P08559 | YGMGTsVER |
| 2851 | P08559 | YHGHSMSDPGVsYR |
| 2852 | P08559 | YHGHsMSDPGVSYR |
| 2853 | P08238 | EIsDDEAEEEK |
| 2854 | P08238 | IEDVGsDEEDDSGK |
| 2855 | P08238 | IEDVGsDEEDDsGKDK |
| 2856 | P08047 | SDQDHsMDEMTAVVK |
| 2857 | P07910 | DDEKEAEEGEDDRDsANGEDDS |
| 2858 | P07900 | DKEVsDDEAEEK |
| 2859 | P07900 | EEKESEDKPEIEDVGsDEEEEK |
| 2860 | P07814 | EYIPGQPPLSQsSDSSPTR |
| 2861 | P07814 | EYIPGQPPLSQSSDSsPTR |

| | | |
|---|---|---|
| 2862 | P06748 | CGSGPVHISGQHLVAVEEDAEsEDEEEEDVK |
| 2863 | P06748 | DELHIVEAEAMNYEGsPIK |
| 2864 | P06733 | sGKYDLDFK |
| 2865 | P06493 | IGEGtYGVVYK |
| 2866 | P06493 | IGEGTyGVVYK |
| 2867 | P06454 | sDAAVDTSSEITTK |
| 2868 | P06454 | SDAAVDTsSEITTK |
| 2869 | P06454 | SDAAVDtsSEITTK |
| 2870 | P06400 | ISEGLPTPtKMtPR |
| 2871 | P06400 | ISEGLPtPTKMtPR |
| 2872 | P06241 | DGsLNQSSGYR |
| 2873 | P05787 | AsLEAAIADAEQR |
| 2874 | P05787 | AFsSRsYTSGPGSR |
| 2875 | P05787 | ISSSsFSR |
| 2876 | P05783 | sTFSTNYR |
| 2877 | P05455 | FAsDDEHDEHDENGATGPVK |
| 2878 | P05412 | NSDLLTsPDVGLLK |
| 2879 | P05388 | EESEEsDEDMGFGLFD |
| 2880 | P05388 | EEsEEsDEDMGFGLFD |
| 2881 | P05387 | DEKKEEsEEsDDDMGFGLFD |
| 2882 | P05387 | DEKKEEsEEsDDDMGFGLFD |
| 2883 | P05187 | HVPDsGATATAYLCGVK |
| 2884 | P05114 | TEESPASDEAGEKEAKsD |
| 2885 | P05114 | EDLPAENGETKTEESPAsDEAGEK |
| 2886 | P05114 | EDLPAENGETKTEEsPASDEAGEK |
| 2887 | P05114 | KVsSAEGAAKEEPK |
| 2888 | P05023 | DKYEPAAVsEQGDKK |
| 2889 | P04920 | ALTQPsPVSTPSSVQFFLQEDDSADR |
| 2890 | P04792 | GPsWDPFR |
| 2891 | P04792 | QLsSGVSEIR |
| 2892 | P04792 | AQLGGPEAAKsDETAAK |
| 2893 | P04183 | SCINLPTVLPGsPSK |
| 2894 | P04075 | LQsIGTENTEENR |
| 2895 | P04049 | SAsEPSLHR |
| 2896 | P04049 | RAsDDGKLTDPSK |
| 2897 | P04049 | SHSESASPSALSSSPNNLsPTGWSQPK |
| 2898 | P04049 | SHSESASPSALSSsPNNLsPTGWSQPK |
| 2899 | P02794 | HTLGDsDNES |
| 2900 | P02765 | CDSSPDsAEDVR |
| 2901 | P02545 | SGAQASSTPLsPTR |
| 2902 | P02545 | LRLsPsPTSQR |

| | | |
|---|---|---|
| 2903 | P02545 | sGAQASSTPLSPTR |
| 2904 | P02545 | SGAQASStPLsPTR |
| 2905 | P02545 | LRLsPSPTSQR |
| 2906 | P02545 | NKsNEDQSMGNWQIK |
| 2907 | P02545 | LRLsPsPTsQR |
| 2908 | P02545 | sVGGSGGGSFGDNLVTR |
| 2909 | P00533 | ELVEPLtPSGEAPNQALLR |
| 2910 | O96019 | EGsPANWK |
| 2911 | O96013 | RDsPPPPAR |
| 2912 | O96013 | DKRPLsGPDVGTPQPAGLASGAK |
| 2913 | O95999 | SNsDESNFSEK |
| 2914 | O95997 | LFQLGPPsPVK |
| 2915 | O95835 | sNSFNNPLGNR |
| 2916 | O95817 | SSVQGASsREGsPAR |
| 2917 | O95817 | SSVQGASsREGsPAR |
| 2918 | O95817 | SSTPLHsPSPIR |
| 2919 | O95817 | SQsPAASDCSSSSSSASLPSSGR |
| 2920 | O95817 | VPPAPVPCPPPsPGPSAVPSsPK |
| 2921 | O95817 | VPPAPVPCPPPsPGPSAVPsSPK |
| 2922 | O95817 | SSVQGAsSREGsPAR |
| 2923 | O95785 | NPEDKSPQLSLsPRPAsPK |
| 2924 | O95785 | NPEDKSPQLSLsPRPAsPK |
| 2925 | O95785 | TPLALAGsPTPK |
| 2926 | O95772 | AALIPGGLSDGQFYsPPEsEAGsEEAEEK |
| 2927 | O95772 | AALIPGGLSDGQFYsPPEsEAGsEEAEEK |
| 2928 | O95772 | AALIPGGLSDGQFYsPPEsEAGsEEAEEK |
| 2929 | O95747 | TEDGGWEWsDDEFDEESEEGK |
| 2930 | O95714 | LAELPAAAQPSAEDsDtEDDsEAEQTER |
| 2931 | O95714 | LAELPAAAQPSAEDsDtEDDSEAEQTER |
| 2932 | O95714 | LAELPAAAQPSAEDsDtEDDSEAEQTER |
| 2933 | O95696 | VHGEPTsDLsDID |
| 2934 | O95696 | VHGEPTsDLsDID |
| 2935 | O95685 | ARsLPSsPER |
| 2936 | O95685 | ARsLPSsPER |
| 2937 | O95684 | EKGPTTGEGALDLSDVHsPPKsPEGK |
| 2938 | O95684 | EKGPTTGEGALDLSDVHsPPKsPEGK |
| 2939 | O95684 | GPTTGEGALDLsDVHSPPKsPEGK |
| 2940 | O95671 | HDSIPAADTFEDLsDVEGGGSEPTQR |
| 2941 | O95625 | VEVAHIsGGE |
| 2942 | O95544 | SLsASPALGSTK |
| 2943 | O95466 | RDsELGPGVK |

| | | |
|---|---|---|
| 2944 | O95466 | EAAAQEAGADTPGKGEPPAPKsPPK |
| 2945 | O95453 | ELsPAGSISK |
| 2946 | O95453 | NLsPSQEEAGLEDGVSGEIsDTELEQTDSCAEPLSEGR |
| 2947 | O95453 | NLSPSQEEAGLEDGVSGEIsDTELEQTDSCAEPLSEGR |
| 2948 | O95400 | GKHSLDsDEEEDDDDGGSSK |
| 2949 | O95400 | GPGQPSsPQR |
| 2950 | O95394 | STIGVMVTAsHNPEEDNGVK |
| 2951 | O95391 | LVEQANsPK |
| 2952 | O95365 | HFKDEDEDEDVAsPDGLGR |
| 2953 | O95361 | ATAQPPAPLSPDSGsPSPDSGSASPVEEEDVGSSEK |
| 2954 | O95361 | ATAQPPAPLsPDSGSPSPDSGSASPVEEEDVGSSEK |
| 2955 | O95361 | ATAQPPAPLSPDSGSPSPDSGSAsPVEEEDVGSSEK |
| 2956 | O95361 | ATAQPPAPLSPDSGSPSPDsGSAsPVEEEDVGSSEK |
| 2957 | O95359 | VQNsPPVGR |
| 2958 | O95359 | LDNTPAsPPRsPAEPNDIPIAK |
| 2959 | O95359 | LDNTPAsPPRsPAEPNDIPIAK |
| 2960 | O95297 | SESVVyADIR |
| 2961 | O95297 | DYTGCSTSESLsPVK |
| 2962 | O95297 | DYTGCSTsESLSPVK |
| 2963 | O95292 | sLSSsLDDTEVKK |
| 2964 | O95292 | sLSSsLDDTEVKK |
| 2965 | O95292 | SLsSSLDDTEVKK |
| 2966 | O95251 | SQQQPTPVtPK |
| 2967 | O95251 | LSQsSQDSSPVR |
| 2968 | O95251 | QTRSSGsETEQVVDFSDR |
| 2969 | O95251 | QTRssGsETEQVVDFSDR |
| 2970 | O95243 | SLsSGSNFCSEQK |
| 2971 | O95239 | NQsLVEENEK |
| 2972 | O95239 | RRtFsLTEVR |
| 2973 | O95239 | ALASNTSFFSGCsPIEEEAH |
| 2974 | O95232 | ESDTKNEVNGTSEDIKSEGDTQsN |
| 2975 | O95218 | EVEDKEsEGEEEDEDEDLSK |
| 2976 | O95218 | EEsDGEYDEFGR |
| 2977 | O95218 | LDEDEDEDDADLSKYNLDAsEEEDSNK |
| 2978 | O95155 | SQSSEGVSSLSSsPSNSLETQSQSLSR |
| 2979 | O95071 | RIsQSQPVR |
| 2980 | O95049 | sPGGGSEANGLALVSGFK |
| 2981 | O95049 | VHDAEssDEDGYDWGPATDL |
| 2982 | O95049 | VHDAEssDEDGYDWGPATDL |
| 2983 | O94979 | DSDQVAQsDGEESPAAEEQLLGEHIK |
| 2984 | O94979 | DSDQVAQSDGEEsPAAEEQLLGEHIK |

| | | |
|---|---|---|
| 2985 | O94979 | EQTLsPTITSGLHNIAR |
| 2986 | O94906 | LSQVSDSVSGQtVVDPK |
| 2987 | O94888 | SESLIDASEDsQLEAAIR |
| 2988 | O94880 | NSADDEELTNDsLTLSQSK |
| 2989 | O94880 | NSADDEELtNDSLTLSQSK |
| 2990 | O94826 | AsPAPGSGHPEGPGAHLDMNSLDR |
| 2991 | O94808 | LDsSACLHAVGDK |
| 2992 | O94804 | NLKtLQK |
| 2993 | O94804 | QVAEQGGDLsPAANR |
| 2994 | O94804 | EMGsLSIKDPK |
| 2995 | O94804 | ASQsRPNsSALETLGGEK |
| 2996 | O94804 | ASQSRPNsSALETLGGEK |
| 2997 | O94763 | KNSTGSGHsAQELPTIR |
| 2998 | O94763 | KNsTGSGHSAQELPTIR |
| 2999 | O75976 | SLLSHEFQDEtDtEEETLYSSK |
| 3000 | O75976 | SLLSHEFQDEtDTEEETLYSSK |
| 3001 | O75971 | SHVTEEEEEEEEEEsDS |
| 3002 | O75970 | TAPTALGITLsSsPTSTPELR |
| 3003 | O75962 | RLssGKADGHVK |
| 3004 | O75962 | RLssGKADGHVK |
| 3005 | O75962 | AGAAsPLNsPLSSAVPSLGK |
| 3006 | O75962 | AGAAsPLNsPLSSAVPSLGK |
| 3007 | O75925 | TCPsLsPTSPLNNK |
| 3008 | O75925 | TCPsLsPTSPLNNK |
| 3009 | O75822 | AAAAAAAGDsDSWDADAFSVEDPVR |
| 3010 | O75822 | AAAAAAAGDSDsWDADAFSVEDPVR |
| 3011 | O75822 | AAAAAAAGDsDSWDADAFsVEDPVR |
| 3012 | O75821 | GIPLATGDtsPEPELLPGAPLPPPK |
| 3013 | O75821 | GIPLATGDTsPEPELLPGAPLPPPK |
| 3014 | O75764 | SSASSsPKRPsVER |
| 3015 | O75764 | SSASSsPKRPSVER |
| 3016 | O75694 | AAPQsPSVPK |
| 3017 | O75683 | ELsPAALEK |
| 3018 | O75674 | EATNTTSEPSAPSQDLLDLSPsPR |
| 3019 | O75643 | EEAsDDDMEGDEAVVR |
| 3020 | O75607 | HQIVTMSNDVSEEEsEEEEEDsDEEEVELCPILPAK |
| 3021 | O75607 | HQIVTMSNDVSEEEsEEEEEDsDEEEVELCPILPAK |
| 3022 | O75607 | HQIVTMSNDVsEEESEEEEEDsDEEEVELCPILPAK |
| 3023 | O75569 | AEAPPLEREDsGTFSLGK |
| 3024 | O75533 | RWDQTADQTPGAtPK |
| 3025 | O75533 | RWDQTADQtPGAtPK |

| | | |
|---|---|---|
| 3026 | O75533 | GGDSIGEtPTPGASK |
| 3027 | O75533 | GGDSIGEtPtPGASK |
| 3028 | O75533 | DTPGHGSGWAEtPR |
| 3029 | O75533 | KLSSWDQAEtPGHTPsLR |
| 3030 | O75533 | KLSSWDQAEtPGHtPSLR |
| 3031 | O75530 | LSSDENSNPDLsGDENDDAVSIESGTNTERPDTPtNTPNAPGR |
| 3032 | O75530 | LSSDENSNPDLsGDENDDAVSIESGTNTERPDtPTNTPNAPGR |
| 3033 | O75494 | sFDYNYR |
| 3034 | O75494 | sRsFDYNYR |
| 3035 | O75475 | TGVTSTsDsEEEGDDQEGEK |
| 3036 | O75475 | TGVTSTsDsEEEGDDQEGEK |
| 3037 | O75475 | EDtDHEEKAsNEDVTK |
| 3038 | O75475 | EDTDHEEKAsNEDVTK |
| 3039 | O75475 | QSNASsDVEVEEK |
| 3040 | O75448 | LLSSNEDDANILSsPTDR |
| 3041 | O75448 | LLsSNEDDANILSSPTDR |
| 3042 | O75446 | KGsDDDGGDSPVQDIDTPEVDLYQLQVNTLR |
| 3043 | O75446 | KGsDDDGGDsPVQDIDTPEVDLYQLQVNTLR |
| 3044 | O75420 | SLsVPDSGR |
| 3045 | O75400 | HKsDsPEsDAER |
| 3046 | O75400 | HKSDsPEsDAER |
| 3047 | O75400 | HKSDsPEsDAER |
| 3048 | O75396 | NLGsINTELQDVQR |
| 3049 | O75379 | NLLEDDsDEEEDFFLR |
| 3050 | O75376 | EPAPLLSAQYETLSDsDD |
| 3051 | O75376 | EPAPLLSAQYETLsDsDD |
| 3052 | O75376 | HEAPSsPISGQPCGDDQNASPSK |
| 3053 | O75369 | APsVATVGSICDLNLK |
| 3054 | O75351 | EGQPSPADEKGNDsDGEGESDDPEK |
| 3055 | O75153 | SEDPPGQEAGsEEEGSSASGLAK |
| 3056 | O75152 | KFsAGGDSDPPLK |
| 3057 | O75152 | RLsSASTGKPPLSVEDDFEK |
| 3058 | O75151 | DSDYVYPSLEsDEDNPIFK |
| 3059 | O75151 | KGsDDAPYSPTAR |
| 3060 | O75151 | KGsDDAPYsPTAR |
| 3061 | O75122 | SRsDIDVNAAAGAK |
| 3062 | O75030 | LEDILMDDTLSPVGVTDPLLSSVsPGASK |
| 3063 | O75030 | LEDILMDDTLsPVGVTDPLLSSVsPGASK |
| 3064 | O60841 | IEPIPGEsPK |
| 3065 | O60841 | NKPGPNIEsGNEDDDASFK |
| 3066 | O60841 | KWDGsEEDEDNSK |

| | | |
|---|---|---|
| 3067 | O60841 | KQSFDDNDsEELEDK |
| 3068 | O60841 | KQsFDDNDSEELEDKDSK |
| 3069 | O60841 | VEMYSGsDDDDDFNKLPK |
| 3070 | O60841 | VEMYsGsDDDDDFNK |
| 3071 | O60841 | VEMySGsDDDDDFNKLPK |
| 3072 | O60832 | AKEVELVsE |
| 3073 | O60832 | KRESESEsDEtPPAAPQLIK |
| 3074 | O60832 | EsEsESDETPPAAPQLIK |
| 3075 | O60832 | EsEsESDETPPAAPQLIK |
| 3076 | O60832 | AGLEsGAEPGDGDSDTTK |
| 3077 | O60832 | AGLESGAEPGDGDsDTTK |
| 3078 | O60825 | NsFTPLSSSNTIR |
| 3079 | O60825 | NYsVGSRPLKPLsPLR |
| 3080 | O60825 | NYsVGSRPLKPLsPLR |
| 3081 | O60784 | AGLQsLEASGR |
| 3082 | O60763 | DLGHPVEEEDELEsGDQEDEDDESEDPGK |
| 3083 | O60762 | ASLEVSRsPR |
| 3084 | O60716 | GSLAsLDsLR |
| 3085 | O60716 | GSLAsLDsLR |
| 3086 | O60678 | GAVENEEDLPELsDsGDEAAWEDEDDADLPHGK |
| 3087 | O60678 | GAVENEEDLPELsDsGDEAAWEDEDDADLPHGK |
| 3088 | O60566 | KLsPIIEDSR |
| 3089 | O60524 | DELNEELIQEEssEDEGEYEEVR |
| 3090 | O60524 | DELNEELIQEEssEDEGEYEEVR |
| 3091 | O60524 | NPYLLsEEEDDDVDGDVNVEK |
| 3092 | O60504 | HPssPSALR |
| 3093 | O60504 | HPSsPSALR |
| 3094 | O60504 | LCDDGPQLPTsPR |
| 3095 | O60502 | LENEGsDEDIETDVLYSPQMALK |
| 3096 | O60341 | KLPPPPPQAPPEEENEsEPEEPSGVEGAAFQSR |
| 3097 | O60341 | EMDESLANLsEDEYYsEEER |
| 3098 | O60341 | EMDESLANLsEDEYYsEEER |
| 3099 | O60293 | KPISDNSFssDEEQSTGPIK |
| 3100 | O60293 | KPISDNSFssDEEQSTGPIK |
| 3101 | O60293 | SVVVTLNDSDDsEsDGEAsK |
| 3102 | O60293 | SVVVTLNDsDDsESDGEASK |
| 3103 | O60293 | SVVVTLNDSDDsEsDGEAsK |
| 3104 | O60293 | ATADTPAPASSGLsPK |
| 3105 | O60293 | ATADTPAPASSGLSPKEEGELEDGEIsDDDNNsQIR |
| 3106 | O60293 | TSSSsPANsDVEIDGIGR |
| 3107 | O60293 | TSSSsPANsDVEIDGIGR |

| | | |
|---|---|---|
| 3108 | O60292 | EVsPAPAVAGQSK |
| 3109 | O60292 | TLsDESLCSGR |
| 3110 | O60271 | ERPIsLGIFPLPAGDGLLtPDAQK |
| 3111 | O60271 | ERPIsLGIFPLPAGDGLLtPDAQK |
| 3112 | O60271 | LHQLsGSDQLESTAHSR |
| 3113 | O60271 | LHQLSGsDQLESTAHSR |
| 3114 | O60271 | SASQSsLDKLDQELK |
| 3115 | O60271 | SAsQSSLDKLDQELK |
| 3116 | O60271 | ETDYPAGEDLSEsGQVDK |
| 3117 | O60264 | GGPEGVAAQAVASAASAGPADAEMEEIFDDAsPGK |
| 3118 | O60239 | SECSGAssPECEVER |
| 3119 | O60239 | SECSGAssPECEVER |
| 3120 | O60231 | LLEDsEESSEETVSR |
| 3121 | O60231 | LLEDsEEsSEETVSR |
| 3122 | O60231 | LLEDsEESsEETVSR |
| 3123 | O43933 | TAsQEGCQELTQEQR |
| 3124 | O43896 | LYADsDsGDDSDKR |
| 3125 | O43896 | LYADsDsGDDSDKR |
| 3126 | O43847 | RGsLSNAGDPEIVK |
| 3127 | O43823 | VDSEGDFsENDDAAGDFR |
| 3128 | O43823 | VDsEGDFsENDDAAGDFR |
| 3129 | O43823 | VDsEGDFsENDDAAGDFRsGDEEFKGEDELCDSGR |
| 3130 | O43818 | MNEEIssDsESESLAPR |
| 3131 | O43818 | MNEEISsDsESESLAPR |
| 3132 | O43818 | MNEEISsDsESESLAPR |
| 3133 | O43815 | FLESAAADFsDEDEDDDVDGR |
| 3134 | O43765 | SRTPsASNDDQQE |
| 3135 | O43765 | sRTPsASNDDQQE |
| 3136 | O43719 | VFDDEsDEKEDEEYADEK |
| 3137 | O43719 | VLDEEGsER |
| 3138 | O43719 | EFDEDsDEKEEEEDTYEK |
| 3139 | O43707 | FAIQDIsVEETSAK |
| 3140 | O43683 | DGKFsPIQEK |
| 3141 | O43663 | GLAPNtPGK |
| 3142 | O43598 | YFEADPPGQVAAsPDPTT |
| 3143 | O43583 | LTVENsPK |
| 3144 | O43432 | RsPVPAQIAITVPK |
| 3145 | O43432 | LDFIESDsPCSSEALSK |
| 3146 | O43399 | MDSAGQDINLNsPNK |
| 3147 | O43395 | GDDDEEsDEEAVK |
| 3148 | O43318 | sIQDLTVTGTEPGQVSSR |

| | | |
|---|---|---|
| 3149 | O43314 | sGEQITSSPVSPK |
| 3150 | O43314 | sGEQITSSPVsPK |
| 3151 | O43306 | DAEPPsPTPAGPPR |
| 3152 | O43294 | KRPSLPSSPsPGLPK |
| 3153 | O43294 | KRPsLPSSPsPGLPK |
| 3154 | O43294 | KRPsLPSsPSPGLPK |
| 3155 | O43294 | KRPsLPsSPSPGLPK |
| 3156 | O43290 | VENMDIsDEEEGGAPPPGsPQVLEEDEAELELQK |
| 3157 | O43290 | VENMDIsDEEEGGAPPPGsPQVLEEDEAELELQK |
| 3158 | O43237 | DFQDYMEPEEGCQGsPQR |
| 3159 | O43159 | QGPPCsDsEEEVER |
| 3160 | O43159 | QGPPCsDsEEEVER |
| 3161 | O43159 | ALEAASLSQHPPSLCIsDsEEEEEER |
| 3162 | O43159 | ALEAASLSQHPPSLCIsDsEEEEEER |
| 3163 | O43159 | ALEAASLSQHPPsLCIsDSEEEEEER |
| 3164 | O43149 | LPSSSGLPAADVSPATAEEPLsPSTPTR |
| 3165 | O15541 | AAYGDLssEEEEENEPESLGVVYK |
| 3166 | O15541 | AAYGDLssEEEEENEPESLGVVYK |
| 3167 | O15541 | YGVYEDENYEVGsDDEEIPFK |
| 3168 | O15530 | ANsFVGTAQYVSPELLTEK |
| 3169 | O15417 | LALsPEDKPIRLsPSK |
| 3170 | O15417 | LALsPEDKPIRLsPSK |
| 3171 | O15371 | VYSLPDGTFsSDEDEEEEEEEEEEEEEEET |
| 3172 | O15371 | VYSLPDGTFssDEDEEEEEEEEEEEEEEET |
| 3173 | O15258 | VDPSLMEDsDDGPSLPTK |
| 3174 | O15234 | QsGDGQESTEPVENK |
| 3175 | O15234 | DPsPEADAPVLGSPEKEEAAsEPPAAAPDAAPPPPDRPIEK |
| 3176 | O15231 | GGQGDPAVPAQQPADPSTPERQSsPsGsEQLVR |
| 3177 | O15231 | GGQGDPAVPTQQPADPStPEQQNSPSGSEQFVR |
| 3178 | O15173 | LLKPGEEPSEYtDEEDTK |
| 3179 | O15164 | NEsEDNKFsDDsDDDFVQPR |
| 3180 | O15164 | NESEDNKFsDDsDDDFVQPR |
| 3181 | O15164 | NESEDNKFsDDsDDDFVQPR |
| 3182 | O15155 | SLsIEIGHEVK |
| 3183 | O15119 | DLCPsEGEsDAEAESK |
| 3184 | O15119 | DLCPsEGEsDAEAESK |
| 3185 | O15085 | SLGGESsGGTTPVGSFHTEAAR |
| 3186 | O15085 | NSVLsDPGLDSPRtsPVIMAR |
| 3187 | O15085 | NSVLSDPGLDsPRTsPVIMAR |
| 3188 | O15085 | NSVLSDPGLDsPRtSPVIMAR |
| 3189 | O15085 | SLGGESsGGTtPVGSFHTEAAR |

| | | |
|---|---|---|
| 3190 | O15085 | HQVLLEDPEQEGsAEEEELGVLPCPSTSLDGENR |
| 3191 | O15061 | QRsPAPGsPDEEGGAEAPAAGIR |
| 3192 | O15061 | QRsPAPGsPDEEGGAEAPAAGIR |
| 3193 | O15027 | AQQELVPPQQQAsPPQLPK |
| 3194 | O15027 | ADSGPTQPPLSLsPAPETK |
| 3195 | O15027 | GSVSQPStPsPPKPTGIFQTSANSSFEPVK |
| 3196 | O15027 | GSVSQPStPsPPKPTGIFQTSANSSFEPVK |
| 3197 | O15014 | LVEPHsPSPSSK |
| 3198 | O15014 | LVEPHsPsPSSK |
| 3199 | O15014 | FCDsPTsDLEMR |
| 3200 | O15013 | YDTNNNEEEEGEQFDFDsGDEIPEADR |
| 3201 | O14974 | LAsTSDIEEK |
| 3202 | O14950 | AtSNVFAMFDQSQIQEFK |
| 3203 | O14950 | ATsNVFAMFDQSQIQEFK |
| 3204 | O14908 | SAGGRPGsGPQLGTGR |
| 3205 | O14828 | KLsPTEPK |
| 3206 | O14777 | NsQLGIFSSSEK |
| 3207 | O14745 | EALAEAALEsPRPALVR |
| 3208 | O14737 | KVMDsDEDDDY |
| 3209 | O14681 | FPsPHPsPAK |
| 3210 | O14681 | FPsPHPsPAK |
| 3211 | O14639 | TLsPTPSAEGYQDVR |
| 3212 | O14639 | TLsPTPsAEGYQDVR |
| 3213 | O14639 | STsQGsINSPVYSR |
| 3214 | O14639 | STsQGSINSPVYSR |
| 3215 | O14617 | NTETSKsPEKDVPMVEK |
| 3216 | O14613 | LHLEtPQPsPQEGGSVDIWR |
| 3217 | O14613 | LHLEtPQPsPQEGGSVDIWR |
| 3218 | O14545 | LDSQPQETsPELPR |
| 3219 | O14497 | GPsPSPVGSPASVAQSR |
| 3220 | O00629 | NVPHEDICEDsDIDGDYR |
| 3221 | O00571 | GKsSFFSDR |
| 3222 | O00567 | EELMSsDLEETAGSTSIPK |
| 3223 | O00567 | EELMssDLEETAGSTSIPK |
| 3224 | O00566 | KsPVFSDEDsDLDFDISK |
| 3225 | O00566 | KSPVFsDEDsDLDFDISK |
| 3226 | O00566 | KSPVFsDEDsDLDFDISK |
| 3227 | O00505 | NVPQEESLEDsDVDADFK |
| 3228 | O00505 | NVPQEEsLEDsDVDADFK |
| 3229 | O00499 | GNKSPsPPDGsPAATPEIR |
| 3230 | O00499 | GNKsPSPPDGSPAATPEIR |

| | | |
|---|---|---|
| 3231 | O00499 | GNKSPsPPDGSPAATPEIR |
| 3232 | O00443 | SQsLNIR |
| 3233 | O00410 | RQDEDYDEQVEEsLQDEDDNDVYILTK |
| 3234 | O00401 | AIHssDEDEDEDDEEDFEDDDEWED |
| 3235 | O00401 | AIHssDEDEDEDDEEDFEDDDEWED |
| 3236 | O00308 | TTPATGEQsPGAR |
| 3237 | O00303 | TCFsPNR |
| 3238 | O00273 | AsPPGDLQNPK |
| 3239 | O00264 | GDQPAASGDsDDDEPPPLPR |
| 3240 | O00264 | EGEEPTVYsDEEEPK |
| 3241 | O00264 | GDQPAAsGDSDDDEPPPLPR |
| 3242 | O00220 | FIYLEDGTGSAVsLE |
| 3243 | O00203 | EGDELEDNGKNFYEsDDDQKEK |
| 3244 | O00193 | RsAsPDDDLGSSNWEAADLGNEER |
| 3245 | O00193 | RsAsPDDDLGSSNWEAADLGNEER |
| 3246 | O00178 | LHGGFDsDCsEDGEALNGEPELDLTSK |
| 3247 | O00178 | LHGGFDsDCsEDGEALNGEPELDLTSK |
| 3248 | O00161 | TTWGDGGENsPCNVVSK |
| 3249 | O00159 | DVEsPSWR |
| 3250 | E9PAV3 | VQGEAVSNIQENTQTPTVQEEsEEEEVDETGVEVK |
| 3251 | E9PAV3 | VQGEAVSNIQENTQTPTVQEESEEEEVDEtGVEVK |
| 3252 | B2RUZ4 | DGVsLGAVsSTEEASR |
| 3253 | A6NFI3 | AALHTTPDsPAAQLER |
| 3254 | A5YM69 | AEELsPAALsPLLEPIR |
| 3255 | A5YM69 | AEELsPAALsPLLEPIR |
| 3256 | A2RU67 | SPDLGEYDPLTQADsDEsEDDLVLNLQK |
| 3257 | A2RU67 | SPDLGEYDPLTQADsDEsEDDLVLNLQK |
| 3258 | A2AJT9 | HRsLsPVPR |
| 3259 | A2AJT9 | HRsLsPVPR |
| 3260 | A1L390 | ALssEEEEEMGGAAQEPESLLPPSVLDQASVIAER |
| 3261 | A1L390 | ALssEEEEEMGGAAQEPESLLPPSVLDQASVIAER |
| 3262 | A1L390 | SSSVLsLEGSEK |
| 3263 | A1L390 | SSSVLsLEGsEK |
| 3264 | A1L390 | sPLsPTETFSWPDVR |
| 3265 | A1L390 | sPLsPTETFSWPDVR |
| 3266 | A0FGR8 | EPTPSIASDIsLPIATQELR |
| 3267 | A0FGR8 | EPTPSIAsDISLPIATQELR |
| 3268 | A0FGR8 | EPTPsIAsDISLPIATQELR |

1. Xie, P.; Liu, J.; Liao, Z.; Zhou, Q.; Sun, J.; Liu, Z.; Xiong, H.; Wan, H. Profiling the differential phosphoproteome between breast milk and infant formula through a titanium (IV)-immobilized magnetic nanoplatform. *Food chemistry* **2024**, *464*, 141541, doi:10.1016/j.foodchem.2024.141541.
2. Wei, X.; Wen, X.; Zheng, H.; Zhang, Y.; Jia, Q. Facile synthesis of Fe3+ immobilized magnetic polydopamine-polyethyleneimine composites for phosphopeptide enrichment. *Journal of chromatography. A* **2024**, *1719*, 464752, doi:10.1016/j.chroma.2024.464752.
3. Wang, J.Y.; Zhang, X.Y.; Yan, Y.H.; Xuan, R.R. Facile preparation of nitrogen/titanium-rich porous organic polymers for specific enrichment of <i>N</i>-glycopeptides and phosphopeptides. *Analytical Methods* **2024**, *16*, 695-703, doi:10.1039/d3ay02011h.
4. Wang, K.X.; Yu, A.J.; Gao, Y.; Chen, M.; Yuan, H.; Zhang, S.S.; Ouyang, G.F. A nitrogen-doped graphene tube composite based on immobilized metal affinity chromatography for the capture of phosphopeptides. *Talanta* **2023**, *261*, 124617, doi:10.1016/j.talanta.2023.124617.
5. He, Y.T.; Zhang, S.S.; Zhong, C.; Yang, Y.X.; Li, G.R.; Ji, Y.; Lin, Z. Facile synthesis of Ti<SUP>4+</SUP>-immobilized magnetic covalent organic frameworks for enhanced phosphopeptide enrichment. *Talanta* **2021**, *235*, 122789, doi:10.1016/j.talanta.2021.122789.
6. Wang, D.Q.; Huang, J.F.; Zhang, H.R.; Gu, T.J.; Li, L.J. Cotton Ti-IMAC: Developing Phosphorylated Cotton as a Novel Platform for Phosphopeptide Enrichment. *Acs Appl Mater Inter* **2023**, *15*, 47893-47901, doi:10.1021/acsami.3c08697.
7. Chen, J.K.; Wang, B.; Luo, Y.T.; Wang, W.M.; Ding, C.F.; Yan, Y.H. Facile preparation of porphyrin-based porous organic polymers for specific enrichment and isolation of phosphopeptides and phosphorylated exosomes. *Talanta* **2023**, *264*, 124771, doi:10.1016/j.talanta.2023.124771.
8. Wang, H.W.; Tang, R.Z.; Jia, S.C.; Ma, S.J.; Gong, B.L.; Ou, J.J. Monodisperse Ti4+-immobilized macroporous adsorbent resins with polymer brush for improved multi-phosphopeptides enrichment in milk. *Microchim Acta* **2022**, *189*, 405, doi:10.1007/s00604-022-05500-5.


**Disclaimer/Publisher's Note:** The statements, opinions and data contained in all publications are solely those of the individual author(s) and contributor(s) and not of MDPI and/or the editor(s). MDPI and/or the editor(s) disclaim responsibility for any injury to people or property resulting from any ideas, methods, instructions or products referred to in the content.
